# The roles of pre-season immunity, age, viral shedding, and community exposures in shaping influenza household transmission dynamics

**DOI:** 10.1038/s41467-026-76037-x

**Published:** 2026-07-30

**Authors:** Molly K. Sauter, Jackie Kleynhans, Jocelyn Moyes, Meredith L. McMorrow, Florette K. Treurnicht, Orienka Hellferscee, Anne von Gottberg, Nicole Wolter, Amelia Buys, Lorens Maake, Neil A. Martinson, Kathleen Kahn, Limakatso Lebina, Katlego Motlhaoleng, Floidy Wafawanaka, Francesc Xavier Gómez-Olivé, Stefano Tempia, Bryan Grenfell, Cécile Viboud, Kaiyuan Sun, Cheryl Cohen

**Affiliations:** 1 Division of International Epidemiology and Population Studies, Fogarty International Center, National Institutes of Health, Bethesda, MD USA; 2 Department of Ecology and Evolutionary Biology, Princeton University, Princeton, NJ USA; 3 Centre for Respiratory Diseases and Meningitis, National Institute for Communicable Diseases of the National Health Laboratory Service, Johannesburg, South Africa; 4 School of Public Health, Faculty of Health Sciences, University of the Witwatersrand, Johannesburg, South Africa; 5 Influenza Division, Centers for Disease Control and Prevention, Atlanta, GA USA; 6 Influenza Program, Centers for Disease Control and Prevention, Pretoria, South Africa; 7 School of Pathology, Faculty of Health Sciences, University of the Witwatersrand, Johannesburg, South Africa; 8 Perinatal HIV Research Unit, MRC Soweto Matlosana Collaborating Centre for HIV/AIDS and TB, University of the Witwatersrand, Johannesburg, South Africa; 9 DST/NRF Centre of Excellence for Biomedical Tuberculosis Research, University of the Witwatersrand, Johannesburg, South Africa; 10 Johns Hopkins University Center for TB Research, Baltimore, MD USA; 11 MRC/Wits Rural Public Health and Health Transitions Research Unit (Agincourt), School of Public Health, Faculty of Health Sciences, University of the Witwatersrand, Johannesburg, South Africa; 12 MassGenics, Duluth, GA USA; 13 Center for Infectious Disease Research, School of Medicine, Westlake University, Hangzhou, Zhejiang Province China

**Keywords:** Influenza virus, Epidemiology, Epidemiology, Risk factors

## Abstract

Protective immunity against influenza transmission remains poorly understood due to the high prevalence of undetected asymptomatic infections. To address this, we analyze 1518 individuals and 623 infection episodes from a three-year, rural and urban, South African household cohort study that combines pre-season serum collection with twice-weekly virological testing regardless of symptoms. By developing a subtype/lineage-specific household transmission model and incorporating time-resolved viral shedding kinetics for all observed infections, we disentangle household and community exposure, pre-season immunity, and age effects. After adjusting for exposure intensity, pre-season hemagglutination inhibition (HAI) titers ≥1:40 are associated with a reduced infection risk for A(H1N1)pdm09, A(H3N2), and B/Victoria, but not B/Yamagata. However, children exhibit higher susceptibility and longer viral shedding durations than adults across all subtypes/lineages, even after accounting for pre-season HAI titers. Here, we show persistent age-related effects on influenza susceptibility and viral shedding, underscoring the need to explore immune mechanisms beyond HAI-based correlates of protection.

## Abstract (web summary)

Current understanding of influenza transmission dynamics is limited by lack of information on asymptomatic transmission. Here, the authors use data from a household-based cohort study in South Africa to investigate the relative contribution of viral shedding, pre-existing immunity, and demographic factors on influenza transmission.

## Introduction

Refining our understanding of influenza transmission is essential for identifying key risk factors for infection, characterizing protective immunity, and informing prevention strategies to reduce disease burden. Human challenge studies have provided valuable insights into the role of prior immunity and exposure dose on infection risk. Yet, these studies involve healthy volunteers in controlled laboratory settings and may not adequately represent the diverse populations and real-world contexts in which influenza spreads^1,2^. In parallel, studying influenza transmission in real-world settings is challenging due to the acute nature of infection, with viral shedding typically lasting only 3 to 6 days, and a high prevalence of asymptomatic infections^1,3–5^. Consequently, the two observational study designs traditionally used to assess influenza transmission and protective immunity, household cohort studies and case-ascertained household transmission studies, often encounter difficulties in tracking infections and capturing transmission dynamics^6^. Overcoming these challenges through improved study designs remains a priority to enhance our understanding of influenza transmission and inform more effective intervention strategies.

The Prospective Household cohort study of Influenza, Respiratory Syncytial virus, and other respiratory pathogens community burden and Transmission dynamics in South Africa (PHIRST) aims to address these challenges by introducing the collection of pre-season serum samples followed by twice-weekly respiratory virological testing regardless of symptom presence throughout the influenza season^7^. This unique design leverages the distinct strengths of traditional observational studies while overcoming key limitations. Prior household cohort studies have sampled based on symptomatic illness and/or inferred infections from paired sera. Although the paired sera approach can detect asymptomatic and subclinical infections, its sensitivity and specificity are limited, and it lacks precision in determining the timing of infection^6,8–10^. Case-ascertained household transmission studies have relied on clinical visits of symptomatically infected individuals to enroll index cases and their households, neglecting transmission events initiated by asymptomatic individuals and those who do not seek care^5,6,11,12^. In contrast, symptom-independent routine testing in the PHIRST cohort allows for the precise identification of influenza infections and household index cases across the clinical spectrum of disease, eliminating the bias towards symptomatic transmission. Further, the PHIRST cohort enables the assessment of the relationship between pre-existing immunity and the risk of infection^6,8–10^, along with direct observation of transmission events through repeated virological testing at short time intervals^5,6,11^. Specifically, these data provide the means to reconstruct viral shedding kinetics for each influenza-infected participant during the study period, which permits the characterization of time-varying influenza exposure risk for susceptible household members.

In this study, leveraging the PHIRST study’s unique design and detailed data collection in a high-transmission setting, we developed a highly detailed household transmission model to understand how influenza virus exposure intensity, age, and serum HAI titers influence the risk of infection acquisition. We are particularly interested in quantifying the role of prior immunity while accounting for influenza virus exposure, and vice versa. We also provide valuable insights into influenza virus transmission from low- and middle-income countries, where the burden of influenza is heightened by low vaccination rates, population demographics, and a high prevalence of comorbidities^13–16^.

## Results

### Cohort description

The PHIRST participants were young (56% of the individuals were <18 years old) and resided in large households (40% of households had over 5 members). HIV prevalence was high with 16% of participants living with HIV (PLWH) and 19% of PLWH having a CD4 count less than 200 (an indication of immune suppression). No individuals included in this analysis had received an influenza vaccine for the then-current year. Table 1 lists individual and household characteristics for individuals that completed the follow-up period, provided all necessary samples, and had pre-season titer samples analyzed (see ref. ^7^ for a complete cohort profile). Over the three years of the study, there were 1258 influenza-positive specimens, comprising around 1% of all the specimens collected. Among the 1518 individuals included in the analysis, 513 (34%) experienced at least one influenza virus infection episode within the year of the individual’s study enrollment. All four known circulating subtypes/lineages of seasonal influenza were captured over the course of the three-year study. In 2016, the shortest data collection period, the predominant subtypes/lineages were A(H1N1)pdm09 and B/Victoria, though there was some co-circulation of A(H3N2). Conversely, A(H3N2) and B/Yamagata led circulation in 2017. A(H1N1)pdm09 and B/Victoria returned to predominance in 2018 (for sampling results across all years, see Supplementary Figs. 4–6).

**Table 1:** Key characteristics of individuals, households, and influenza viral infections

Individual characteristic	Year		2016 *N* = 480	2017 *N* = 523	2018 *N* = 515
	Site	Agincourt (Rural)	234 (49)	261 (50)	253 (49)
		Klerksdorp (Urban)	246 (51)	262 (50)	262 (51)
	Age group (years)	<5	59 (12)	58 (11)	59 (11)
		5–11	112 (23)	119 (23)	139 (27)
		12–18	103 (22)	110 (21)	85 (17)
		19–40	115 (24)	102 (19)	142 (28)
		>40	91 (19)	134 (26)	85 (17)
	Sex	Female	286 (60)	309 (59)	325 (63)
		Male	194 (40)	214 (41)	190 (37)
	HIV status	Negative	403 (84)	440 (84)	400 (78)
		PLWH CD4 ≥ 200	64 (13)	58 (11)	72 (14)
		PLWH CD4 < 200	13 (3)	10 (2)	22 (4)
		Unknown	0	15 (3)	21 (4)
Household characteristic	Year		2016 *N* = 100	2017 *N* = 108	2018 *N* = 117
	Household size	3–5 Members	51 (51)	61 (56)	82 (70)
		6–10 Members	44 (44)	41 (38)	34 (29)
		>10 Members	5 (5)	6 (6)	1 (1)
Observed infection episodes	Year		2016 *N* = 174	2017 *N* = 239	2018 *N* = 210
	A(H1N1)pdm09		52 (30)	4 (2)	77 (37)
	A(H3N2)		28 (16)	164 (69)	3 (1)
	B/Victoria		90 (52)	0	130 (62)
	B/Yamagata		4 (2)	71 (30)	0

Key characteristics of individuals and households included in the analysis, and influenza virus infections for which shedding kinetics could be estimated. Brackets indicate percentage relative to the given *N* for the year and category (Individual Characteristic/Household Characteristic/Observed Infection Episodes). See ref. ^7^ for all observed individuals and infections. For the HIV status covariate, PLWH stands for persons living with HIV, further defined by demonstrating a CD4 T cell count below or equal to and above 200.

**Fig. 1:**
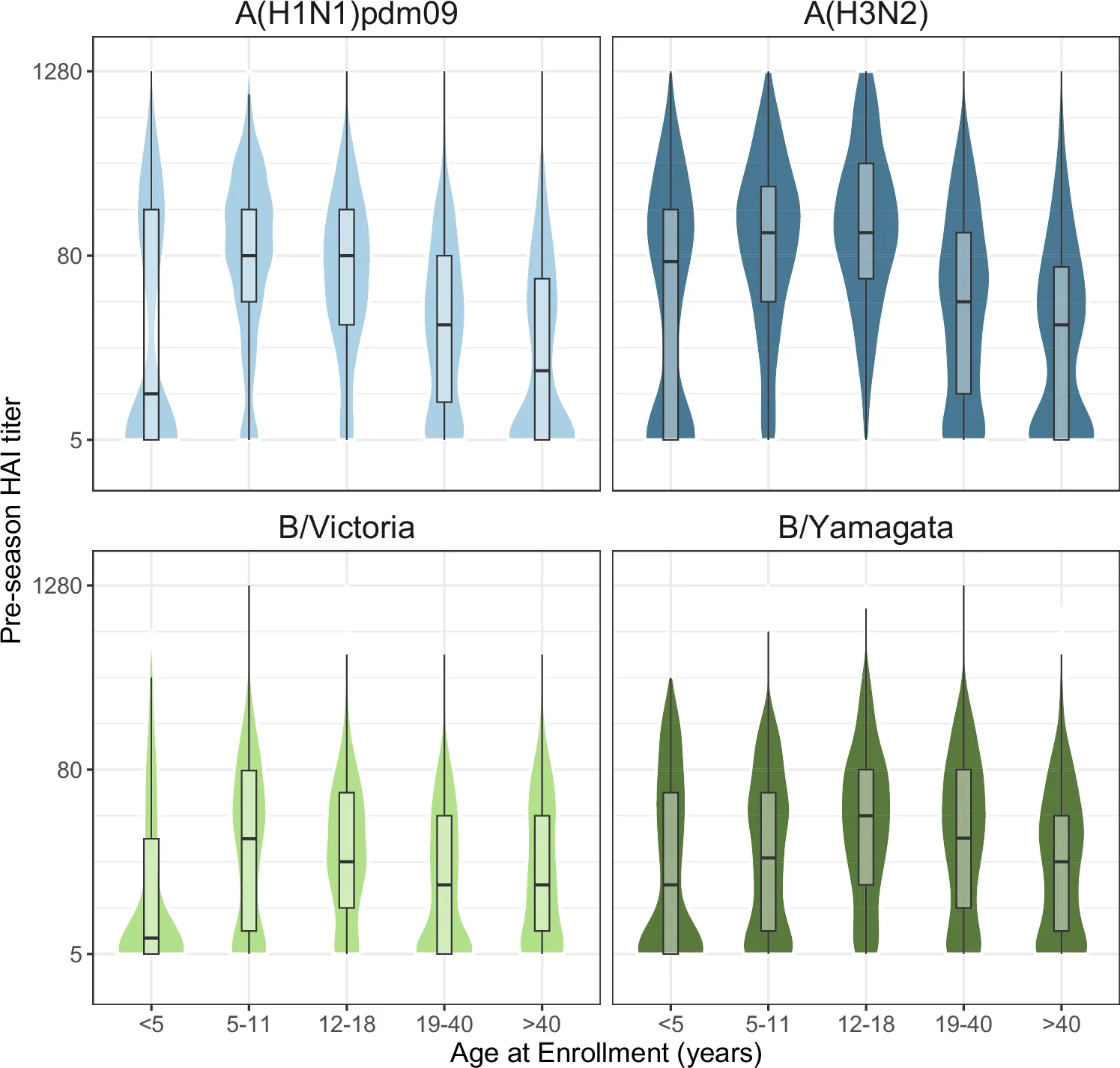
Distribution of pre-season serum influenza antibody titers by age group. Distribution of pre-season titers (in log scale) by age group and influenza subtype/lineage. The titers were generated using hemagglutination inhibition assay (HAI) against representative circulating viruses during the study period. Titers were analyzed for 1518 individuals with the age groups comprising 59 individuals for <5 years old, 112 for 5–11 years old, 103 for 12–18 years old, 115 for 19–40 years old, and 91 for  >40 years old. Violin plots show the distribution of pre-season titers, with the width indicating the density of observations. The overlaid box plots summarize the median with the center black line, the interquartile range defined by the box limits, and depict whiskers extending to 1.5 times the interquartile range for each age group. Source data are provided as a Source Data file.

We examined the distribution of pre-season hemagglutination inhibition (HAI) titers across age groups (Fig. 1). The youngest group (<5 years) displays a distinctly bimodal titer distribution, consistent with the stochastic nature of early-life influenza exposure: many children remain immunologically naïve, while others have already experienced infection and seroconverted. In contrast, individuals aged 12–18 years exhibit a more unimodal, but still broadly distributed, titer profile, reflecting the accumulation of heterogeneous exposure histories over time. Among older adults, the titer distribution reverts to a bimodal pattern, with evidence of seroreversion, potentially indicative of waning immunity due to aging and/or the long-term effects of immune imprinting. Supplementary Fig. 12 illustrates the age-dependent trajectory of average pre-season hemagglutination inhibition (HAI) titers. For both influenza A subtypes, titers increase steeply between ages 0 and 10, whereas influenza B lineages show a more gradual rise over a broader age range, likely reflecting less frequent immune boosting due to lower infection rates, as well as the poorer persistence of antibodies against influenza B viruses compared with those against influenza A. Thereafter, titers for all subtypes and lineages decline progressively with advancing age and eventually plateau in older adulthood at levels of approximately 20–30.

**Fig. 2:**
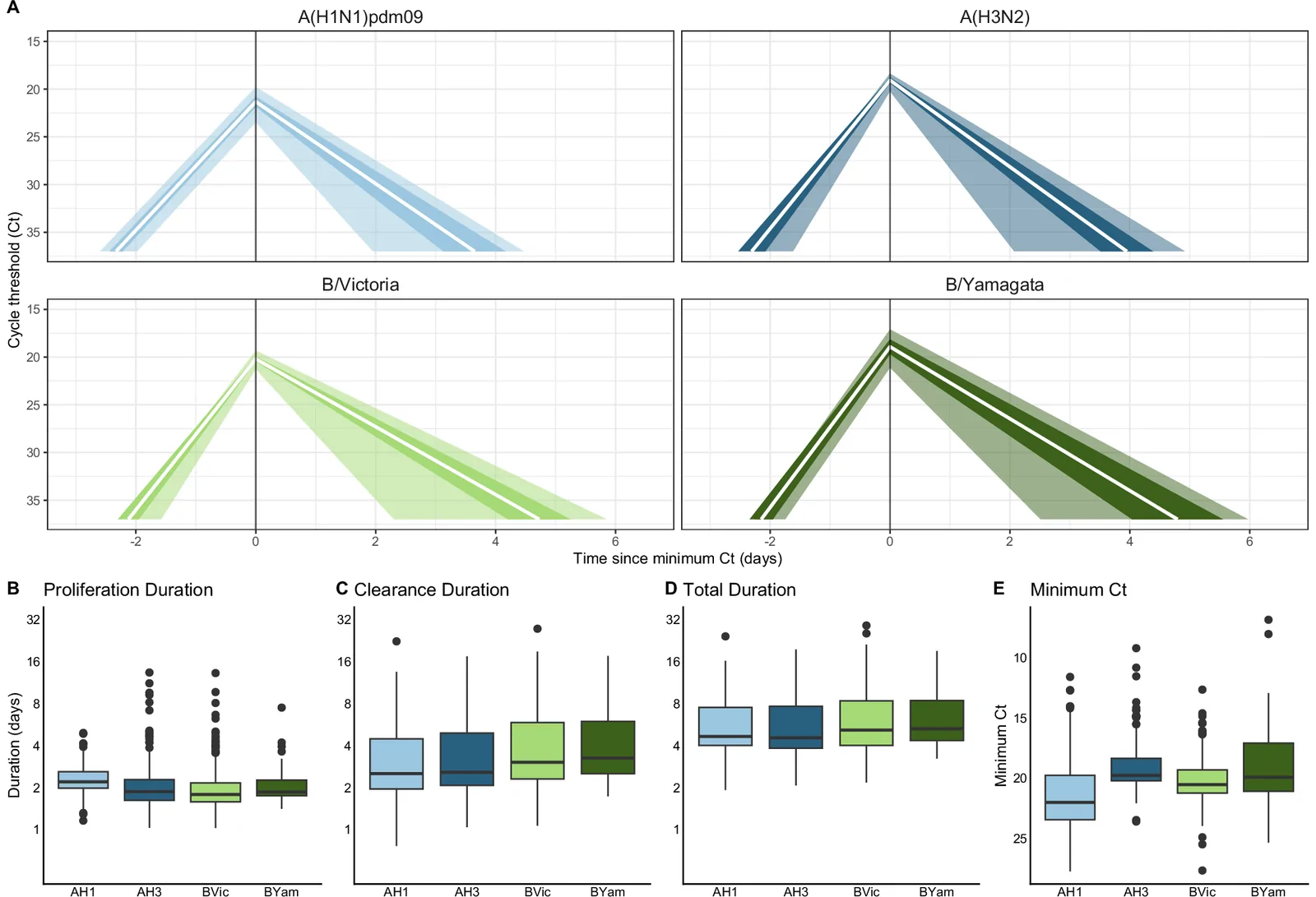
Influenza viral shedding kinetics estimates for each subtype/lineage. Shedding kinetics estimated for 623 total infection episodes: 133 episodes of A(H1N1)pdm09, 195 of A(H3N2), 220 of B/Victoria, and 75 of B/Yamagata. **A** Average estimated viral shedding kinetics in Ct values over time since peak shedding (i.e., time since the minimum Ct value). The white solid line represents the posterior mean estimate from the Bayesian piecewise linear viral shedding model, the darker shaded area represents the 95% confidence intervals, and the lighter shaded area represents the interquartile range. **B** Distributions of the proliferation duration estimates for each subtype/lineage. The box plots summarize the median with the center black line, the interquartile range by the box limits, and include whiskers extending to 1.5 times the interquartile range with points beyond the whiskers as outliers. **C**–**E** same as (**B**) for clearance duration, total duration, and minimum Ct (peak viral load), respectively. Source data are provided as a Source Data file.

Viral RNA shedding trajectories were estimated for 623 infection episodes, of which 133 (21%) were A(H1N1)pdm09, 195 (31%) were A(H3N2), 220 (35%) were B/Victoria, and 75 (12%) were B/Yamagata (Table 1). Figure 2 summarizes the viral shedding kinetics estimates for each subtype/lineage. The median proliferation duration was similar across subtype (Fig. 2B; A(H1N1)pdm09 2.20 days, IQR: 1.98–2.60; A(H3N2) 1.87 days, IQR: 1.62–2.28; B/Victoria 1.78 days, IQR: 1.56–2.16; B/Yamagata 1.86 days, IQR: 1.75–2.26). The median clearance duration was consistently longer than the proliferation duration for all subtypes, and slightly, though not significantly longer, for both type B lineages compared to type A subtypes (Fig. 2C; A(H1N1)pdm09 2.52 days, IQR: 1.95–4.47; A(H3N2) 2.57 days, IQR: 2.07–4.92; B/Victoria 3.04 days, IQR: 2.30–5.86; B/Yamagata 3.26 days, IQR: 2.51–5.97). Accordingly, the total durations were similar across subtypes/lineages (Fig. 2D; A(H1N1)pdm09 4.7 days, IQR: 4.0–75; A(H3N2) 4.6 days, IQR: 3.8–7.7; B/Victoria 5.2 days, IQR: 4.0–8.4; B/Yamagata 5.3 days, IQR: 4.4–8.4). The coefficients of variation were higher for the clearance duration than proliferation duration, significantly so for all but A(H3N2) by asymptomatic testing for equality of coefficients of variation (*p*-value < 0.05)^17^. (Supplementary Table 1). The median estimated peak viral load as proxied by minimum Ct for the episode ranged from 19.8 Ct for A(H3N2) to 22.0 Ct for A(H1N1)pdm09 (Fig. 2E; A(H1N1)pdm09 22.0 Ct, IQR: 19.8–23.5; A(H3N2) 19.8 Ct, IQR: 18.4–20.2; B/Victoria 20.5 Ct, 19.3–21.3; B/Yamagata 19.9 Ct, IQR: 17.1–21.1).

### Age and pre-season HAI titers association with the shedding kinetics of seasonal influenza

**Fig. 3:**
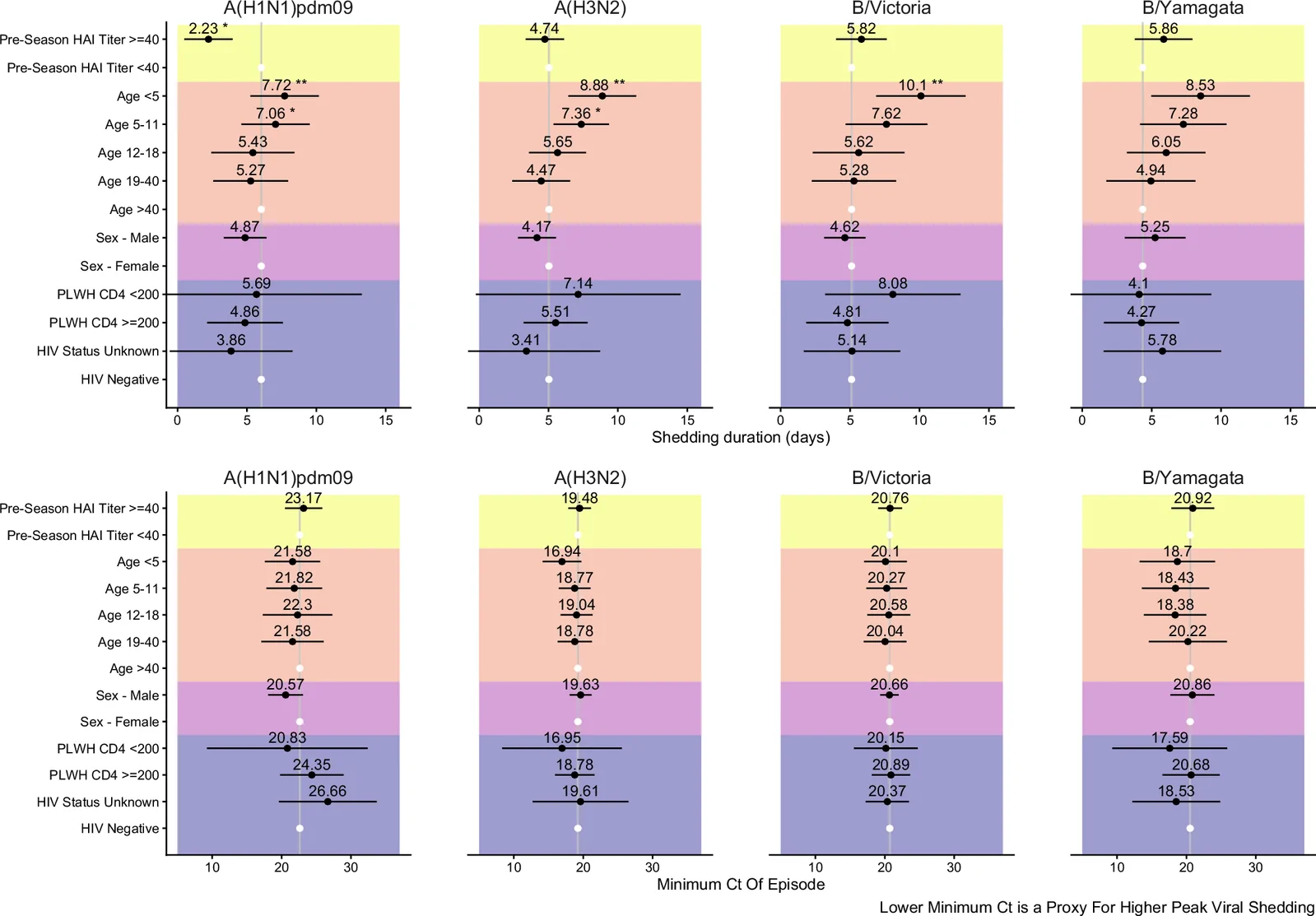
Predictors of influenza virus shedding characteristics by subtype/lineage based on multivariable linear regression. The top panel regresses the total shedding duration and the bottom panel the peak viral load, proxied by the minimum Ct of the infection episode. For the HIV status covariate, PLWH stands for persons living with HIV, further defined by demonstrating a CD4 T cell count below or equal to and above 200. Associations were estimated using multivariable mixed-effects linear regression models fitted separately to 100 posterior-resampled viral shedding trajectories, with parameter estimates pooled across realization using Rubin’s rules, modeled for independent infection episodes as biological replicates. These analyses included 133 infection episodes for A(H1N1)pdm09, 195 for A(H3N2), 220 for B/Victoria, and 75 for B/Yamagata. Statistical inference was based on two-sided Wald tests with Satterthwaite’s approximation for degrees of freedom. No adjustment was made for multiple comparisons. For shedding duration, estimates are given in units of days with lines representing the 95% CI. The central line indicates the mean duration for a given subtype/lineage. The reference groups are designated by a white dot. For the shedding duration, the reference estimates for each subtype are as follows: A(H1N1)pdm09 4.39 days, A(H3N2) 4.71 days, B/Victoria 5.64 days, B/Yamagata 4.94 days. For the minimum Ct of episode, the reference estimates are A(H1N1)pdm09 22.62 Ct, A(H3N2) 21.06 Ct, B/Victoria 20.54 Ct, B/Yamagata 20.72 Ct. Asterisks denote *p*-value: “***”: <0.001 “**”: <0.01 “*”: <0.05. Exact *p*-values for results indicated as significant are as follows: for A(H1N1)pdm09: Pre-Season HAI Titer ≥ 40 *p* = 0.0182, Age <5 *p* = 0.0099, Age 5–11 0.0367; for A(H3N2): Age <5 *p* = 0.0011, Age 5–11 *p* = 0.0105; for B/Victoria: Age <5 *p* = 0.0073. Source data are provided as a Source Data file.

Figure 3 shows predictors of the total duration and the peak viral load (minimum Ct) of viral RNA shedding by influenza subtype/lineage. For A(H1N1)pdm09 infections, higher pre-season HAI titers correlated with shorter durations of shedding, with a 2.3-day reduction in duration time for those with a titer of at least 1:40 compared to those with lower titers [95% confidence interval (CI): −3.90 to −0.41 days]. This was not seen for other subtypes/lineages. Younger individuals tended to shed for longer, even after adjusting for HAI titers. In particular, children <5 years old exhibited a 3–5 day longer shedding duration for all subtypes/lineages apart from B/Yamagata compared to persons >40 years old (A(H1N1)pdm09 3.33 days, 95% CI: 0.87–5.79; A(H3N2) 4.16 days, 95% CI: 1.72–6.61; B/Victoria 4.46 days, 95% CI: 1.24–7.69). Children aged 5–11 years also demonstrated longer durations for infections of A(H1N1)pdm09 [2.67 days, 95% CI: 0.21–5.13] and A(H3N2) [2.64 days, 95% CI: 0.65–4.64]. HIV status, even among the immunocompromised, was not significantly associated with the total duration of shedding for any of the influenza subtypes. Regarding peak viral load, no significant association was found between any of the included covariates and viral load reduction for any subtype or lineage (Fig. 3, bottom panel).

### Household and community influenza exposures, pre-season HAI titers, and age jointly shape the risk of influenza acquisition

**Fig. 4:**
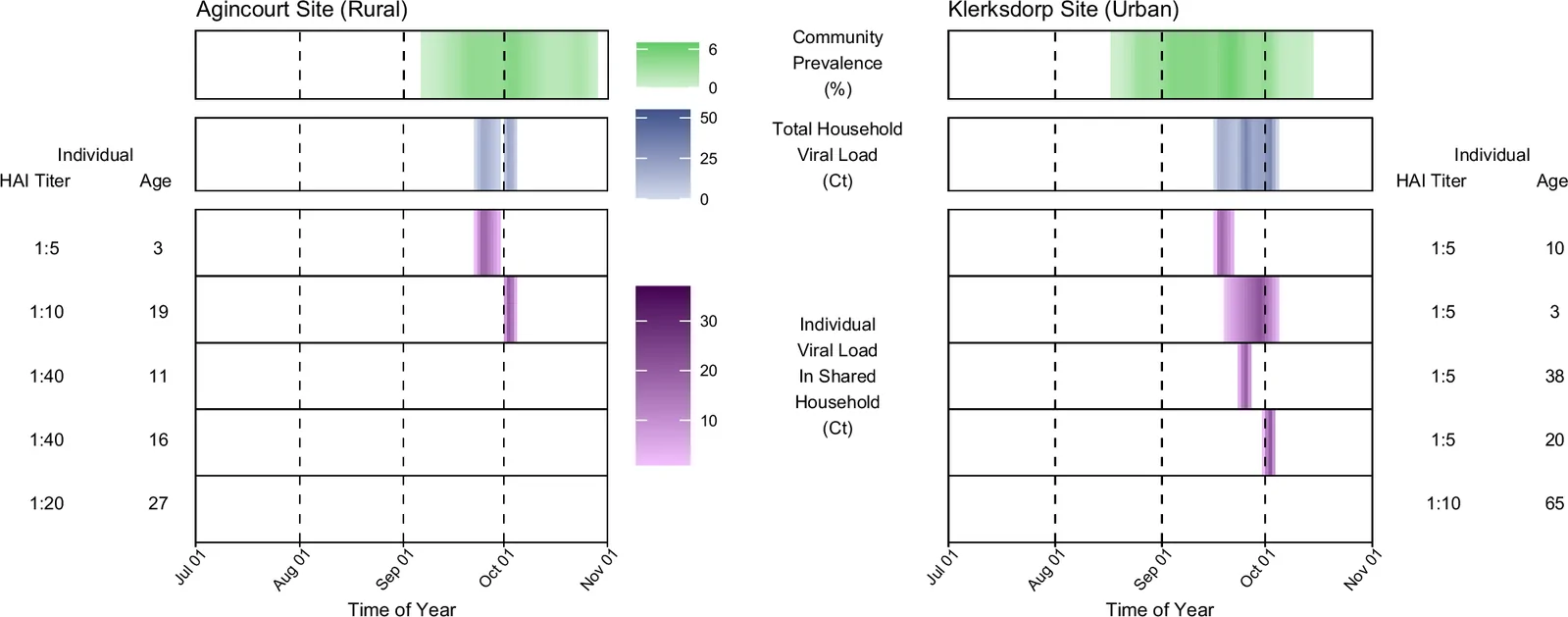
Visualization of key variables of the influenza household transmission model. Depicts the interplay between community prevalence, household exposure, pre-season titers and age. Two individual households, selected from rural (left) and urban (right) communities, are presented, and all data references B/Victoria in 2018. Influenza community prevalence (top panel) is shown as a percentage of the enrolled cohort with an observed PCR-confirmed infection. The total influenza household exposure (second panel) is shown as a composite of individual viral loads Ct values (bottom panel) within the household. The ages (in years) and pre-season HAI titers of household members are indicated on either side. The model is run on a daily time scale. Source data are provided as a Source Data file.

**Fig. 5:**
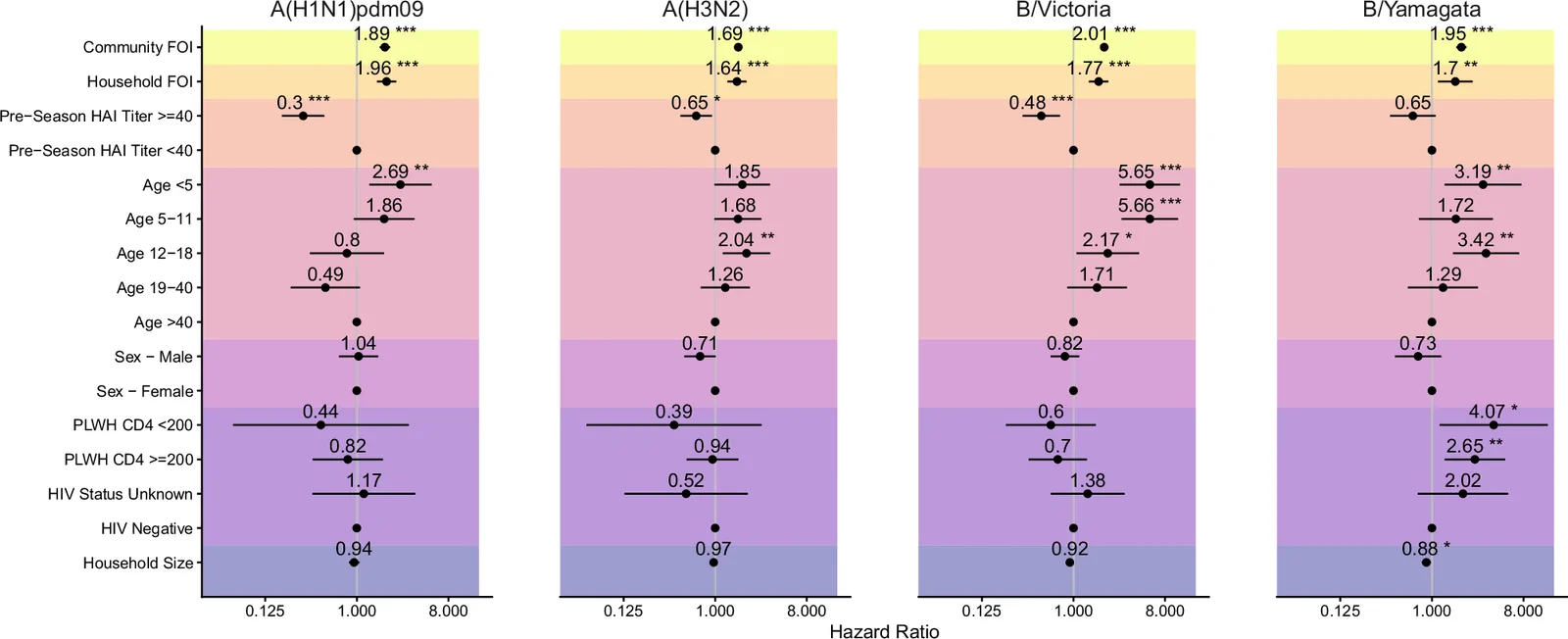
Factors influencing the risk of influenza virus infection acquisition by subtype/lineage based on multivariable logistic regression. Variables include the community force of infection (FOI), household FOI, pre-season HAI titer, age, sex, HIV status, and household size. Community FOI is indicated with respect to a 0.01 increase in absolute community prevalence (defined as the total number of infection episodes within the cohort at a given time/cohort population). Household FOI is indicated with respect to a step by 10 Ct units within the household. For the HIV status covariate, PLWH stands for persons living with HIV further defined by demonstrating a CD4 T cell count below or equal and above 200. Associations were estimated using multivariable Poisson regression models fitted separately to 100 posterior-resampled viral shedding trajectories, with estimates pooled using Rubin’s rules to propagate uncertainty from the shedding model on the timing of infection and time-varying household FOI into the final regression estimates. Statistical inference was based on two-sided Wald tests. Analyses treated individuals as biological estimates and sample sizes included 923 individuals for A(H1N1)pdm09, 500 individuals for A(H3N2), 866 individuals for B/Victoria, and 507 individuals for B/Yamagata. No adjustment was made for multiple comparisons. Black dots represent point estimates of hazard ratios interpreted on the log link scale (Poisson regression is equivalent to a Cox model in this context), with the black horizontal error bars indicating 95% CI intervals; white dots indicate reference categories. The reference groups for categorical variables are designated by a white dot. Asterisks denote *p*-value “***”: <0.001 “**”: <0.01 “*”: <0.05. Exact *p*-values for results indicated as significant are as follows: for A(H1N1)pdm09: Community FOI *p* = 1 × 10^−^^24^, Household FOI *p* = 1 × 10^−^^9^, Pre-Season HAI Titer ≥ 40 *p* = 1 × 10^−^^7^, Age <5 *p* = 0.0061; for A(H3N2): Community FOI *p* = 5 × 10^−^^37^, Household FOI *p* = 6 × 10^−^^6^, Pre-Season HAI Titer ≥ 40 *p* = 0.0186, Age 12–18 *p* = 0.0098; for B/Victoria: Community FOI *p* = 8 × 10^−^^47^, Household FOI *p* = 4 × 10^−^^7^, Pre-Season HAI Titer ≥ 40 *p* = 0.0007, Age <5 *p* = 7 × 10^−^^7^, Age 5–11 *p* = 1 × 10^−^^7^, Age 12–18 *p* = 0.0318; for B/Yamagata: Community FOI *p* = 5 × 10^−^^29^, Household FOI *p* = 0.0079, Age <5 *p* = 0.0092, Age 12–18 *p* = 0.0013, PLWH CD4 < 200 *p* = 0.0249, PLWH CD4 ≥ 200 *p* = 0.0054, Household Size *p* = 0.0158. Source data are provided as a Source Data file.

Next, we used the shedding data to inform a daily transmission model accounting for time-varying influenza exposure in the household. We also considered time-varying infection risk from the community, and demographic and immunological characteristics of household members, using a multivariable logistic regression framework (see Fig. 4 and methods for details on the approach, and Fig. 5 for results). We find a strong effect of the household force of infection (FOI, proxied by viral shedding) on risk of infection acquisition, where a 10 Ct unit increase in viral load within the household associated with a 65–95% increase in risk depending on subtype/lineage (A(H1N1)pdm09 hazard ratio (HR), 1.96 95% CI: 1.58–2.44; A(H3N2) 1.64 HR, 95% CI: 1.32–2.03; B/Victoria 1.77 HR, 95% CI: 1.42–2.20; B/Yamagata 1.70 HR, 95% CI: 1.15–2.52). The community FOI (proxied by influenza infection prevalence in the cohort) was significantly associated with an increase in risk of infection acquisition for all four subtypes/lineages, such that a 0.01 increase in community influenza prevalence in the population, for which the peak prevalence was between 5 and 6%, was associated with a 70–100% increase in risk (A(H1N1) pdm09 1.89 HR, 95% CI: 1.68–2.13; A(H3N2) 1.69 HR, 95% CI: 1.56–1.82; B/Victoria 2.01 HR, 95% CI: 1.83–2.20; B/Yamagata 1.95 HR, 95% CI: 1.74–2.19; Supplemental Fig. 10).

High pre-season HAI titers were protective against infection for all subtypes with the exception of B/Yamagata, with a titer of at least 1:40 providing a 50–70% reduction in risk of infection acquisition (Fig. 5; A(H1N1) pdm09 0.30 HR, 95% CI: 0.18–0.48; A(H3N2) 0.65 HR, 95% CI: 0.45–0.93; B/Victoria 0.48 HR, 95% CI: 0.31–0.74). Even after controlling for the household and community FOI as well as pre-season HAI titers, there was still a residual association between age and risk of infection acquisition. Compared to the reference group of ≥40 years old, children had an increased risk of infection, although the specific age subgroup exhibiting higher susceptibility depended on the subtype. Those <5 years old had an increased risk of infection by 170–460% for all subtypes except A(H3N2) (A(H1N1)pdm09 2.69 HR, 95% CI: 1.33–5.46; B/Victoria 5.65 HR, 95% CI: 2.85–11.21; B/Yamagata 3.19 HR, 95% CI: 1.33–7.65). Those aged 5–11 years demonstrated a significantly increased risk of infection acquisition compared to the reference group for B/Victoria (B/Victoria 5.66 HR, 95% CI: 2.98–10.73); whereas those in the 12–18 year old group had a higher risk for all subtypes but A(H1N1)pdm09 (A(H3N2) 2.04 HR, 95% CI: 1.19–3.49; B/Victoria 2.17 HR, 95% CI 1.07–4.42; B/Yamagata 3.42 HR, 95% CI 1.61–7.23).

For B/Yamagata infections, larger household size was associated with a decreased risk of infection acquisition, such that one additional person in the household contributed about 10% reduction in risk (0.88 HR, 95% CI 0.80–0.98). For B/Yamagata, compared to individuals that were HIV negative, people living with HIV (PLWH) displayed an increased risk of infection acquisition that scaled with the level of immuno-suppression (PLWH CD4 < 200 4.07 HR, 95% CI: 1.20–13.86; PLWH CD4 ≥ 200 2.65 HR, 95% CI: 1.33–5.28). No other measured characteristic was associated with a significant change in risk of infection acquisition (*p*-value > 0.05).

## Discussion

We analyzed data from the PHIRST cohort in South Africa to refine our understanding of the viral, demographic, and immunologic factors that influence influenza virus transmission. The PHIRST study had a unique design with high-frequency real-time reverse transcription polymerase chain reaction (rRT-PCR) sampling at intervals similar to the timescale of influence virus transmission, enabling us to characterize the shedding kinetics of each observed infection episode. In this cohort that comprised a young population with a relatively high prevalence of comorbidities, we demonstrate that age is a stronger predictor of shedding duration than pre-season HAI titers. From the estimated shedding kinetics of household members and viral activity in the cohort, we model the temporal dynamics of influenza exposure within the household and community, both of which are predictive of risk of infection acquisition. Further, we find that pre-season HAI titers are a correlate of protection for the predominant influenza subtypes/lineages (A/H1N1pdm09, A/H3N2, B/Victoria); however, even after adjusting for titers, children and adolescents retained higher susceptibility to influenza for all subtypes/lineages.

Our results indicate that viral shedding intensity among household contacts was predictive of the risk of acquiring influenza infection. Specifically, a 10-unit increase in shedding intensity, as indicated by the Ct value, was associated with approximately a 95% increased risk for A(H1N1)pdm09, 65% for A(H3N2), 80% for B/Victoria, and 70% for B/Yamagata. These findings are consistent with human challenge studies that have demonstrated a correlation between the risk of influenza virus infection acquisition and challenge viral dose^1,2^. In contrast, previous case-ascertained household transmission studies did not observe significant correlations between the viral shedding intensity of the index case and subsequent infectivity^5,12,18^. These studies concentrated on post-symptomatic viral shedding and thereby could not evaluate pre-symptomatic and asymptomatic shedding, as well as exposures to multiple infected household members. Additionally, the PHIRST cohort included a substantial proportion of children, whose influenza infection episodes were significantly longer and exhibited higher peak viral loads compared to adults, after controlling for pre-season HAI titer. The presence of these prolonged shedding episodes provided the opportunity to examine infectivity hazards with a greater temporal variability than prior studies, thereby enhancing the statistical power of our analysis. Similarly, increased prevalence in the general community, estimated from the overall cohort population, accounting for any exposure outside of the household, such as in schools or workplaces, was also strongly correlated with an increased risk of infection acquisition.

Controlling for time-varying influenza exposures within the household and from the general community, we found that HAI titers of at least 1:40 were a correlate of protection against influenza virus infection for all strains except for B/Yamagata, conferring at most 70% protection against A(H1N1)pdm09 and at least 35% protection against A(H3N2). This relationship between pre-existing immunity and risk of infection acquisition aligns well with prior studies that have identified HAI titers of at least 1:40 as providing approximately 50% reduction in risk, even though this cutoff was based on adults with vaccine-mediated immunity, while the PHIRST cohort was focused on natural immunity^19,20^. Titers against B/Yamagata did not appear to confer protection when considering a threshold of 1:40. However, running the same transmission model, with the only difference being to treat titers as a continuous variable rather than ≥1:40 or <1:40 categorical, higher B/Yamagata titers did associate with a reduced infection risk (Supplementary Fig. 8). These results suggest either that the threshold for protection may be higher than 1:40 for this lineage, or that there was a lack of power due to B/Yamagata causing fewer infections than the other subtypes/lineages in our dataset. Interestingly, the effect size for A(H3N2) is substantially smaller than that for A(H1N1)pdm09. When pre-season titers are analyzed as a continuous variable on a log scale, A(H3N2) titers are no longer significantly associated with a reduced risk of infection. Putative explanations include antigenic mismatch between the circulating A(H3N2) strain during the season and the reference strain used to measure pre-existing antibody titers, or the possibility that A(H3N2) requires a higher protective titer threshold to confer the same level of immunity as A(H1N1)pdm09, reflecting antigenic distinctiveness within influenza A subtypes.

A key result of our study was the strong residual effect of age on the risk of infection acquisition, after adjustment for HAI titers. Previous research has indicated that children have a higher risk of infection for both type A and B influenza^14,21^. Compared to older individuals, children are thought to experience higher contact rates, more exposure-prone behavior, and relative immunological naivety beyond that measured by HAI titers. Our data can shed light on the putative contribution of each of these hypotheses. If contact rates and other behavioral risks were the dominant factors in the age variation in infection risk, there should be consistent age relationships across all subtypes, as contact rates and behavior universally affect infection transmission. Kleynhans et al. ^22^ conducted a cross-sectional study within the PHIRST cohort and found that children aged 14–18 years had the highest contact rates of all participants. This could partially explain the heightened risk of infection acquisition observed in individuals aged 12–18 years for A(H3N2), B/Victoria, and B/Yamagata, but it does not support the increased risk seen in those under 11 years old for A(H1N1)pdm09, B/Victoria, and B/Yamagata. While there could be other age-specific factors, such as contact rates and behavioral heterogeneities unaccounted for, our data suggests a potential residual effect of age-specific immune responses. Previous work has suggested that the 1:40 HAI titer cutoff is inaccurate to use as a correlate of protection when evaluating vaccines for children^23^. While our household model accounts for variations in pre-existing HAI titers both in categorical and continuous scales, other age-dependent immune mediators that are not captured by HAI titers likely play a role (Supplementary Figs. 7 and 8). Since children are considered high-transmitters and a key group for vaccination, it will be important to identify correlates of protection against influenza infection that are age-appropriate^21,24^.

We found a significant gradient of influenza virus shedding duration with age across all subtypes/lineages in our adjusted models, in which the youngest children shed the longest. We did not find significant impact of pre-season HAI titer level on shedding duration, except for influenza A(H1N1)pdm09. Since children, nevertheless, shed the longest, it is possible that unobserved pre-existing components of adaptive immune memory, such as non-HAI antibodies, could have reduced the shedding duration. These additional immune functions likely operate on viral clearance, as the coefficient of variation was significantly higher for clearance duration than proliferation duration for all but A(H3N2) (Supplementary Table 1). If immune functions beyond HAI antibodies, such as those targeting neuraminidase, the HA stalk, or other influenza-specific cellular responses, play a more critical role in promoting viral clearance for A(H3N2), B/Victoria, and B/Yamagata infections, while pre-existing HAI antibodies are more effective at preventing infection and correlate more closely with viral control for A(H1N1)pdm09, this could explain the subtype-specific effects observed on shedding duration^25^. This interpretation aligns with the hypothesis that pre-season HAI antibodies primarily act to block infection rather than to reduce viral shedding once infection is established, with the exception of A(H1N1)pdm09, where HAI titers may better reflect protective immune responses involved in limiting viral replication.

Further, this hypothesis could in part explain why there was no significant association between titers of at least 1:40 and peak viral load reduction. But it should be noted that there was also no residual effect of age, and it is also likely that since peak viral load is less directly measurable, it is likely less accurately estimated and further interpretation is limited. More broadly, the endemicity of seasonal influenza implies the number of influenza exposures accumulates with age, and so would immune memory. Overall, immune compartments other than serum HA antibodies, such as neuraminidase or stalk antibodies, influenza-specific B-cell and T-cell memory, or binding antibodies that do not inhibit hemagglutination, may contribute to the prevention of infection acquisition and/or the reduction of viral shedding^2,25–27^.

We also explored other individual and household-level characteristics associated with both the duration of viral shedding and the risk of infection acquisition. Previous studies have indicated that HIV status influences the risk of morbidity and mortality from influenza-related illness^28,29^. While there was a relatively large portion of people living with HIV included in our analysis, most PLWH were on effective treatment, limiting the power of analysis on immunocompromised individuals. We found no significant correlation between HIV status, including PLWH who were immunocompromised, and the duration of viral shedding. However, PLWH had an increased risk of infection acquisition with the B/Yamagata lineage virus. Additionally, for B/Yamagata, individuals living in larger households exhibited a lower risk of infection after controlling for household FOI. This finding is consistent with previous influenza studies that have suggested a dilution effect of household contact, wherein members of larger households experience a reduced per-contact transmission risk compared with those in smaller households^30^. However, this does not necessarily imply a lower overall household infection risk, as we observed that cumulative household infection risk (defined as the fraction of household members infected during a given season) does not vary across households of different sizes (Supplementary Fig. 11).

Our analysis has several limitations. First, despite the extensive rRT-PCR testing scheme, there were still 2 to 3-day intervals between sample collections. This could result in missing very short infection episodes, potentially leading to missed influenza infections. We also did not use quantitative PCRs but rather used the Ct value as an indirect measure of viral load. Second, our analysis assumes that pre-season HAI titer measurements remain constant throughout each influenza season, despite the knowledge that HAI titer might wane over the season. Third, we were unable to evaluate the impact of co-circulating influenza viruses as we excluded instances of coinfection from our analysis. Fourth, our transmission model lacks a specific contact structure, meaning it does not account for heterogeneity in interactions among household members or demographic differences in community contact exposure^31^. As a result, we cannot fully resolve the contribution of behavioral and biological risk factors to age differences in susceptibility, although subtype differences point at possible immunological factors (related to the amount of antigenic drift and number of past exposures associated with each lineage). Fifth, we did not have independent influenza surveillance data from the study sites to infer community exposure, and instead aggregated PCR-identified infections across all studied households as a proxy for community FOI. In contrast, household FOI was measured by a distinct indicator, based on the total daily viral load within a given household inferred from a viral kinetics model applied to each infected member. Overall, we believe the use of distinct FOI indicators for community and household exposures, fairly large number of households studied (*n* = 327), and asynchronicity of individual outbreaks between households (Supplementary Figs. 4–6), likely contributed to disentangling the role of these two infection sources. Another caveat relates to the absence of influenza vaccination in our cohort, limiting the applicability of our findings to settings with greater vaccine usage, although it allows us to focus on the role of natural immunity. Additionally, the number of observed B/Yamagata infections was lower compared to other subtypes limiting the statistical power for this lineage. It is worth noting that globally, the B/Yamagata lineage is now considered extinct in the post-COVID-19 pandemic period^32^. Finally, our study was limited to the pre-COVID-19 period; it remains unclear how the immunity gap produced by the lack of influenza circulation during the COVID-19 pandemic would impact the relationship between pre-existing HAI titers, age, and the risk of infection^10,33,34^.

In summary, by modeling high-resolution virological samples from a detailed cohort study, we were able to reconstruct the time-varying intensities of influenza exposures within households and from the general community, both of which significantly correlated with the risk of influenza infection acquisition. After adjusting for these exposures, we found that pre-season HA antibodies reduced the risk of infection acquisition by influenza A(H1N1)pdm09, A(H3N2), and B/Victoria, but not B/Yamagata and reduced shedding duration for A(H1N1)pdm09. After adjustment for HAI titers, we found a residual effect of age on shedding duration and infection risk. Furthermore, the residual age effects on susceptibility and shedding duration and the inconsistency of the observed age trend between subtypes, highlight the need for more work on the roles of immune compartments beyond serum HA antibodies, such as antibodies to neuraminidase and HA stalk, B-cell, T-cell, neutralization and other effector functions of antibodies as potential correlates of protection against influenza.

## Methods

### Study design

The PHIRST study enrolled participants from two study sites in rural and urban communities of South Africa^7^. The rural site was in the Agincourt sub-district of the Mpumalanga Province Demographic Surveillance Site, an established health and socio-demographic surveillance site (HDSS) since 1992. The urban study site was the Jouberton suburb of Klerksdorp in the North West Province. New cohorts of households were enrolled each year from 2016 to 2018 prior to the start of the winter influenza season, which typically runs from May to September in South Africa’s temperate climate. In Agincourt, households were randomly selected from the HDSS database and in Klerksdorp, selection was based on proximity to randomly generated global positioning system) coordinates. Successful enrollment required that the household had more than 2 members and that at least 80% of the members provided written consent, or written assent and consent from a parental guardian for household members <17 years old. Each year, we enrolled an average of 55 new households, or 281 individuals, per study site. A total of 327 households and 1684 individuals were successfully enrolled and completed follow-up surveys and samples over the duration of the three-year study.

Upon enrollment, we collected demographic, household, and baseline health data, including an offer of HIV testing to all participants who did not have a known HIV status and were over 10 years old; children were tested at birth and, when negative, were assumed to be negative through age 10. Blood serum samples were collected at least once prior to each influenza season. After enrollment, the participants entered an intensive follow-up period for six months in 2016, and ten months in 2017 and 2018, spanning the entirety of the influenza season. Trained investigators visited each household twice-weekly to collect nasopharyngeal swabs regardless of the presence of symptoms or health-seeking behavior.

### Laboratory testing

Influenza antibody titers were measured with HAI assays against strains circulating during the study period: A/South Africa/2517/2016 (A(H1N1)pdm09), A/Singapore/INFIMH-16-0019/2016 (A(H3N2)), B/South Africa/R3037/2016 (B/Victoria), and B/South Africa/R05631/2017 (B/Yamagata). The same viruses were used for all three study years. Nasopharyngeal swabs were tested for influenza with rRT-PCR using the FTD Flu/RSV detection assay (Fast Track Diagnostics, Luxembourg)^7,35^. Positive samples were further analyzed to determine the influenza subtype or lineage with the US CDC influenza A subtyping kit or the CDC influenza B Yamagata and Victoria lineages typing kit (available through International Reagent Resource Program; http://www.internationalreagentresource.org)^7,35^. The cohort design, enrollment, testing, and laboratory protocols have all been described in detail in previous publications^7^.

### Analytical approach

To build a household transmission model that captures the daily risk of influenza virus infection based on the shedding dynamics of infected household members, we first estimated the viral shedding kinetics of each observed infection episode. Episodes were defined by clustering influenza-positive samples (rRT-PCR cycle threshold (Ct) value < 37) that were less than 14 days apart and of the same subtype^3^. Some samples tested positive for an influenza type but did not reach the threshold for positivity on the corresponding subtype or lineage assay. If non-subtyped samples were temporally clustered with samples that could be subtyped, they were assumed to be a part of the same infection episode. We observed limited co-circulation of influenza virus subtypes; there were 25 instances of coinfection (4% of all observed infections), which were excluded from our analysis, as it is challenging to attribute the Ct value accurately to a single subtype in the presence of co-infection.

The rRT-PCR Ct value has been shown to provide a quantitative proxy (though not a direct measure) of a sample’s viral load and has been used in previous studies of influenza transmission^1,5,18^. Based on previous work, we assumed an exponential increase in viral load during the proliferation stage, defined as the period from the onset of shedding to the peak viral load, followed by an exponential decrease during the clearance stage, defined as the period from the peak viral load to the end of shedding^36,37^. This translates to a linear decrease in Ct value from the threshold value of 37 to a minimum Ct value (i.e., proxy peak viral load), followed by a linear increase in Ct from the minimum to detection threshold. Hence, shedding kinetics can be modeled as a piecewise linear function and fitted to serial Ct values of each infection episode, where Ct values are based on the multiplex FTD Flu/RSV detection assay. To calibrate the model, and account for uncertainty due to censoring or missing observations, we implemented a Bayesian hierarchical framework using the Hamiltonian Monte Carlo algorithm to estimate the value and timing of the minimum Ct (a proxy for peak viral load), as well as the durations of the proliferation and clearance phases, both at the population mean for each subtype or lineage and at the individual (per-infection episode) level. We generated 100 resampled realizations of individual shedding parameters based on their posterior distributions. For each realization, we determined the start and end dates of each infection episode to reconstruct individual shedding timelines. Infection onset was defined as one day prior to the first observed shedding day, consistent with viral kinetics reported in human challenge studies^1,2^ (for model equations and details see Supplementary Text). In all subsequent regression analyses, models were fitted separately to each of the 100 resampled shedding trajectories, and the resulting estimates were pooled together to properly propagate uncertainty from the shedding model into the final reported results.

Next, we explored individual factors that are predictive of viral shedding duration and peak viral load (estimated minimum Ct). We used a mixed effects multiple linear regression fit separately to each subtype/lineage and viral kinetics characteristic (total shedding duration and peak viral load) with fixed effects covariates including age, sex, and HIV infection status and year as a random effect; since B/Yamagata only circulated in 2017, year was not included as a random effect. HIV status was included as a covariate given the high prevalence of PLWH in the study population and its potential relevance to influenza transmission dynamics. HAI antibody titers are commonly used serologic markers of protective humoral immunity to influenza infection with a titer cutoff of 1:40 described to provide 50% protection against the infection^19,20,23^. Thus, we also included the participant’s pre-season HAI titer grouped as either above or equal to 1:40 or less than 1:40 as an indicator of pre-season immunity. For the majority of individuals, we used the HAI antibody titer from a serological sample taken closest to the start of the influenza season, between March and May; however, for the 87 (5.7%) individuals that did not have a blood draw in this time period, we interpolated their titer measurement based on the initial serological measurement taken between November and February (method detailed in supplementary materials). The shedding regression models were fit separately to each influenza subtype/lineage.

To assess predictors of influenza virus infection risk, we employed piecewise exponential survival models with time-varying covariates. These models integrated daily estimates of viral shedding intensity, community influenza prevalence, and other relevant covariates^37–39^. The piecewise exponential model was estimated using Poisson regression, with each individual’s daily infection status as the binary outcome and corresponding daily covariates. At the start of the season, all individuals were considered susceptible and were then excluded from the risk set following the onset of infection with a specific subtype or lineage. Although individuals were no longer deemed susceptible after infection onset, their infection status and shedding intensity continued to contribute to the transmission risk for other household members. A time-varying covariate representing the household FOI was calculated by summing the estimated daily shedding intensities of all influenza-infected individuals within the household. We also included a community FOI term, derived from the prevalence of each influenza subtype within the cohort at the corresponding study site and year. This was measured as the daily number of PCR-positive individuals divided by the total study population at the site (see Supplementary Fig. 10). Data for A(H1N1)pdm09 and B/Victoria included observations from both 2016 and 2018, while observations for A(H3N2) and B/Yamagata were restricted to 2017. Additional individual-level covariates included pre-season HAI titer, age, sex, HIV status, and household size. Further methodological details are provided in the Supplementary Text.

### Ethical approval

The PHIRST (Prospective Household cohort study of Influenza, Respiratory Syncytial virus, and other respiratory pathogens community burden and Transmission dynamics in South Africa) protocol was approved by the University of the Witwatersrand Human Research Ethics Committee (Reference 150808) and the US CDC’s Institutional Review Board relied on the local review (#6840). The protocol was registered on http://clinicaltrials.gov on 6 August 2015 (Reference NCT02519803; https://clinicaltrials.gov/ct2/show/NCT02519803). Participants provided individual written consent or assent prior to enrollment and received a grocery store voucher of ZAR25-30 (USD 2–2.5) per visit for their time. (See 45 C.F.R. part 46.114; 21 C.F.R. part 56.114).

### Reporting summary

Further information on research design is available in the Nature Portfolio Reporting Summary linked to this article.

## Supplementary information


Supplementary Information
Reporting Summary
Transparent Peer Review file


## Source data


Source Data


## Data Availability

The PHIRST Cohort Data are available under restricted access as the individual-level data cannot be publicly shared due to ethical restrictions and the potential for identifying included individuals. Access to the individual participant data, and a data dictionary defining each field in the dataset, requires provision of protocol and ethics approval for the proposed use. To request individual participant data access, please submit a proposal to CC (cherylc@nicd.ac.za), who will respond within 1 month of request. Upon approval, data can be made available through a data sharing agreement. Source Data are provided with this paper; these data are aggregate, de-identified and/or model-derived estimates sufficient to reproduce all data-driven figures and tables presented in the manuscript. For the underlying models requiring individual-level participant data, pseudo-data, structured in the same format as the clinical data, can be found at https://github.com/msauter19/phirst-flu-shed-trans. Source data are provided with this paper.

## Code availability

All the statistical analysis for this study and the figures were performed in R (version 4.3.1, RStudio 2023.6.2.561). The corresponding code to reproduce all figures, as well as executable code using pseudo data to validate the viral shedding kinetics model, viral shedding regression models, and household transmission model, can be found at https://github.com/msauter19/phirst-flu-shed-trans.
