# Supplementary material for: The roles of pre-season immunity, age, viral shedding, and community exposures in shaping influenza household transmission dynamics
Source: Nat Commun. 2026 Jul 30;17:9238. doi: 10.1038/s41467-026-76037-x (PMC13526887; doi:10.1038/s41467-026-76037-x)
Supplement: Supplementary file 1 [file 41467_2026_76037_MOESM1_ESM.pdf]

Supplementary text, tables, and figures:

The roles of pre-season immunity, age, viral shedding, and community exposures in shaping influenza household transmission dynamics

Molly K Sauter[1,2], Jackie Kleynhans[3,4], Jocelyn Moyes[3,4], Meredith L McMorrow[5,6], Florette K Treurnicht[3], Orienka Hellferscee[3,7], Anne von Gottberg[3,7], Nicole Wolter[3,7], Amelia Buys[3], Lorens Maake[3], Neil A Martinson[8,9,10], Kathleen Kahn[11], Limakatso Lebina[8], Katlego Motlhaoleng[8], Floidy Wafawanaka[11], Francesc Xavier Gómez-Olivé[11], Stefano Tempia[4,5,6,12], Bryan Grenfell[2], Cecile Viboud[1], Kaiyuan Sun[13,1¶*], Cheryl Cohen[3,4¶*]

[1] Division of International Epidemiology and Population Studies, Fogarty International Center, National Institutes of Health, Bethesda, Maryland, United States of America

[2] Department of Ecology and Evolutionary Biology, Princeton University, Princeton, New Jersey, United States of America

[3] Centre for Respiratory Diseases and Meningitis, National Institute for Communicable Diseases of the National Health Laboratory Service, Johannesburg, South Africa

[4] School of Public Health, Faculty of Health Sciences, University of the Witwatersrand, Johannesburg, South Africa

[5] Influenza Division, Centers for Disease Control and Prevention, Atlanta, Georgia, United States of America

[6] Influenza Program, Centers for Disease Control and Prevention, Pretoria, South Africa

[7] School of Pathology, Faculty of Health Sciences, University of the Witwatersrand, Johannesburg, South Africa

[8] Perinatal HIV Research Unit, MRC Soweto Matlosana Collaborating Centre for HIV/AIDS and TB, University of the Witwatersrand, South Africa

[9] DST/NRF Centre of Excellence for Biomedical Tuberculosis Research, University of the Witwatersrand, Johannesburg, South Africa

[10] Johns Hopkins University Center for TB Research, Baltimore, Maryland, United States of America

[11] MRC/Wits Rural Public Health and Health Transitions Research Unit (Agincourt), School of Public Health, Faculty of Health Sciences, University of the Witwatersrand, Johannesburg, South Africa

[12] MassGenics, Duluth, Georgia, USA

[13] Center for Infectious Disease Research, School of Medicine, Westlake University, Hangzhou, Zhejiang Province, China.

Supplementary Methods

1. Characterizing kinetics of influenza viral shedding

1.1 Defining subtype-specific infection episodes

An infection episode was defined as a series of consecutive positive samples from the same individual for a given influenza subtype or lineage, as previously described by Cohen et al.[1]. Briefly, following the first positive sample, an episode included all subsequent samples until either (i) more than two weeks elapsed between two consecutive positive samples, or (ii) a sample tested positive for a different subtype than the preceding one. Negative samples occurring within an episode were retained, as transient negative results can arise from fluctuations in viral load or variable sample quality.

In some cases, samples tested negative for all subtype-specific primers despite being positive for the broader influenza type A or B primers. When such samples occurred within the timeframe of an established infection episode for a known subtype, they were retained and assigned the same subtype as the surrounding samples. Samples that remained untyped after this correction were assigned a subtype based on the dominant circulating lineage during that season: B/Victoria in 2016, A(H3N2) and B/Yamagata in 2017, and B/Victoria and A(H1N1)pdm09 in 2018. Non-subtyped samples likely reflected low viral RNA concentrations and excluding them could bias population-level shedding parameter estimates. However, due to the co-circulation of A(H3N2) and A(H1N1)pdm09 in 2016, non-subtyped influenza A samples from that year were excluded. This exclusion affected 14 samples, each representing a distinct infection episode.

To characterize the viral shedding kinetics, we modeled the Ct values from the raw serial RT-PCR data for each observed episode. We obtained up to three different Ct values for one sample, one for the detection assay and one for each subtype that was detected in the case of coinfections. Though the Ct values of the assays typically correlate with each other, the different assays had different calibrations and cannot be directly compared. Generally, it is assumed that the subtype assay is more sensitive to the true RNA concentration in the sample, as it more precisely identifies the present RNA. However, given our interest of including positive non-subtyped samples for the analysis (those samples that tested negative and were typed by the detection assay,

but had a Ct value over the threshold on the subtype assays) all the raw Ct data observations were taken from the FTD Flu/RSV detection assay (Fast Track Diagnostics, Luxembourg) [2,3].

1.2 Modeling Viral Shedding Kinetics Using a Hierarchical Bayesian Framework

As previously described by Kissler et al [4,5], this viral shedding model assumes that the proliferation phase, from the onset of infection to the peak viral load, can be represented by an exponential increase in viral RNA concentration, and that the subsequent clearance phase, from the peak to the end of infection, follows an exponential decline. Consequently, cycle threshold (Ct) values measured by RT-PCR, which serve as a proxy for the negative logarithm of viral RNA concentration, are expected to decrease linearly from the limit-of-detection threshold (Ct_{lod}=37) to a minimum Ct value (Ct_{min}, representing peak viral RNA concentration) during the proliferation phase, and then increase linearly back to Ct_{lod} during the clearance phase. For convenience, we define

$$\Delta\mathrm{Ct}(t) = \mathrm{Ct}(t) - \mathrm{Ct}_{\mathrm{lod}}$$

which represents viral RNA shedding intensity over time, proportional to the logarithm of viral RNA load. The peak shedding intensity is therefore given by

$$\Delta\mathrm{Ct}_{\mathrm{max}} = \mathrm{Ct}_{\mathrm{lod}} - \mathrm{Ct}_{\mathrm{min}}$$

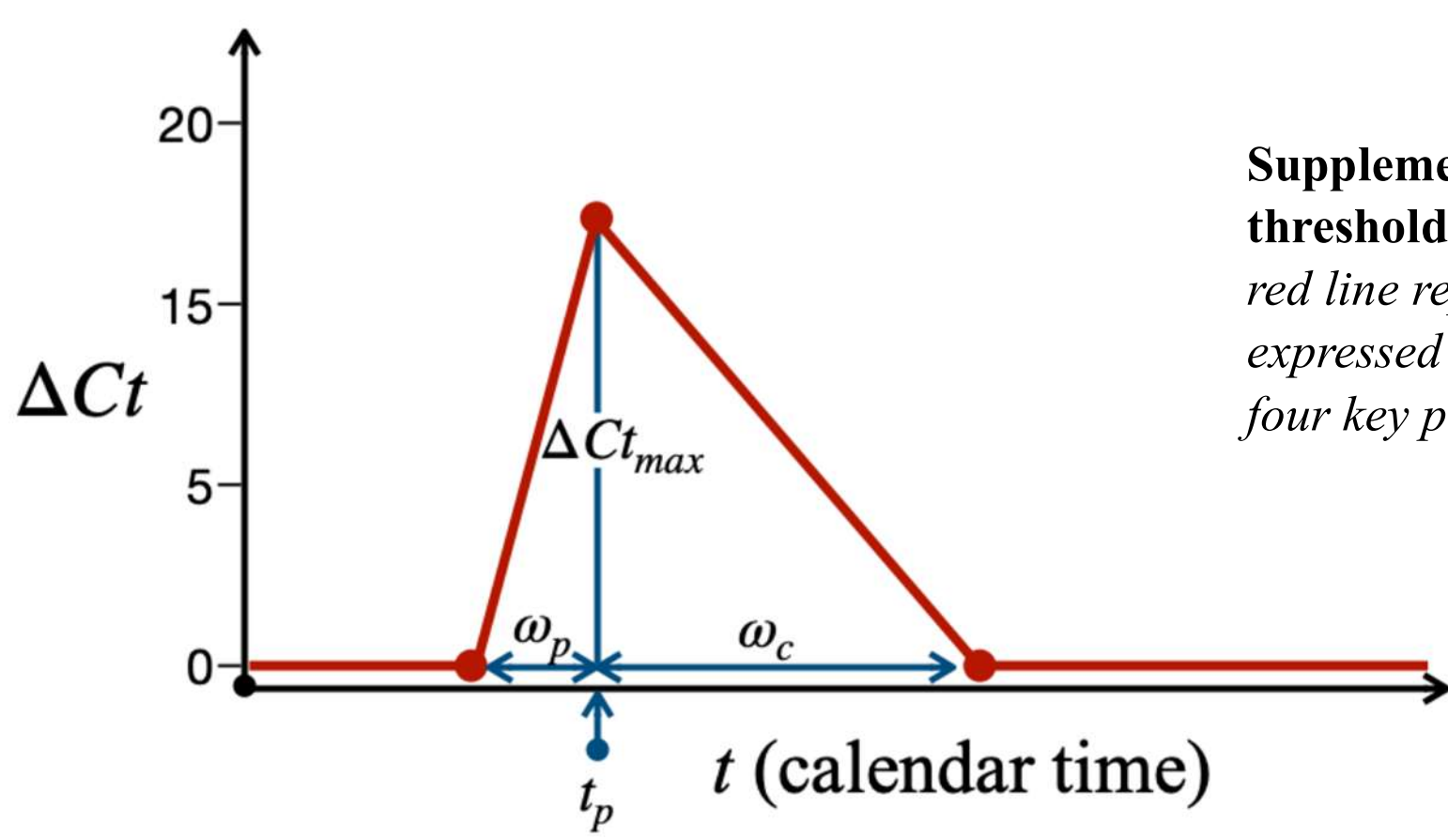

Supplementary Fig. 1. Theoretical trajectory of cycle threshold (Ct) values during an infection episode. *The red line represents the modeled viral shedding trajectory, expressed as $\Delta Ct(t)$ over time. The blue lines indicate the four key parameters estimated in the model:*

Under these assumptions, individual viral shedding kinetics can be described as a linear increase in ΔCt from 0 to the peak shedding intensity (ΔCt_{max}) during the proliferation phase, followed by a linear decrease from ΔCt_{max} back to 0 during the clearance phase. Supplementary Fig. 1 illustrates this theoretical kinetic profile, representing the model expectation $E[\Delta Ct(t)]$, and visualizes the key parameters estimated in the shedding model: ω_p for the proliferation

duration, ω_c for the clearance duration, t_p for the calendar timing of peak viral shedding, and ΔCt_{max} for the peak shedding intensity. Under this viral shedding framework, the expected individual shedding trajectory $E[\Delta Ct(t)]$ can thus be expressed as a set of piecewise linear equations.

$$E[\Delta\mathrm{Ct}(t)] = \begin{cases} 0, & t \le t_\mathrm{p} - \omega_\mathrm{p} \\ \frac{\Delta\mathrm{Ct}_\mathrm{max}}{\omega_\mathrm{p}}(t + \omega_\mathrm{p} - t_\mathrm{p}), & t_\mathrm{p} - \omega_\mathrm{p} < t \le t_\mathrm{p} \\ \Delta\mathrm{Ct}_\mathrm{max} - \frac{\Delta\mathrm{Ct}_\mathrm{max}}{\omega_\mathrm{c}}(t - t_\mathrm{p}), & t_\mathrm{p} < t \le t_\mathrm{p} + \omega_\mathrm{c} \\ 0, & t > t_\mathrm{p} + \omega_\mathrm{c} \end{cases}$$

We then specified an observation model, assuming that the observed $\Delta Ct(t)$ values are drawn from a zero-truncated normal distribution with mean $E[\Delta Ct(t)]$ and standard deviation σ, where σ represents the observational noise shared across all infection episodes of a given subtype.

$$\Delta\mathrm{Ct}(t) \sim \max\big(0, \mathrm{Normal}(E[\Delta\mathrm{Ct}(t)], \sigma)\big)$$

This single parameter was used to absorb two sources of uncertainty that are not separately identifiable from the available data: assay-level variation in quantitative RT-PCR measurements and sampling-level variation during specimen collection. The latter reflects the fact that, even in an actively infected individual, a nasopharyngeal swab may collect different amounts of viral material, or occasionally fail to sample virus-containing tissue, particularly when infection is spatially localized in the upper respiratory tract. Thus, σ should be interpreted as the combined uncertainty in observed viral-load measurements conditional on an infection episode being modeled, rather than as the background false-positive probability of RT-PCR testing.

To avoid applying this observation model to periods in which individuals were unlikely to be infected, the model was fitted only to measurements within the episode window, defined as the identified infection episode together with a two-week buffer period on either side. Negative RT-PCR measurements far from any positive infection episode were not included in the viral-kinetics likelihood and were treated as true negatives for the purpose of defining infection episodes and fitting windows.

For each of the individual parameters (ω_p, ω_c, and ΔCt_{max}), excluding t_p which does not represent intrinsic biological process of viral replication, we implemented a Bayesian hierarchical model at the population level to constrain individual estimates within biologically plausible ranges.

Specifically, each individual parameter was assumed to be drawn from a population-level lognormal distribution, defined as follows:

$$\ln(\omega_{\mathrm{p}}) \sim \mathrm{Normal}\left(\ln\left(\mu_{\omega_{\mathrm{p}}}\right), \ln\left(\sigma_{\omega_{\mathrm{p}}}\right)\right)$$
$$\ln(\omega_{\mathrm{c}}) \sim \mathrm{Normal}\left(\ln\left(\mu_{\omega_{\mathrm{c}}}\right), \ln\left(\sigma_{\omega_{\mathrm{c}}}\right)\right)$$
$$\ln(\Delta\mathrm{Ct}_{\mathrm{max}}) \sim \mathrm{Normal}\left(\ln\left(\mu_{\Delta\mathrm{Ct}_{\mathrm{max}}}\right), \ln\left(\sigma_{\Delta\mathrm{Ct}_{\mathrm{max}}}\right)\right)$$

where $\ln(\cdot)$ denotes the natural logarithm function. To improve computational efficiency, we constrained the parameters to biologically plausible upper bounds: $\omega_{\mathrm{p}} < 15$ days, $\omega_{\mathrm{c}} < 30$ days, and $\Delta\mathrm{Ct}_{\mathrm{max}} < 37$. Both human influenza challenge and household cohort studies have suggested the high end of shedding duration to fall around 8-10 days [6–8], though it has also been shown in A(H1N1)pdm09 that particularly severe infections might shed for over 27 days [9]. Thus, the constraints we used extend well beyond the typically observed durations to accommodate unusually long shedding episodes, while remaining computationally tractable.

The priors of each of the population level mean parameters ($\mu_{\omega_{\mathrm{p}}}$, $\mu_{\omega_{\mathrm{c}}}$, $\mu_{\Delta\mathrm{Ct}_{\mathrm{max}}}$) were specified as truncated normal distributions covering biologically plausible ranges of variation in viral proliferation duration (ω_{p}), clearance duration (ω_{c}), and peak viral shedding ($\Delta\mathrm{Ct}_{\mathrm{max}}$). These priors were informed by human challenge studies and household cohort studies [6–8]. In particular, we drew on a comprehensive review of 56 influenza challenge studies, which provides a consistent characterization of early viral shedding dynamics under controlled conditions [6]. Across the studies, viral shedding increased rapidly within the first day after challenge, peaked around day 2, and had an average duration of approximately 4.8 days with no significant variation between subtypes/lineages. The consistency in the proliferation stage across studies and subtypes/lineages motivated a prior mean of 2 days and prior variance of 1 day for the proliferation phase. However, the same review reported substantial variation in shedding clearance duration between studies and individuals within the same studies, which informed our choice of a relatively large variance of 3 days for the clearance phase. Further, challenge studies are restricted to healthy adults whereas household cohort studies suggest that in the excluded populations, particularly children, viral shedding can be longer and more heterogeneous [7,8]. Such studies also reported that influenza B viruses tend to exhibit slightly longer shedding durations than influenza A viruses. To account for these considerations, we specified priors for the mean clearance duration centered at 4 days for influenza A subtypes and 5 days for influenza B lineages. These priors were intended to be weakly

informative, providing biologically plausible starting values while allowing the observed data to drive posterior inference.

The prior on the maximum change in Ct value reflects the dynamic range of the RT-PCR assay and provides a weakly informative constraint on peak shedding intensity.

Specifically, the priors were:

$$\text{Prior}\left(\mu_{\omega_\mathrm{p}}\right) \sim \text{Normal}(2, 1), \mu_{\omega_\mathrm{p}} > 0$$

$$\text{Prior}\left(\mu_{\omega_\mathrm{c}}\right) \sim \begin{cases} \text{Normal}(4, 3) \text{ for influenza type A}, \mu_{\omega_\mathrm{c}} > 0 \\ \text{Normal}(5, 3) \text{ for influenza type B}, \mu_{\omega_\mathrm{c}} > 0 \end{cases}$$

$$\text{Prior}\left(\mu_{\Delta\mathrm{Ct}_\mathrm{max}}\right) \sim \text{Normal}(37/2, 37/6), \mu_{\Delta\mathrm{Ct}_\mathrm{max}} > 0$$

The priors for the corresponding population-level variation parameters ($\sigma_{\omega_\mathrm{p}}$, $\sigma_{\omega_\mathrm{c}}$, $\sigma_{\Delta\mathrm{Ct}_\mathrm{max}}$) are selected to reflect plausible ranges of biological variability observed across individuals:

$$\text{Prior}\left(\sigma_{\omega_\mathrm{p}}\right) \sim \text{Normal}(1, 1/2), \sigma_{\omega_\mathrm{p}} > 0$$

$$\text{Prior}\left(\sigma_{\omega_\mathrm{c}}\right) \sim \text{Normal}(3, 3/2), \sigma_{\omega_\mathrm{c}} > 0$$

$$\text{Prior}\left(\sigma_{\Delta\mathrm{Ct}_\mathrm{max}}\right) \sim \text{Normal}(37/6, 12), \sigma_{\Delta\mathrm{Ct}_\mathrm{max}} > 0$$

The peak timing (t_p) of each individual shedding episode was estimated independently at the individual level, as it is primarily influenced by individual's exposure to household and community forces of infection rather than by intrinsic within-host viral replication dynamics. Specifically, the prior distribution for each individual's peak timing was assumed to follow a normal distribution centered on the timing of their observed highest ΔCt value, with a standard deviation of 2 days. This choice reflects the empirical sampling frequency of approximately 2–3 days between consecutive respiratory sample collections, allowing sufficient flexibility for the model to capture inter-individual variation in peak timing while remaining consistent with the temporal resolution of the data.

$$\text{Prior}\left(t_\mathrm{p}\right) = \text{Normal}\left(t_\mathrm{p}^{obs}\left(\Delta\mathrm{Ct}\left(t_\mathrm{p}^{obs}\right) = \Delta\mathrm{Ct}_\mathrm{max}^{obs}\right), 2\right)$$

Given the above model specification, we excluded certain infection episodes from the shedding model estimates due to the proximity of missing samples. This included if there were two missing samples prior to the first positive sample or after the last positive sample of an episode. In other words, that we did not have data for a week on either end of the infection, inhibiting the opportunity to estimate the duration with accuracy. We also excluded infections that were cut off by either the start or the end of the collection period, with a positive occurring within one negative

or missing sample from start or end collection date; this was mostly of the B/Victoria wave that occurred relatively late in 2018. This excluded 58 out of 681 infections.

The Bayesian hierarchical models described above were implemented in Stan (rstan version 2.32.6) and interfaced through R (version 4.3.1, RStudio 2023.6.2.561). Separate models were fitted for each influenza subtype or lineage using four Markov Chain Monte Carlo (MCMC) chains, each run for 1,000 iterations. The estimated σ for each influenza subtype reflected the typical deviation of observed data from model predictions (A(H1N1)pdm09 median = 0.65, 95% CI: 0.60–0.73; A(H3N2) median = 1.00, 95% CI: 0.96–1.05; B/Victoria median = 1.25, 95% CI: 1.14–1.39; B/Yamagata median = 0.96, 95% CI: 0.89–1.30). Bayesian modeling of influenza viral shedding was performed using Stan, and posterior summaries and model diagnostics were obtained through the rstan package.

For downstream regression analyses (described in the next section), we accounted for uncertainty arising from potential censoring and missing observations by resampling from the posterior distributions. Each regression was repeated 100 times, drawing one random posterior estimate per individual from the shedding model outputs, and the resulting regression estimates were pooled to obtain the final reported values.

A note on the shedding model ill-fitted estimates

To evaluate the model fit, we analyzed the number of divergent transitions for each of the four model runs, relevant to the numerical instability in Hamilton Monte Carlo sampling and should ideally be zero, and the Gelman R-hat statistic for each posterior, a convergence diagnostic where values close to 1 indicate good mixing across chains. While the majority of fits demonstrated satisfactory convergence and stability, across all model runs there were no divergent transitions, a subset exhibited mild deviations from ideal diagnostic criteria.

At the population level, 3 (36%) of the 24 posteriors had a Gelman R statistic that exceeded the 1.1 threshold. These were for the proliferation mean posteriors for A(H3N2), and both the proliferation mean and clearance posteriors for B/Victoria, which might have been a result of the relatively large observed variation in duration. At the individual-episode level (n = 623), 15 (2%) of minimum Ct posteriors, 43 (7%) of proliferation duration posteriors, 64 (10%) of clearance duration posteriors, and 155 (25%) of peak timing (t_p) estimates had R values above 1.1. The t_p parameter showed the highest frequency of poor convergence, and episodes with poorly estimated

t_p values almost always coincided with ill-fitting estimates of other individual-level parameters. These problematic t_p fits were typically associated with infection episodes containing only a single positive sample, particularly when the Ct value was relatively high (indicating low viral load). Such weak or short-duration infection episodes frequently produced bimodal posterior distributions for t_p, reflecting greater uncertainty in peak timing. For type B influenza, it could also be the case that there is true biological variation as previous work has suggest that the viral shedding kinetics for type B influenza follow a highly variable and occasionally bimodal pattern [7,8].

Supplementary Fig. 2 visualizes a subset of the modeled viral shedding trajectories for five infection episodes for each subtype/lineage. These examples encompass the range of model performance, including two well-converged fits (R-hat = 1), one moderate fit (1.1 < R-hat < 1.5), and two poor fits (R-hat > 1.5). The poorly converged examples display distinctly bimodal, and in some cases trimodal, posterior estimates of the peak time parameter (t_p). Supplementary Fig. 3 presents the corresponding trace plots of the model posterior specifically for t_p, illustrating the sampling behavior underlying these convergence patterns. The bimodal and multimodal posterior distributions seen for these individuals arise primarily resulted from single-sample episodes, where insufficient temporal information constrained the model's ability to define a clear infection trajectory. It is also possible that in some cases, there was true biological variation; type B infections have previously been shown to exhibit bi- or multiphasic viral shedding kinetics [7].

Although such multimodality increases uncertainty in t_p estimation and may reduce apparent model fit, the posterior mean typically falls between alternative peaks, providing a reasonable approximation of the overall infection course. The broader characteristics of the shedding profile, including total duration and magnitude, remain well represented, even in these uncertain cases. Small inaccuracies in peak timing could, however, introduce modest variability in downstream analyses that use t_p to inform household-level force-of-infection (FOI) estimates. To incorporate this uncertainty, both the shedding characteristic regressions and the household transmission model regression analyses used 100 realizations of resampled posterior shedding trajectories across all subtypes, ensuring robust inference despite variable convergence quality.

1.3 Visualizing the estimated shedding kinetics in the main text

In Figure 2A and 2E, when visualizing the average viral shedding kinetics for each subtype or strain, we first transformed the model-predicted ΔCt values back to Ct by adding the limit of detection threshold (Ct_{lod}) to enable direct comparison with the observed RT-PCR data. We then rescaled the temporal axis from calendar time (t) to time since peak shedding (τ), defined relative to the average timing of minimum Ct (i.e., the average timing of peak viral shedding, $\overline{t_P}$). These transformations are defined as follows:

$$\mathrm{Ct}(\tau) = \Delta\mathrm{Ct}(\tau) + \mathrm{Ct}_{\mathrm{lod}}$$

$$\tau = t - \overline{t_P}$$

1.4 Regression analysis of risk factors associated with viral shedding duration and peak viral load

To examine factors associated with the total duration of viral shedding (defined as the sum of viral proliferation and clearance stages) and peak viral load, for each of the 100 realizations of the resampled posterior shedding trajectories, we used mixed-effects multivariable linear regression models.

The covariates included in the models were pre-season HAI titer (categorized as <1:40 or ≥1:40), age, sex, and HIV infection status. These variables were selected from a larger pool of potential covariates based on their improvement of model fit, as assessed by the Akaike Information Criterion (AIC), or their relevance to the study objectives (Table S2). For the pre-season HAI titer, we used the titer from a serological sample taken closest to the start of the influenza season. This collection period occurred between March and May and is depicted for each year in Supplementary Figs. 4-6. There were 87 individuals (5.7%) that did not have blood collected in this time period, but were sampled previously between November and February. Thus, a titer measurement could be interpolated while accounting for potential waning. To do so, for individuals that had both a draw in November-February and March-May, we linearly regressed the March-May titer draw against the November-February draw as well as age, sex, and HIV status of the individual as those individual characteristics created the best fit transmission model (for model testing see Supplementary Tables 2 and 3). We then used this regression to infer the March-May draw based on the November-February draw for those missing this second draw.

To account for potential batch effects across study years, we included study year as a random effect in the regression model. This approach allows the model to estimate a separate

intercept for each year while simultaneously estimating the variance of these intercepts across years. In doing so, the model captures unobserved year-to-year variability without assuming that such differences systematically influence the relationship being tested, for example due to differences in measurement or sample collection. For B/Yamagata, which circulated in only a single study year, inclusion of a random effect for year was not possible; therefore, a standard multivariable linear regression model was used instead. All reported p-values correspond to two-sided tests, with statistical significance defined as $p < 0.05$.

The specific equations of the regression models are as follows:

$$D_i = \beta_0^{\mathrm{D}} + \beta_{\mathrm{T}}^{\mathrm{D}} x_i^{\mathrm{T}} + \boldsymbol{\beta}_{\mathbf{A}}^{\mathbf{D}} \boldsymbol{x}_{\boldsymbol{i}}^{\mathbf{A}} + \boldsymbol{\beta}_{\mathbf{S}}^{\mathbf{D}} \boldsymbol{x}_{\boldsymbol{i}}^{\mathbf{S}} + \boldsymbol{\beta}_{\mathbf{HIV}}^{\mathbf{D}} \boldsymbol{x}_{\boldsymbol{i}}^{\mathbf{HIV}} + b_{\mathrm{Y}}^{\mathrm{D}} x_i^{\mathrm{Y}}$$

$$P_i = \beta_0^{\mathrm{P}} + \beta_{\mathrm{T}}^{\mathrm{P}} x_i^{\mathrm{T}} + \boldsymbol{\beta}_{\mathbf{A}}^{\mathbf{P}} \boldsymbol{x}_{\boldsymbol{i}}^{\mathbf{A}} + \boldsymbol{\beta}_{\mathbf{S}}^{\mathbf{P}} \boldsymbol{x}_{\boldsymbol{i}}^{\mathbf{S}} + \boldsymbol{\beta}_{\mathbf{HIV}}^{\mathbf{P}} \boldsymbol{x}_{\boldsymbol{i}}^{\mathbf{HIV}} + \boldsymbol{b}_{\mathbf{Y}}^{\mathbf{P}} \boldsymbol{x}_{\boldsymbol{i}}^{\mathbf{Y}}$$

Where:

D_i – shedding duration for infection episode i, scalar term.

P_i – peak shedding intensity for infection episode i, scalar term.

β_0^{D} – regression intercept for shedding duration, scalar term.

β_0^{P} – regression intercept for peak shedding intensity, scalar term.

$\beta_{\mathrm{T}}^{\mathrm{D}}$ – regression coefficient for the association between pre-season HAI titer and shedding duration, scalar term.

$\beta_{\mathrm{T}}^{\mathrm{P}}$ – regression coefficient for the association between pre-season HAI titer and peak shedding intensity, scalar term.

x_i^{T}– individual's pre-season HAI titer for infection episode i, scalar term.

$\boldsymbol{\beta}_{\mathbf{A}}^{\mathbf{D}}$ – regression coefficients for the association between age group and shedding duration, vector term, with each element of the vector representing the coefficient of a specific age group.

$\boldsymbol{\beta}_{\mathbf{A}}^{\mathbf{P}}$ – regression coefficients for the association between age group and peak shedding intensity, vector term, with each element of the vector representing the coefficient of a specific age group.

$\boldsymbol{x}_{\boldsymbol{i}}^{\mathbf{A}}$– individual's age categories for infection episode i, vector term, with each element of the vector representing a specific age category.

$\boldsymbol{\beta}_{\mathbf{S}}^{\mathbf{D}}$ – regression coefficients for the association between sex and shedding duration, vector term, with each element of the vector representing the regression coefficient of a specific sex category.

$\boldsymbol{\beta}_{\mathbf{S}}^{\mathbf{P}}$ – regression coefficients for the association between sex and peak shedding intensity, vector term, with each element of the vector representing the regression coefficient of a specific sex category.

$\boldsymbol{x}_{i}^{\mathbf{S}}$– individual's sex categories for infection episode i, vector term, with each element of the vector representing a specific sex category.

$\boldsymbol{\beta}_{\mathbf{HIV}}^{\mathbf{D}}$ – regression coefficients for the association between HIV status and shedding duration, vector term, with each element of the vector representing the regression coefficient of a specific HIV infection status.

$\boldsymbol{\beta}_{\mathbf{HIV}}^{\mathbf{P}}$ – regression coefficients for the association between HIV status and peak shedding intensity, vector term, with each element of the vector representing the regression coefficient of a specific HIV infection status.

$\boldsymbol{x}_{i}^{\mathbf{HIV}}$– individual's HIV status categories for infection episode i, vector term, with each element of the vector representing a specific HIV status.

$\boldsymbol{b}_{\mathbf{Y}}^{\mathbf{D}}$ – random effects on shedding duration by calendar year, vector term, with each element of the vector representing the random effect of a specific calendar year.

$\boldsymbol{b}_{\mathbf{Y}}^{\mathbf{P}}$ – random effects on peak shedding intensity by calendar year, vector term, with each element of the vector representing the random effect of a specific calendar year.

$\boldsymbol{x}_{i}^{\mathbf{Y}}$– calendar year when infection episode i occurred, vector term, with each element of the vector representing a specific calendar.

Of the 623 infection episodes included in the viral shedding model, 50 samples tested positive for two influenza subtypes within the same specimen, corresponding to 25 co-infection events, and 77 reinfections were identified across study years. Most co-infections involved the two type A subtypes, which co-circulated during the first year of the study. Reinfections were most frequently observed between influenza A and influenza B viruses, typically when individuals were infected with influenza A early in the season and influenza B later in the same season. These co-

infections and reinfections were retained in the shedding model to contribute to the population-level estimates for each subtype.

However, for the regression analyses assessing the association with pre-season HAI titer, co-infections and reinfections involving the same influenza type within a single season were excluded to avoid confounding due to immune interactions. Among the 77 reinfections, 21 involved the same subtype or type, resulting in the exclusion of 71 episodes in total (co-infections and reinfections combined). An additional 53 episodes were excluded because pre-season HAI titer measurements were unavailable due to missed samples, late enrollment, or sampling errors. The final dataset included 499 infection episodes, comprising 93 A(H1N1)pdm09, 149 A(H3N2), 66 B/Yamagata, and 191 B/Victoria infections.

Using the filtered dataset described above, we fitted linear multivariable or mixed-effect multivariable regression models to each of the 100 realizations of the sampled viral shedding trajectories using R (version 4.3.1, RStudio 2023.6.2.561) package lme4 (version 1.1.34). Parameter estimates across realizations were subsequently pooled with the mice R package (version 3.17.0), thereby propagating uncertainty from the viral shedding kinetics into the final regression estimates. The pooled estimates are summarized in Fig 3 and Supplementary Fig 7.

Supplementary Fig. 2. A sample of the estimated viral shedding kinetic fits from the shedding model.

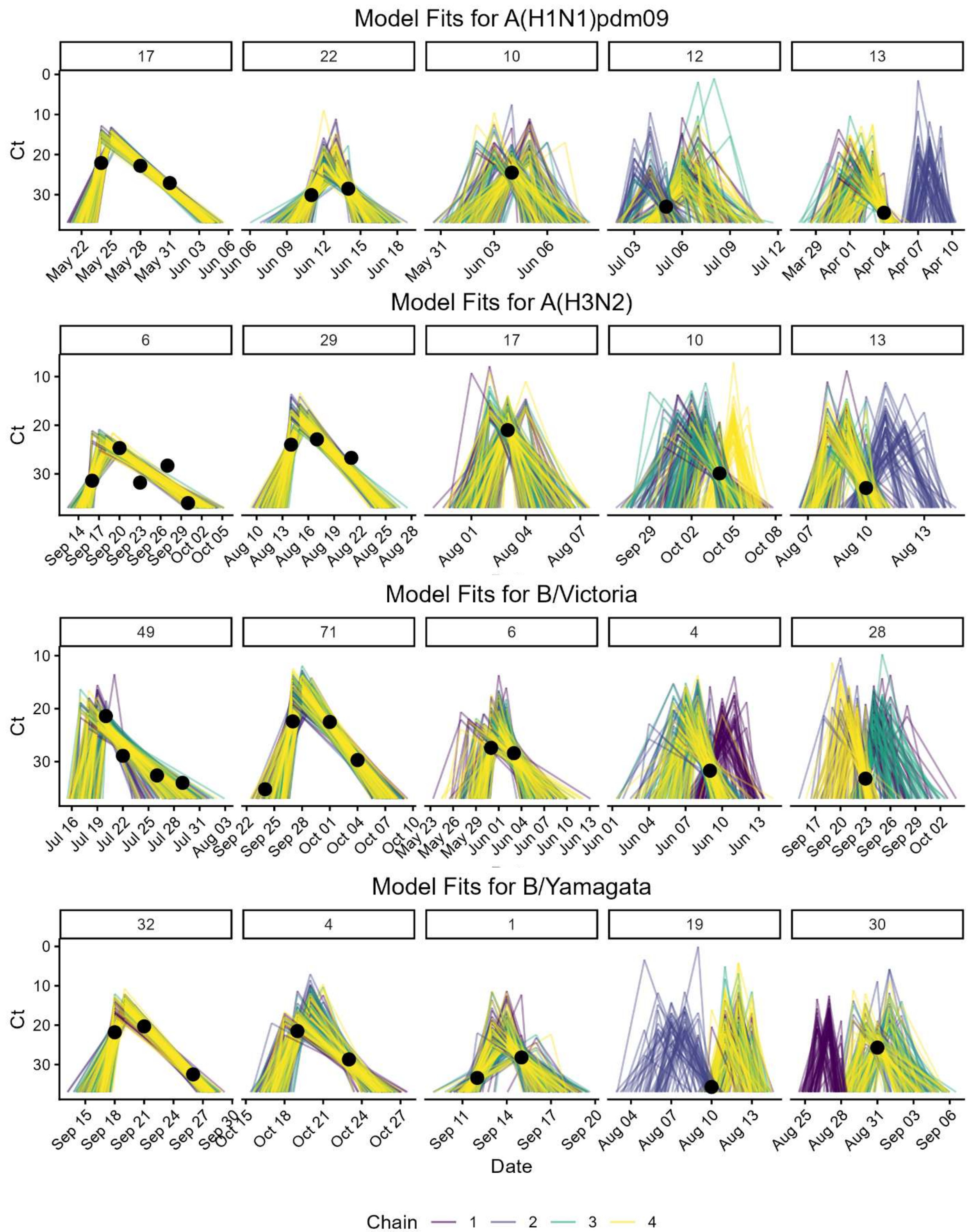

Shown here are five infections for each subtype/lineage. Columns 1 and 2 present two strong fits (R-hat = 1), column 3 presents a one weak fit (1 < R-hat <1.1), and columns 4 and 5 present two poor fits (R-hat >1.1) . The dots are the raw Ct observations that informed the model. The lines depict the first 50 iterations of trace estimates for a given chain of the model as indicated by the line color.

Supplementary Fig. 3. A sample of the model trace plots for estimation of the peak timing of viral shedding (tp).

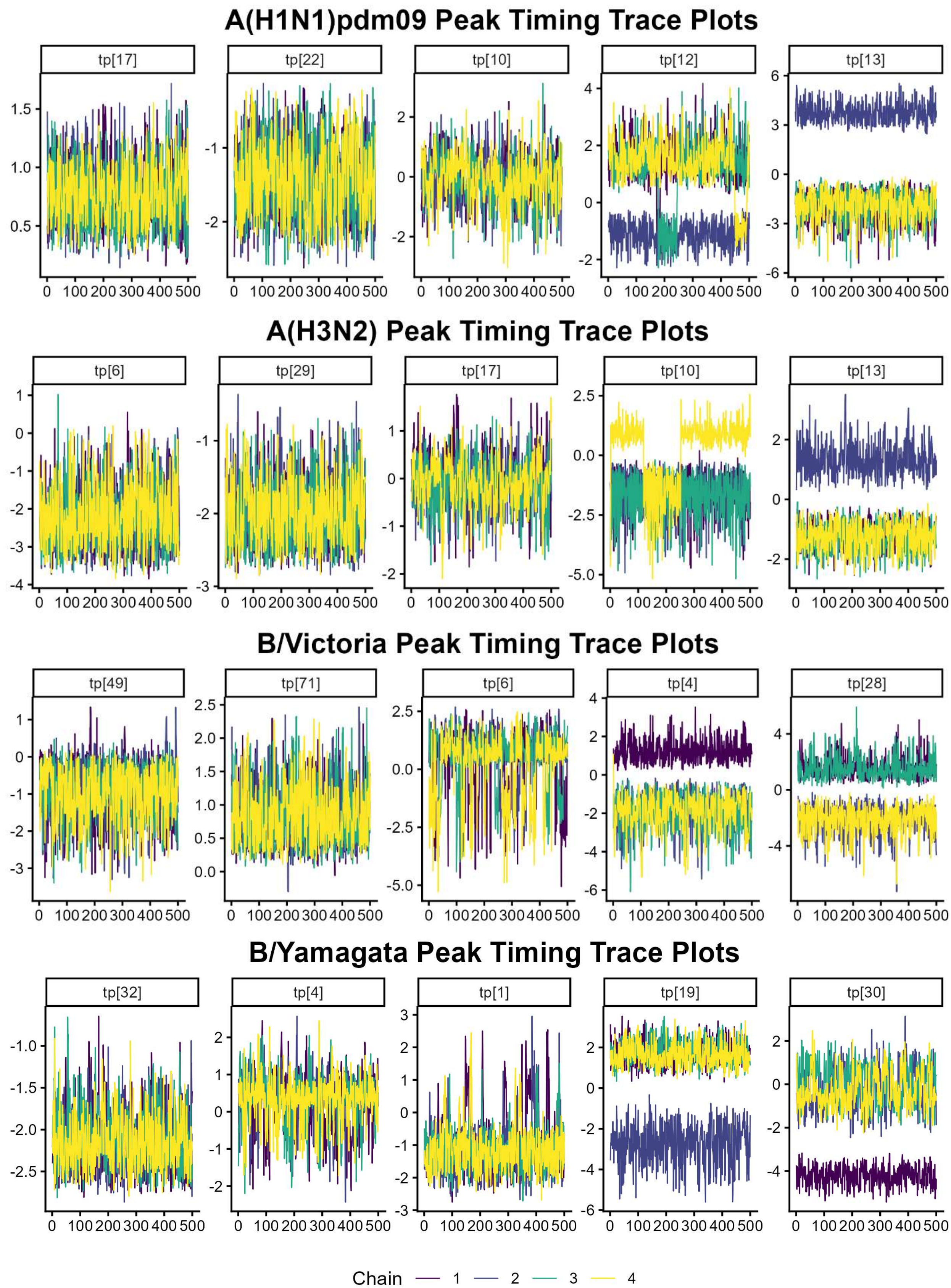

Shown here are five infections for each subtype/lineage. The colored lines represent the estimates of the t_p parameter from the four different chains, as indicated by the color of the line, across iterations 0-500. Columns 1 and 2 present two strong fits (R-hat = 1), column 3 presents a one weak fit (1 < R-hat <1.1), and columns 4 and 5 present two poor fits (R-hat >1.1).

2. Characterize risk factors associated with influenza household transmission

To characterize individual- and household-level risk factors associated with influenza transmission within households, we developed a regression-based transmission model that quantifies the probability of infection acquisition over time. This model links the risk of infection for each household member to both individual characteristics (e.g., age, pre-season immunity, and HIV status) and the household-level force of infection (FOI) derived from the viral shedding model, as well as the community-level risk of exposure.

Observations were created for each day in the influenza season, with a binary dependent variable indicating infection status. The estimated day of infection acquisition was derived from the shedding model, using the estimated duration of shedding and the day of peak infection: we subtracted the estimated proliferation duration from the peak day to obtain the first day of shedding, and, following prior literature, assumed infection acquisition occurred one day before shedding onset [6].

To improve the robustness of the household transmission model, the household and community forces of infection (FOIs) were informed by the characterized viral shedding trajectories of each influenza episode. The shedding model provided higher fidelity estimates of shedding onset compared to raw sampling data. Based on findings from human challenge studies[9,10], we assumed a one-day delay between the time of infection and the onset of viral RNA shedding. The binary dependent variable therefore reflected this estimated day of infection acquisition.

The household force of infection (FOI), defined as the sum of daily viral shedding intensities from infectious household members, was included as a time-varying covariate in the regression model. After accounting for household FOI, we further examined the effect of household size on infection acquisition to adjust for potential reductions in per-person contact intensity in larger households. This so-called dilution effect, where an increase in household size is associated with a lower person-to-person transmission risk, has been reported in previous household transmission studies [10,11]. Including household size thus helps to contextualize household FOI in terms of exposure risk per contact, independent of household size.

The community FOI term was represented by the community-level prevalence of infection within the cohort at time t. This time-varying covariate was smoothed using a four-day moving average, corresponding to the maximum interval between consecutive sample collections. We used the cohort household data because households were randomly selected from the site populations, making cohort infection prevalence broadly representative of the community. Supplementary Fig. 10 shows time series of the smoothed community FOI, with separate figures for school-aged children (5–18) and other ages, with the school calendar indicated to provide context for outbreak timing. While school sessions could influence contact patterns, the community FOI shows no distinct timing differences, supporting its use as a reliable measure of population-level prevalence for the model.

Additional covariates included pre-season HAI titer (categorized by the 1:40 threshold), age, sex, HIV status, and household size (modeled as a continuous variable). The regression models were fitted separately for each influenza subtype to capture subtype-specific transmission dynamics.

Within the transmission model, we also excluded entire households in which one or more individuals had PCR-confirmed infection episodes but lacked estimable viral shedding trajectories, as the corresponding household FOI terms would otherwise omit a known source of infection exposure. The transmission model was implemented only for the season(s) in which a given influenza subtype was dominant; infections that occurred outside those dominant seasons were not included in the analysis. As in the shedding model, additional exclusions were made for individuals with missing pre-season HAI titer data.

The final dataset comprised 87 infection episodes, 923 individuals, and 206 households for A(H1N1)pdm09; 140 infection episodes, 500 individuals, and 105 households for A(H3N2); 165 infection episodes, 866 individuals, and 190 households for B/Victoria; and 64 infection episodes, 507 individuals, and 64 households for B/Yamagata.

To account for uncertainties in the viral shedding kinetics estimated in the previous section, we generated 100 realizations of the viral shedding trajectories by repeatedly resampling from the posterior distributions of the shedding model. For each of these 100 realizations and a given influenza subtype/lineage, a transmission model regression was fitted according to the following equation, which estimates the risk of infection acquisition for individual i at calendar time t. This regression framework is equivalent to a discrete-time survival analysis of infection outcomes but

allows the inclusion of time-varying covariates, such as community and household forces of infection (FOIs), without requiring the proportional hazards assumption [12–14]. The terms of the regressions are defined as follows:

$$y_i(t) = \text{logit}\left(\beta_{\mathrm{C}} \cdot x^{\mathrm{C}}(t) + \beta_{\mathrm{V}} \cdot \sum_{j\neq i}^{j\in h}\left(\Delta \mathrm{Ct}_j^{\mathrm{L}}(t)\right) + \boldsymbol{\beta}_{\mathbf{T}} \cdot \boldsymbol{x}_i^{\mathbf{T}} + \boldsymbol{\beta}_{\mathbf{A}} \cdot \boldsymbol{x}_i^{\mathbf{A}} + \boldsymbol{\beta}_{\mathbf{S}} \cdot \boldsymbol{x}_i^{\mathbf{S}} + \boldsymbol{\beta}_{\mathbf{HIV}} \cdot \boldsymbol{x}_i^{\mathbf{HIV}} + \beta_{\mathrm{HS}} \cdot x_i^{\mathrm{HS}} + \alpha\right)$$

The terms are defined as follows:

β_{C} – log of the hazard-ratio of infection associated with the community force of infection (FOI), scalar term.

x_{C} – Community force of infection (FOI), represented based on the community-level prevalence of influenza infections at the study site at time t, scalar term.

β_{V} – log of the hazard-ratio of infection associated with household FOI, scalar term.

$\sum_{j\neq i}^{j\in h}\left(\Delta \mathrm{Ct}_j^{\mathrm{L}}(t)\right)$ – household FOI at time t as the sum of the viral load of household members apart from the individual i of interest, scalar term.

$\boldsymbol{\beta}_{\mathbf{T}}$ – log of the hazard-ratio of infection associated with the pre-season HAI titer category (<1:40 or $\geq$1:40), vector term.

$\boldsymbol{x}_i^{\mathbf{T}}$ – pre-season HAI titer category for individual i, vector term.

$\boldsymbol{\beta}_{\mathbf{A}}$ – log of the hazard-ratio of infection associated with age group, vector term.

$\boldsymbol{x}_i^{\mathbf{A}}$– age group for individual i, vector term.

$\boldsymbol{\beta}_{\mathbf{S}}$ – log of the hazard-ratio of infection associated with sex, vector term.

$\boldsymbol{x}_i^{\mathbf{S}}$ – sex of individual i, vector term.

$\boldsymbol{\beta}_{\mathbf{HIV}}$ – log of the hazard-ratio of infection associated with HIV status, vector term.

$\boldsymbol{x}_i^{\mathbf{HIV}}$ – HIV status of individual i, vector term.

β_{HS} – log of the hazard-ratio of infection associated with household size, scalar term.

x_i^{HS} – household size of individual i, scalar term.

α – Poisson regression intercept, scalar term.

We fitted Poisson regression models to each of the 100 realizations of the sampled viral shedding trajectories using the glm function in base R (version 4.3.1, RStudio 2023.6.2.561). Parameter estimates across realizations were subsequently pooled with the mice R package

(version 3.17.0), thereby propagating uncertainty from the viral shedding kinetics into the final regression estimates. The pooled estimates are summarized in Figures 5 and Supplementary Fig 8.

3. Spline-Based Visualization of Age-Dependent Trends

The age-dependent trend lines of HAI titers in Fig 2 and Supplementary Fig 9 were generated in R (version 4.3.1, RStudio 2023.6.2.561) using the base R package splines to create spline-based fits to capture non-linear trends in the data. These curves were constructed with B-splines, with knot placement at ages 5, 12, 18, and 40 to describe age-dependent patterns. We used a cubic spline (degree = 3) because cubic functions are the standard choice in spline modeling; they ensure smooth transitions (continuous first and second derivatives) between segments while maintaining interpretability. The age-specific standard deviation, shown as the black line, was calculated at each age and then smoothed using the same spline smoothing method, with identical knot placement and spline degree.

Supplementary Fig. 4. Mosaic plot of nasopharyngeal swabs collected in 2016.

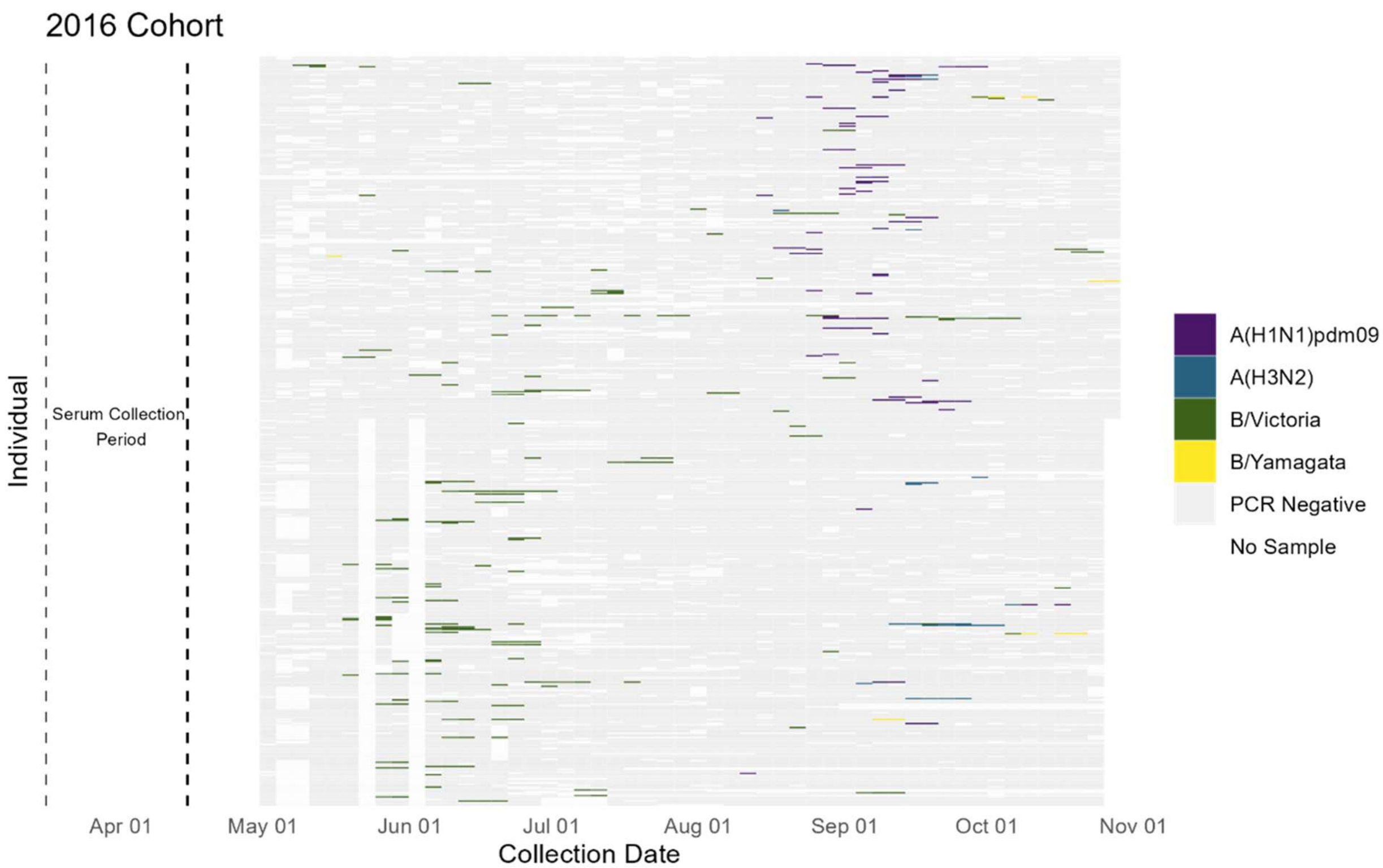

Collection of nasopharyngeal swabs took place from May 5th through October 29th. The serum samples from which the HAI titers used in our analysis were collected between March 16th and April 15th. The color of the tile indicates the result of the swab, including the resulting subtype or lineage in the case that the swab was positive as defined in the figure key. Results are depicted for all 542 individuals followed within this study year, including those that were excluded within the viral shedding and transmission models.

Supplementary Fig. 5. Mosaic plot of nasopharyngeal swabs collected in 2017.

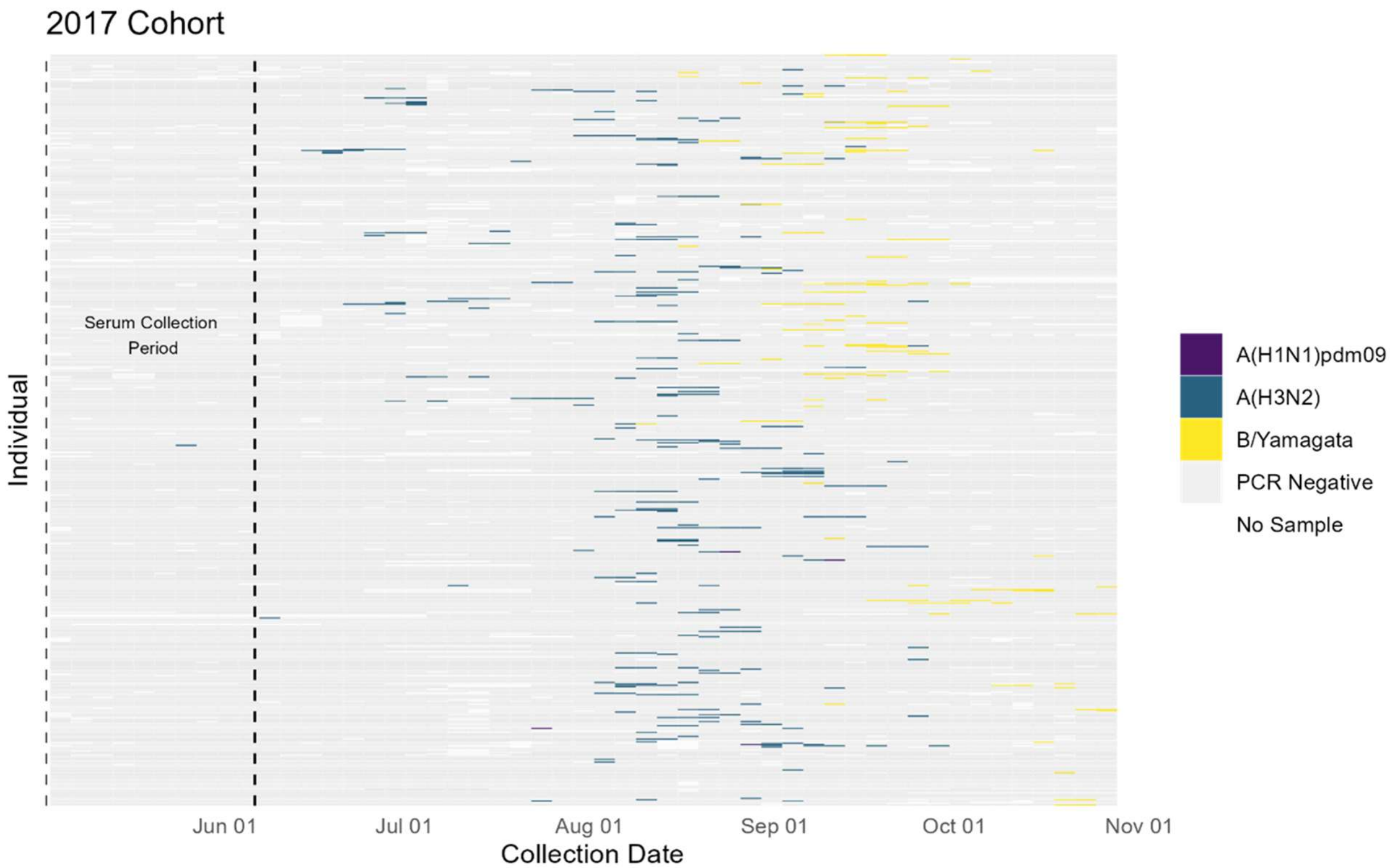

Collection of nasopharyngeal swabs took place from January 16th through October 28th. The serum samples from which the HAI titers used in our analysis were collected between May 2nd and June 6th. The color of the tile indicates the result of the swab, including the resulting subtype or lineage in the case that the swab was positive as defined in the figure key. Results are depicted for all 558 individuals followed within this study year, including those that were excluded within the viral shedding and transmission models.

Supplementary Fig. 6. Mosaic plot of nasopharyngeal swabs collected in 2018.

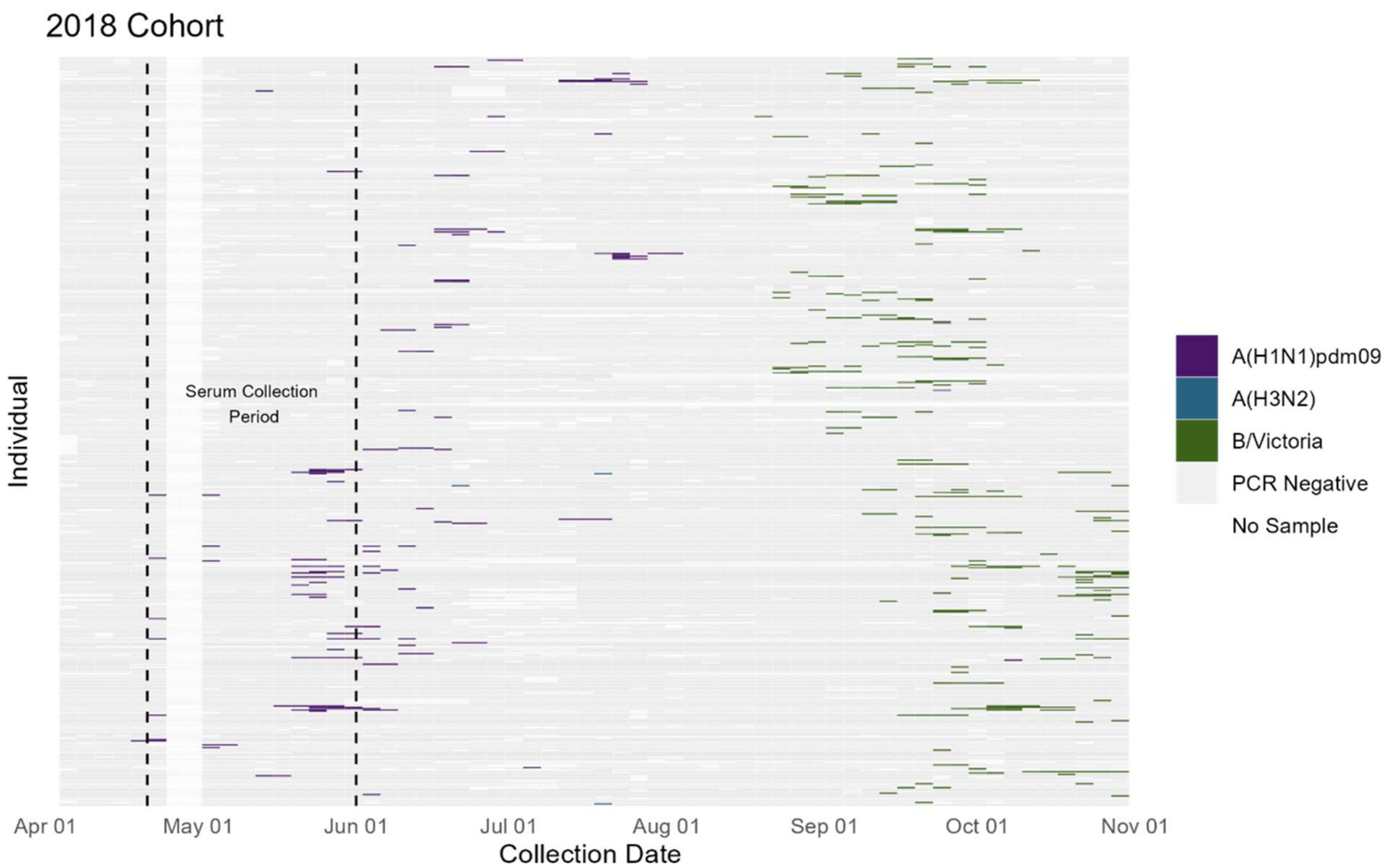

Collection of nasopharyngeal swabs took place from January 15th through October 31st. The serum samples from which the HAI titers used in our analysis were collected between April 21st and June 1th; for individuals that had an infection during this period, a regression was used to estimate their titer for this period based on a sample from November – December of the previous year and the typical titer waning demonstrated in the cohort. The color of the tile indicates the result of the swab, including the resulting subtype or lineage in the case that the swab was positive as defined in the figure key. Results are depicted for all 558 individuals followed within this study year, including those that were excluded within the viral shedding and transmission models.

Supplementary Fig. 7. Predictors of influenza virus shedding characteristics by subtype/lineage based on multivariable linear regression with HAI titer on a continuous log base 4 scale.

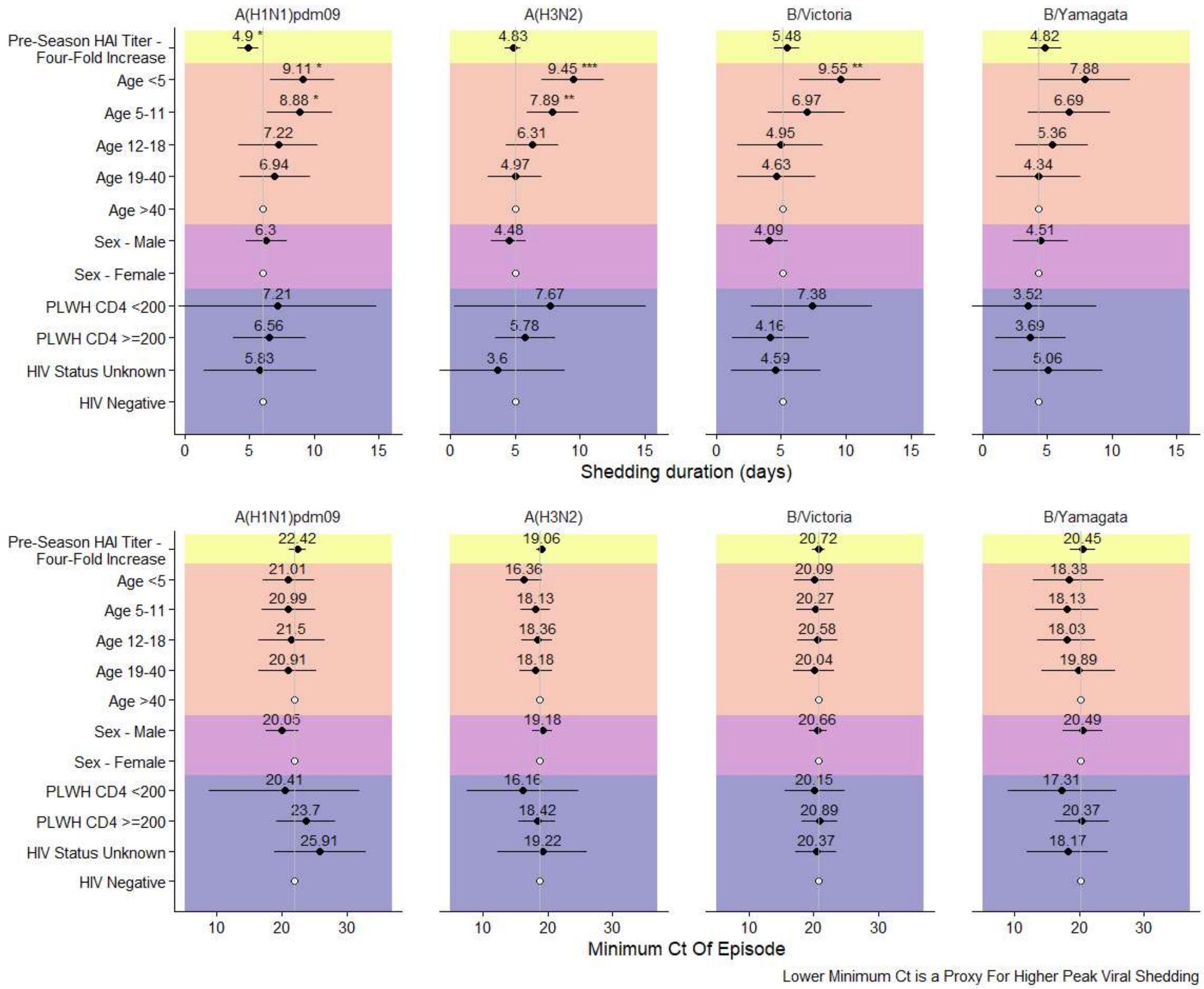

The top panel regresses the total shedding duration and the bottom panel the peak viral load, proxied by the minimum Ct of the infection episode. For the HIV status covariate, PLWH stands for persons living with HIV further defined by demonstrating a CD4 T cell count below or equal to and above 200. Associations were estimated using multivariable mixed-effects linear regression models fitted separately to 100 posterior-resampled viral shedding trajectories, with parameter estimates pooled across realization using Rubin's rules, modeled for independent infection episodes as biological replicates. These analyses included 133 infection episodes for A(H1N1)pdm09, 195 for A(H3N2), 220 for B/Victoria, and 75 for B/Yamagata. Statistical inference was based on two-sided Wald tests with Satterthwaite's approximation for degrees of freedom. No

*adjustment was made for multiple comparisons. For shedding duration, estimates are given in units of days with lines representing the 95% CI. The central line indicates the mean duration for a given subtype/lineage. The reference groups are designated by a white dot. For the shedding duration the reference estimates for each subtype are as follows: A(H1N1)pdm09 6.04 days, A(H3N2) 5.03 days, B/Victoria 5.10 days, B/Yamagata 4.35 days. For the minimum Ct of episode the reference estimates are: A(H1N1)pdm09 22.01 Ct, A(H3N3) 18.80 Ct, B/Victoria 20.71 Ct, B/Yamagata 20.23 Ct. Asterisks denote p-value: '***': <0.001 '**': <0.01 '*': <0.05. Exact p-values for results indicated as significant are as follows: for A(H1N1)pdm09: Pre-Season HAI Four-Fold Increase p = 0.0104, Age < 5 p = 0.0176, Age 5-11 0.0299; for A(H3N2): Age <5 p = 0.0005, Age 5-11 p = 0.0054; for B/Victoria: Age < 5 p = 0.0065.*

Supplementary Fig. 8. Factors influencing the risk of influenza virus infection acquisition by subtype/lineage based on multivariable logistic regression with HAI titer on a continuous log base 4 scale.

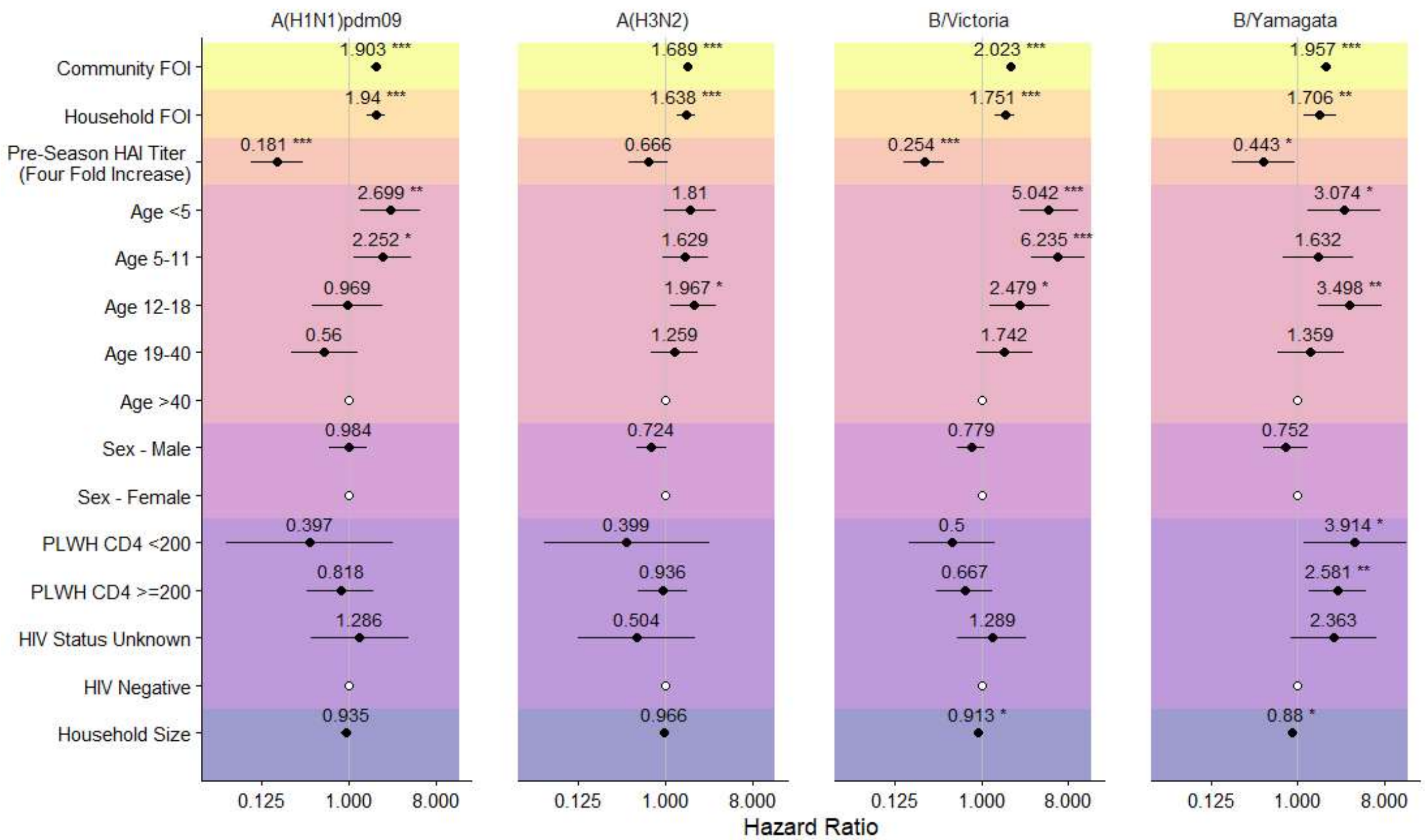

Variables include the community force of infection (FOI), household FOI, pre-season HAI titer, age, sex, HIV status, and household size. Community FOI is indicated with respect to a 0.01 increase in absolute community prevalence (defined as the total number of infection episodes within the cohort at a given time / cohort population). Household FOI is indicated with respect to a step by 10 Ct units within the household. For the HIV status covariate, PLWH stands for persons living with HIV further defined by demonstrating a CD4 T cell count below or equal and above 200. Associations were estimated using multivariable Poisson regression models fitted separately to 100 posterior-resampled viral shedding trajectories, with estimates pooled using Rubin's rules to propagate uncertainty from the shedding model on the timing of infection and time-varying household FOI into the final regression estimates. Statistical inference was based on two-sided Wald tests. Analyses treated individuals as biological estimates and sample sizes included 923 individuals for A(H1N1)pdm09, 500 individuals for A(H3N2), 866 individuals for B/Victoria, and 507 individuals for B/Yamagata. No adjustment was made for multiple comparisons. Black dots represent point estimates of hazard ratios interpreted on the log link scale (Poisson regression is equivalent to a Cox model in this context), with the black horizontal error bars indicating 95% CI

*intervals; white dots indicate reference categories. The reference groups for categorical variables are designated by a white dot. Asterisks denote p-value '***': <0.001 '**': <0.01 '*': <0.05. Exact p-values for results indicated as significant are as follows: for A(H1N1)pdm09: Community FOI* $p = 5 \times 10^{-25}$, *Household FOI* $p = 3 \times 10^{-9}$, *Pre-Season HAI Titer Four Fold Increase* $p = 5 \times 10^{-8}$, *Age < 5* $p = 0.0058$, *Age 5-11* $p = 0.0220$*; for A(H3N2): Community FOI* $p = 5 \times 10^{-37}$, *Household FOI* $p = 6 \times 10^{-6}$, *Age 12-18* $p = 0.0147$*; for B/Victoria: Community FOI* $p = 2 \times 10^{-47}$, *Household FOI* $p = 7 \times 10^{-7}$, *Pre-Season HAI Titer Four Fold Increase* $p = 5 \times 10^{-8}$, *Age < 5* $p = 4 \times 10^{-6}$, *Age 5-11* $p = 2 \times 10^{-8}$, *Age 12-18* $p = 0.0124$, *Household Size* $p = 0.0377$*; for B/Yamagata: Community FOI* $p = 4 \times 10^{-29}$, *Household FOI* $p = 0.0084$, *Pre-Season HAI Titer Four Fold Increase* $p = 0.0317$, *Age < 5* $p = 0.0117$, *Age 12-18* $p = 0.0011$, *PLWH CD4 < 200* $p = 0.0298$, *PLWH CD4 >= 200* $p = 0.0066$, *Household Size* $p = 0.0156$.

Supplementary Fig. 9. Distribution of the pre-season HAI titers separated by observed infection in season.

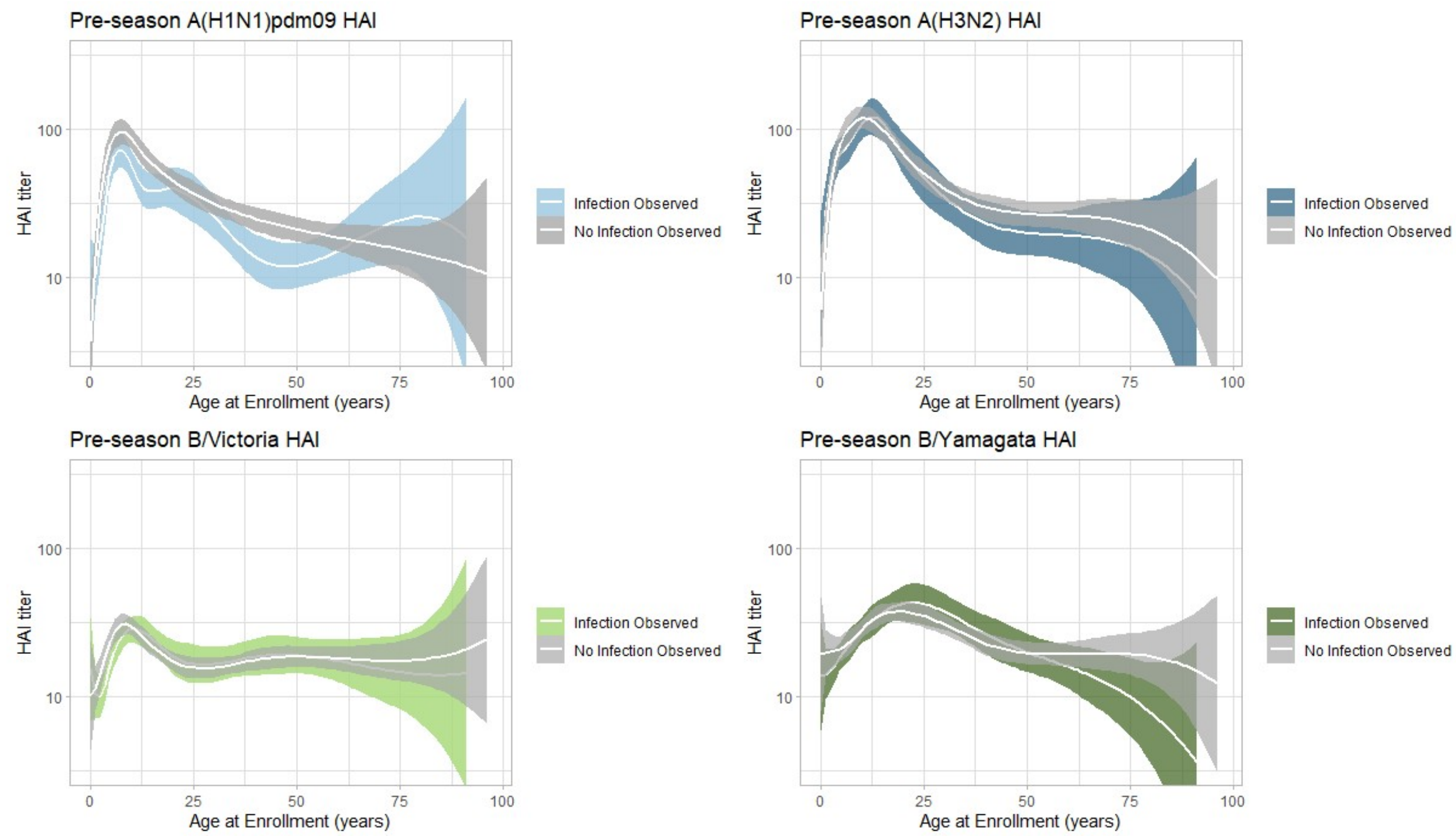

The titer is given on a log base 10 scale and distributed by the age of the participant at enrollment, separated by subtype as indicated. The two lines indicate those who had an observed infection (colored shading) and those who did not have an observed infection (grey shading). The titers were tested with an assay against the recently circulating subtype. The white lines are a fitted spline linear spline fit with B-spline knots at ages 5, 12, 18, and 40 to describe age-dependent patterns, with the shaded region representing the 95% confidence interval.

Supplementary Fig. 10. Temporal trends in infection prevalence informing community FOI

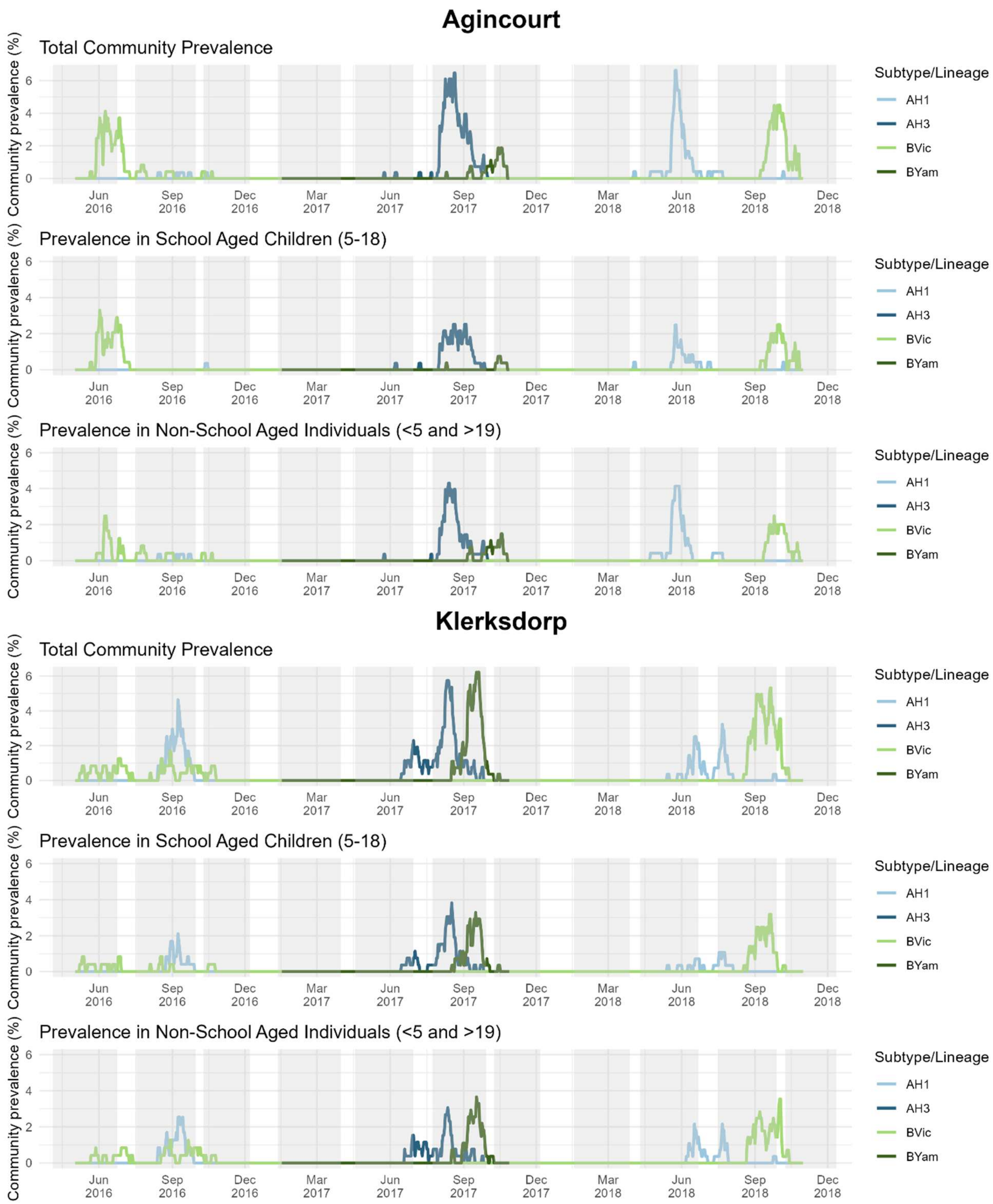

Each set of three panels represents the two cohort sites, Agincourt and Klerksdorp. The top panels of the sets show total community prevalence, calculated as the number of infections on a given day divided by the total cohort population size and smoothed over a 4-day window to account for

potential missing samples. The middle panels show prevalence restricted to school-aged individuals, and the bottom panels show prevalence among non-school-aged individuals. The colored lines correlate to the prevalence for each of the four subtypes/lineages. Community FOI, and thus the prevalence here, was only calculated for the years used in the transmission model for each subtype/lineage (2016 and 2018 for A(H1N1)pdm09 and B/Victoria, 2017 for A(H3N2) and B/Yamagata). The grey shaded regions in each figure indicate when school was in session.

Supplementary Fig. 11. Household cumulative infection risk by household size and influenza virus subtype/lineage.

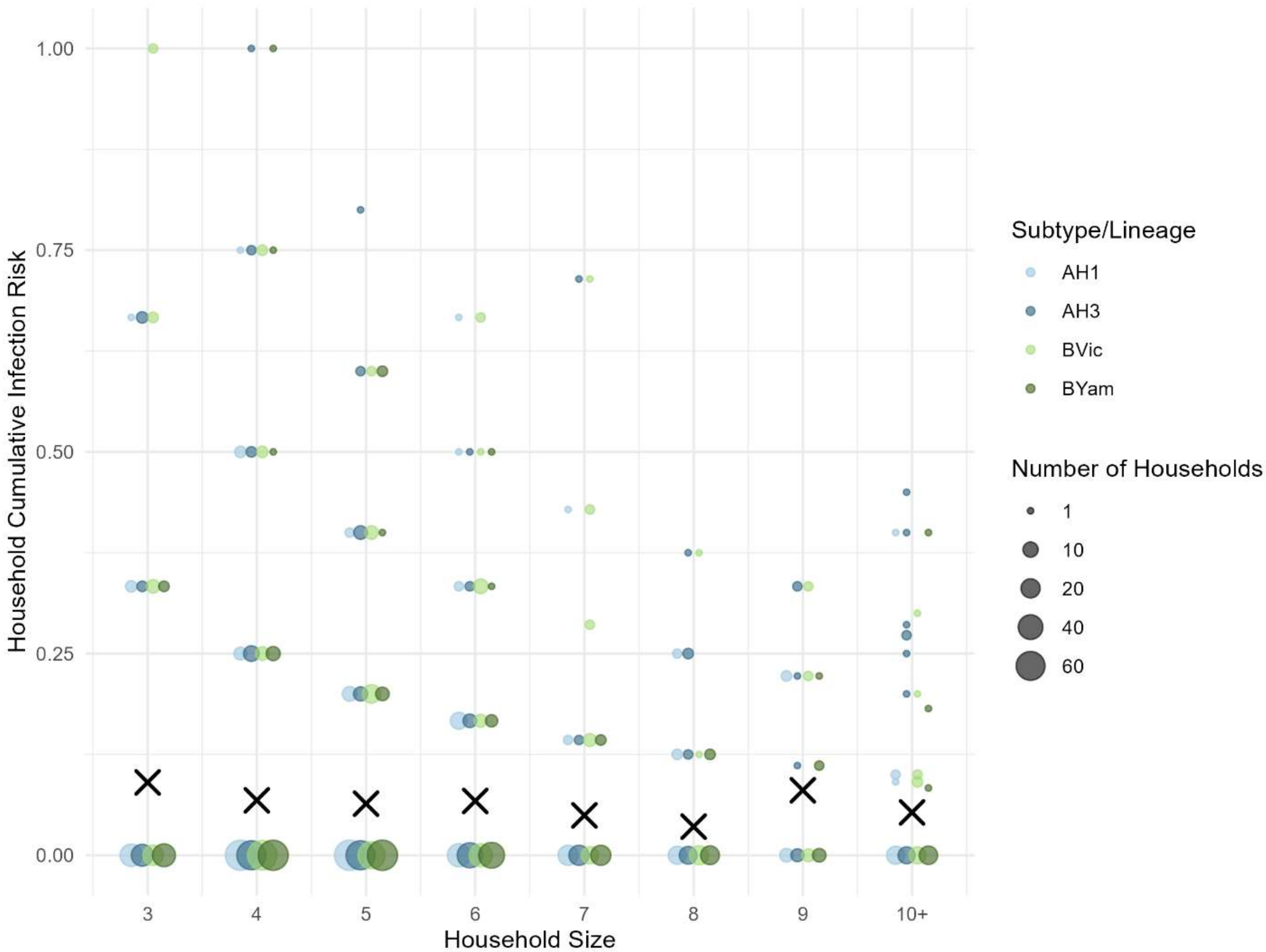

Each point represents the observed cumulative infection risk for a given household size and influenza virus subtype or lineage, with the size of the dot representing the number of households. The cumulative infection risk was calculated as the proportion of individuals experiencing an infection, based on the raw observational data, within each household during the observed influenza season. The black "X" marks the mean cumulative infection risk for the household size. All households of size 10 or larger were grouped into the "10+" category.

Supplementary Fig. 12. Distribution of the pre-season serum influenza antibody titers across age.

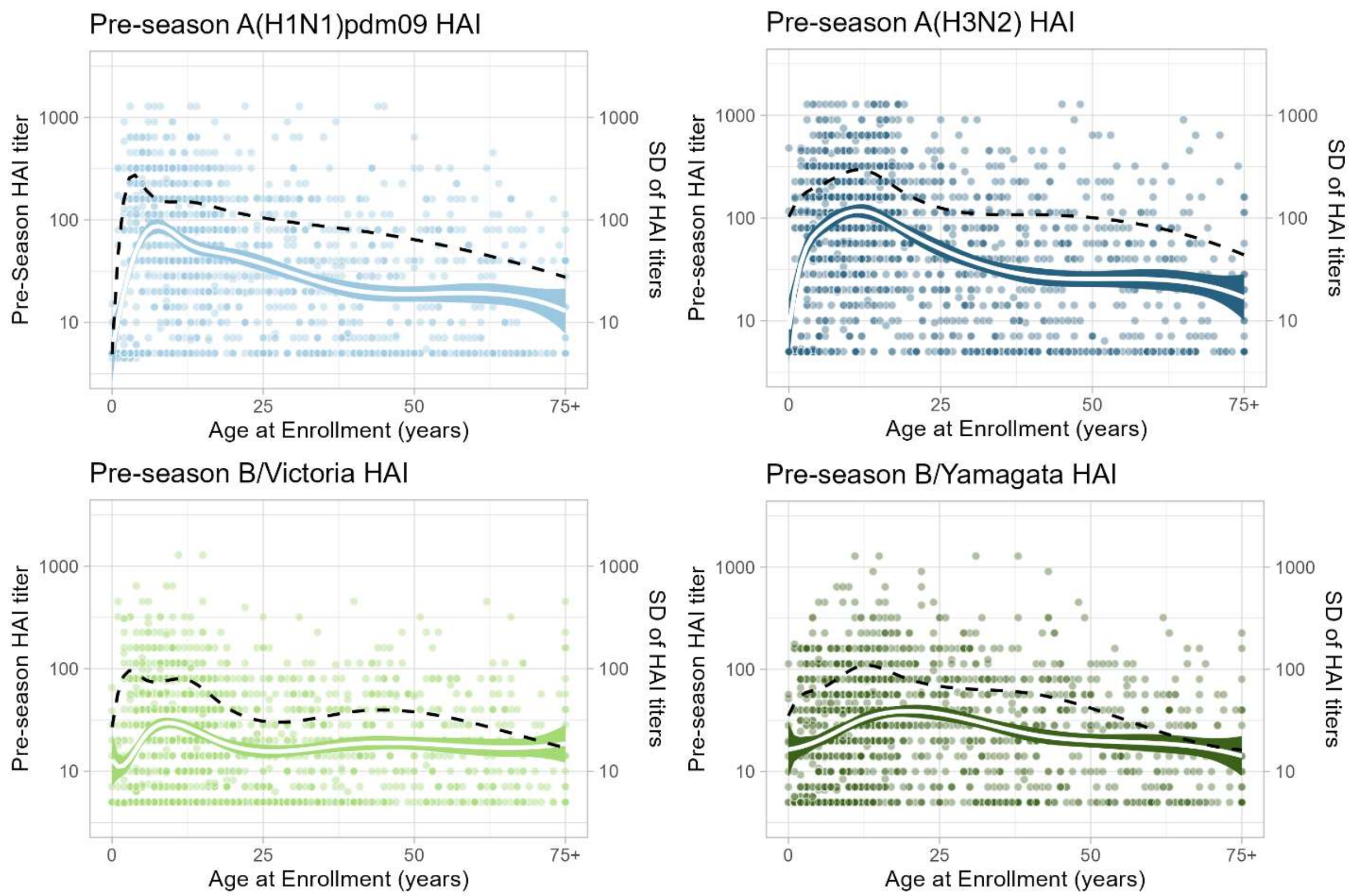

Titers shown as a function of age of the participant at enrollment, separated by subtype/lineage. All participants over 75 are grouped together. The titers were generated using hemagglutination inhibition assay (HAI) against representative circulating viruses during the study period. The white solid line is a fitted linear spline fit with B-spline knots at ages 5, 12, 18, and 40 to describe age-dependent patterns with colored shaded regions representing the 95% confidence intervals. The black dotted line reflects the age-specific standard deviation of the titers smoothed with the same spline method and knot placement transformed on the log 10 scale.

Supplementary Table 1. Coefficients of variation for the estimated proliferation and clearance shedding durations for each subtype/lineage.

Subtype/Lineage	Proliferation Coefficient of Variation	Clearance Coefficient of Variation
A(H1N1)pdm09	28.0	82.6
A(H3N2)	69.3	78.3
B/Victoria	61.9	83.0
B/Yamagata	39.2	68.7

Supplementary Table 2. Model building to determine variables included in the main model regression.

Subtype/ Lineage Model	Community FOI	+ Household FOI	+ Age	+ Categorical (1:40) Pre-season Titer	+ Sex	+ HIV Status	+ Household Size (**Main Model**)	+ Year as fixed effect	+ Year as random effect	Main Model + Study Site	Main Model + BMI	Main Model + Living With Child under 5 Years Old	Main Model + Crowding in Household
A(H1N1)pdm09	1501.3	1466.1	1445.2	1423	1425	1429.7	1430.7	1432.3	Singular Fit	1431.2	1436.2	1432.4	1432.5
A(H3N2)	1962.9	1944.9	1949.5	1946.8	1945.5	1948.6	1948.1			1946.7	1952.7	1950.1	1948.9
B/Victoria	2401.2	2381.9	2319.6	2309.6	2311.2	2315.2	2312.9	2299.8	Singular Fit	2314.4	2319.4	2314.4	2314.8
B/Yamagata	959.82	958.2	961.15	961.23	959.85	954.94	950.68			951.69	956.14	950.98	951.97

Values given represent the AIC for the described model. The highlighted cell represents the regression used for the main model. FOI stands for force of infection. BMI stands for body mass index.

Supplementary Table 3. Sensitivity analysis on the household force of infection (FOI) and community FOI terms included in the main model regression.

Subtype/Lineage Model	Main Model	Main Model Without Community FOI	Null Community Model (Binary Community FOI Term Representing Prevalent/Absent in Study Site)	Main Model Without Household FOI	Null Household Model (Binary Household FOI Term Representing Prevalent/Absent in Household)
A(H1N1)pdm09	1430.7	1439.1	1338.1	1473.2	1397.4
A(H3N2)	1948.1	2129.2	1901.6	1968.3	1945.4
B/Victoria	2306.3	2532.2	2286.7	2338	2285.4
B/Yamagata	950.68	1082.9	922.03	952.03	946.97

Values given represent the AIC of the given model. For the community null model, even though the model fits better, the estimates become very high (ex. 42 odds ratio or a 4000% increase in risk of H3 infection acquisition, which suggests a vast oversimplification of the parameter). Similarly, though the null household model fits slightly better for the null household model, in the A(H1N1)pdm09 regression, the community FOI is no longer significant meaning that there would be no significant risk of infection acquisition from community exposure suggesting again an oversimplification of the parameter that confounds the results.

Supplementary Methods References

1. Cohen, C. *et al.* Asymptomatic transmission and high community burden of seasonal influenza in an urban and a rural community in South Africa, 2017–18 (PHIRST): a population cohort study. *Lancet Glob. Health* **9**, e863–e874 (2021).
2. Cohen, C. *et al.* Cohort profile: A Prospective Household cohort study of Influenza, Respiratory syncytial virus and other respiratory pathogens community burden and Transmission dynamics in South Africa, 2016–2018. *Influenza Other Respir. Viruses* **15**, 789–803 (2021).
3. Jernigan, D. B. *et al.* Detecting 2009 pandemic influenza A (H1N1) virus infection: availability of diagnostic testing led to rapid pandemic response. *Clin. Infect. Dis. Off. Publ. Infect. Dis. Soc. Am.* **52 Suppl 1**, S36-43 (2011).
4. Kissler, S. M. *et al.* Viral dynamics of acute SARS-CoV-2 infection and applications to diagnostic and public health strategies. *PLOS Biol.* **19**, e3001333 (2021).
5. Sun, K. *et al.* SARS-CoV-2 transmission, persistence of immunity, and estimates of Omicron's impact in South African population cohorts. *Sci. Transl. Med.* **14**, eabo7081 (2022).
6. Carrat, F. *et al.* Time lines of infection and disease in human influenza: a review of volunteer challenge studies. *Am. J. Epidemiol.* **167**, 775–785 (2008).
7. Lau, L. L. H. *et al.* Viral shedding and clinical illness in naturally acquired influenza virus infections. *J. Infect. Dis.* **201**, 1509–1516 (2010).
8. Ip, D. K. M. *et al.* The Dynamic Relationship Between Clinical Symptomatology and Viral Shedding in Naturally Acquired Seasonal and Pandemic Influenza Virus Infections. *Clin. Infect. Dis. Off. Publ. Infect. Dis. Soc. Am.* **62**, 431–437 (2016).
9. Li, C.-C. *et al.* Correlation of pandemic (H1N1) 2009 viral load with disease severity and prolonged viral shedding in children. *Emerg. Infect. Dis.* **16**, 1265–1272 (2010).
10. Gordon, A. *et al.* Influenza Transmission Dynamics in Urban Households, Managua, Nicaragua, 2012–2014. *Emerg. Infect. Dis.* **24**, (2018).
11. Cauchemez, S., Carrat, F., Viboud, C., Valleron, A. J. & Boëlle, P. Y. A Bayesian MCMC approach to study transmission of influenza: application to household longitudinal data. *Stat. Med.* **23**, 3469–3487 (2004).
12. Holford, T. R. The Analysis of Rates and of Survivorship Using Log-Linear Models. *Biometrics* **36**, 299–305 (1980).
13. Laird, N. & Olivier, D. Covariance Analysis of Censored Survival Data Using Log-Linear Analysis Techniques. *J. Am. Stat. Assoc.* **76**, 231–240 (1981).
14. Sun, K. *et al.* SARS-CoV-2 transmission, persistence of immunity, and estimates of Omicron's impact in South African population cohorts. *Sci. Transl. Med.* **14**, eabo7081 (2022).
