# Supplementary material for: The roles of pre-season immunity, age, viral shedding, and community exposures in shaping influenza household transmission dynamics
Source: Nat Commun. 2026 Jul 30;17:9238. doi: 10.1038/s41467-026-76037-x (PMC13526887; doi:10.1038/s41467-026-76037-x)
Supplement: Supplementary file 2 [file 41467_2026_76037_MOESM2_ESM.pdf]

nature portfolio

Corresponding author(s): Kaiyuan Sun

Last updated by author(s): Oct 29, 2025

Reporting Summary

Nature Portfolio wishes to improve the reproducibility of the work that we publish. This form provides structure for consistency and transparency in reporting. For further information on Nature Portfolio policies, see our Editorial Policies and the Editorial Policy Checklist.

Statistics

For all statistical analyses, confirm that the following items are present in the figure legend, table legend, main text, or Methods section.

n/a	Confirmed	
☐	☒	The exact sample size (*n*) for each experimental group/condition, given as a discrete number and unit of measurement
☐	☒	A statement on whether measurements were taken from distinct samples or whether the same sample was measured repeatedly
☐	☒	The statistical test(s) used AND whether they are one- or two-sided<br>*Only common tests should be described solely by name; describe more complex techniques in the Methods section.*
☐	☒	A description of all covariates tested
☐	☒	A description of any assumptions or corrections, such as tests of normality and adjustment for multiple comparisons
☐	☒	A full description of the statistical parameters including central tendency (e.g. means) or other basic estimates (e.g. regression coefficient) AND variation (e.g. standard deviation) or associated estimates of uncertainty (e.g. confidence intervals)
☐	☒	For null hypothesis testing, the test statistic (e.g. F, t, r) with confidence intervals, effect sizes, degrees of freedom and P value noted<br>*Give P values as exact values whenever suitable.*
☐	☒	For Bayesian analysis, information on the choice of priors and Markov chain Monte Carlo settings
☐	☒	For hierarchical and complex designs, identification of the appropriate level for tests and full reporting of outcomes
☐	☒	Estimates of effect sizes (e.g. Cohen's *d*, Pearson's *r*), indicating how they were calculated

Our web collection on statistics for biologists contains articles on many of the points above.

Software and code

Policy information about availability of computer code

Data collection	Data were entered during visits on tablet computers with use of the Research Electronic Data Capture application (REDCap).
Data analysis	All the statistical analysis for this study and the figures were performed in R (version 4.3.1, RStudio 2023.6.2.561). The corresponding code to reproduce all figures, as well as executable code using pseudo data to validate the viral shedding kinetics model, viral shedding regression models, and household transmission model, can be found at https://github.com/msauter19/phirst-flu-shed-trans. All R packages required ad versions used are listed in the README file in the GitHub repository and those specific to aspects of the statistical analysis are listed throughout the Supplementary Information. All packages used includes: tidyverse (version 2.0.0), rstan (version 2.32.6), mice (version 3.17.0), zoo (version 1.8.12), tidyr (version 1.3.0), ggplot2 (version 4.0.0), cowplot (version 1.1.3), dplyr (version 1.1.3), lares (version 5.2.2), grid (version 4.3.1), posterior (version 1.4.1), bayesplot (version 1.10.0).

For manuscripts utilizing custom algorithms or software that are central to the research but not yet described in published literature, software must be made available to editors and reviewers. We strongly encourage code deposition in a community repository (e.g. GitHub). See the Nature Portfolio guidelines for submitting code & software for further information.

Data

Policy information about availability of data

All manuscripts must include a data availability statement. This statement should provide the following information, where applicable:

- Accession codes, unique identifiers, or web links for publicly available datasets
- A description of any restrictions on data availability
- For clinical datasets or third party data, please ensure that the statement adheres to our policy

The PHIRST Cohort Data are available under restricted access as the individual-level data cannot be publicly shared due to ethical restrictions and the potential for identifying included individuals. Access to the individual participant data, and a data dictionary defining each field in the dataset, requires provision of protocol and ethics approval for the proposed use. To request individual participant data access, please submit a proposal to CC (cherylc@nicd.ac.za) who will respond within 1 month of request. Upon approval, data can be made available through a data sharing agreement. Source Data are provided with this paper; these data are aggregate, de-identified and/or model-derived estimates sufficient to reproduce all data-driven figures and tables presented in the manuscript. For the underlying models requiring individual-level participant data, pseudo-data, structured in the same format as the clinical data, can be found at https://github.com/msauter19/phirst-flu-shed-trans.

Research involving human participants, their data, or biological material

Policy information about studies with human participants or human data. See also policy information about sex, gender (identity/presentation), and sexual orientation and race, ethnicity and racism.

Reporting on sex and gender	Information on sex were collected based on self-reporting.
Reporting on race, ethnicity, or other socially relevant groupings	NA
Population characteristics	This study included 1518 total individuals split between two study sites, with 748 (49.3%) individuals residing in Agincourt (rural study site) and 770 (49.7%) in Klerksdorp (urban study site). Age of the individuals was grouped for analyses: 176 (11.6%) were under 5 years old, 370 (24.4%) were 5-11 years old, 298 (19.6%) were 12-18 years old, 359 (23.6%) were 19-40 years old, and 310 (20.4%) were over 40 years old. The population was 60.6% female. HIV status was known for 1482 (97.6%) of the population. Of those with known status, 83.9% were HIV Negative, 13.1% were people living with HIV (PLWH) with a CD4 T cell count above or equal to 200, and 3% were PLWH with a CD4 T cell count below 200. Individuals were enrolled as a household. Of the 325 households included in the study, 194 (59.7%) had 3-5 household members, 119 (36.6%) had 6-10 members, and 12 (3.7%) had over 10 members. Population characteristics for the full cohort population, with characteristics described not relevant to our study, are detailed at https://onlinelibrary.wiley.com/doi/10.1111/irv.12881.
Recruitment	See https://onlinelibrary.wiley.com/doi/10.1111/irv.12881 for details
Ethics oversight	The PHIRST (Prospective Household cohort study of Influenza, Respiratory Syncytial virus, and other respiratory pathogens community burden and Transmission dynamics in South Africa) protocol was approved by the University of the Witwatersrand Human Research Ethics Committee (Reference 150808) and the US CDC's Institutional Review Board relied on the local review (#6840). The protocol was registered on http://clinicaltrials.gov on 6 August 2015 (Reference NCT02519803; https://clinicaltrials.gov/ct2/show/NCT02519803). Participants provided individual written consent or assent prior to enrollment and received a grocery store voucher of ZAR25-30 (USD 2–2.5) per visit for their time. (See 45 C.F.R. part 46.114; 21 C.F.R. part 56.114).

Note that full information on the approval of the study protocol must also be provided in the manuscript.

Field-specific reporting

Please select the one below that is the best fit for your research. If you are not sure, read the appropriate sections before making your selection.

[x] Life sciences [ ] Behavioural & social sciences [ ] Ecological, evolutionary & environmental sciences

For a reference copy of the document with all sections, see nature.com/documents/nr-reporting-summary-flat.pdf

Life sciences study design

All studies must disclose on these points even when the disclosure is negative.

Sample size	This study represents analysis of the cohort data that was not planned during the original study design. The protocol for the original study design describes their sample size determination as follows: "The sample size of approximately 1500 individuals over 3 consecutive seasons will allow the estimation of 20% risk of infection and a 10% risk of illness with 95% CI and 5% desired absolute precision in the community" (https://clinicaltrials.gov/study/NCT02519803#study-overview). At the conclusion of the study, the PHIRST cohort had enrolled 1684 individuals for which 1605 completed all follow-up (See https://onlinelibrary.wiley.com/doi/10.1111/irv.12881 for details). Of the individuals that completed follow-up, our analyses required that some individuals be excluded for reasons detailed below, leaving the final sample size of individuals included in this study to be 1518. This sample size is still well within the intended sample size from the original study design determined to be representative of the community.
Data exclusions	In some cases, nasopharyngeal samples tested negative for all subtype-specific primers despite being positive for the broader influenza type A

or B primers. When such samples occurred within the timeframe of an established infection episode for a known subtype, they were retained and assigned the same subtype as the surrounding samples. Samples that remained untyped after this correction were assigned a subtype based on the dominant circulating lineage during that season: B/Victoria in 2016, A(H3N2) and B/Yamagata in 2017, and B/Victoria and A (H1N1)pdm09 in 2018. Non-subtyped samples likely reflected low viral RNA concentrations and excluding them could bias population-level shedding parameter estimates. However, due to the co-circulation of A(H3N2) and A(H1N1)pdm09 in 2016, non-subtyped influenza A samples from that year were excluded. This exclusion affected 14 samples, each representing a distinct infection episode.

We also excluded certain infection episodes from the shedding model estimates due to the proximity of missing samples. This included if there were two missing samples prior to the first positive sample or after the last positive sample of an episode. In other words, that we did not have data for a week on either end of the infection, inhibiting the opportunity to estimate the duration with accuracy. We also excluded infections that were cut off by either the start or the end of the collection period, with a positive occurring within one negative or missing sample from start or end collection date; this was mostly of the B/Victoria wave that occurred relatively late in 2018. This excluded 58 out of 681 infections. For the regression analyses assessing the association with pre-season HAI titer, co-infections and reinfections involving the same influenza type within a single season were excluded to avoid confounding due to immune interactions. Among the 77 reinfections, 21 involved the same subtype or type, resulting in the exclusion of 71 episodes in total (co-infections and reinfections combined). An additional 53 episodes were excluded because pre-season HAI titer measurements were unavailable due to missed samples, late enrollment, or sampling errors. The final dataset included 499 infection episodes, comprising 93 A(H1N1)pdm09, 149 A(H3N2), 66 B/Yamagata, and 191 B/Victoria infections.

Within the transmission model, we also excluded entire households in which one or more individuals had PCR-confirmed infection episodes but lacked estimable viral shedding trajectories, as the corresponding household FOI terms would otherwise omit a known source of infection exposure. The transmission model was implemented only for the season(s) in which a given influenza subtype was dominant; infections that occurred outside those dominant seasons were not included in the analysis. As in the shedding model, additional exclusions were made for individuals with missing pre-season HAI titer data. The final dataset comprised 87 infection episodes, 923 individuals, and 206 households for A(H1N1)pdm09; 140 infection episodes, 500 individuals, and 105 households for A(H3N2); 165 infection episodes, 866 individuals, and 190 households for B/Victoria; and 64 infection episodes, 507 individuals, and 64 households for B/Yamagata.

Replication: NA (This is a prospective household observational study , circulating virus and population immunity have fundamentally changed throughout the study thus it is impossible to replicate the real-world epidemiological situations in a similar population)

Randomization: NA (covariates were controlled by fitting a multi-variable regression model

Blinding: NA (This is a prospective household observational study, no blinding performed)

Reporting for specific materials, systems and methods

We require information from authors about some types of materials, experimental systems and methods used in many studies. Here, indicate whether each material, system or method listed is relevant to your study. If you are not sure if a list item applies to your research, read the appropriate section before selecting a response.

Materials & experimental systems

n/a	Involved in the study
☒	☐ Antibodies
☒	☐ Eukaryotic cell lines
☒	☐ Palaeontology and archaeology
☒	☐ Animals and other organisms
☐	☒ Clinical data
☒	☐ Dual use research of concern
☒	☐ Plants

Methods

n/a	Involved in the study
☒	☐ ChIP-seq
☒	☐ Flow cytometry
☒	☐ MRI-based neuroimaging

Clinical data

Policy information about clinical studies

All manuscripts should comply with the ICMJE guidelines for publication of clinical research and a completed CONSORT checklist must be included with all submissions.

Clinical trial registration: NCT02519803

Study protocol: https://clinicaltrials.gov/study/NCT02519803#study-overview

Data collection: 2.3 Baseline, symptom and health contact data

Data were collected using REDCap (Research Electronic Data Capture)19. Following enrolment, a baseline questionnaire was completed for each household including information on household members, relationships, sleeping arrangements and housing. For each individual, we collected baseline information on demographics, underlying illnesses, vaccinations and occupation. During the twice-weekly follow-up phase, at each visit, for each participant, a questionnaire assessing presence of symptoms, absenteeism and health system contacts was completed and nasopharyngeal (NP) swabs were collected regardless of the presence or absence of symptoms (Table 1). Field workers were trained in the identification of respiratory signs and symptoms at the beginning of each year. Symptoms assessed at twice-weekly visits included fever (self-reported or measured tympanic temperature ≥38°C), cough, difficulty breathing, sore throat, nasal congestion, chest pain, muscle aches, headache, vomiting or diarrhoea. In 2017 and 2018, procedures for collection of symptom data were improved following review of symptom data from 2016. These improvements included simplification of the symptom collection form and monthly training of fieldworkers on signs and symptoms identification and

recording and frequent emphasis of the importance of symptom reporting to participants.

2.4 Laboratory measurements

NP samples were collected using flexible nasopharyngeal nylon flocked swabs (PrimeSwab™, Longhorn Vaccines & Diagnostics, San Antonio, Texas, USA), placed in PrimeStore® Molecular Transport Medium (MTM) (Longhorn Vaccines & Diagnostics, San Antonio, Texas, USA) and transported at 2–8°C to the National Institute for Communicable Diseases (NICD) in Johannesburg, where the samples were tested, aliquoted and stored at 2–8°C before freezing at −70°C. Nucleic acids were extracted from PrimeStore® MTM using the Roche MagNA Pure 96 instrument (Roche, Mannheim, Germany) according to the manufacturer's instructions. NP samples were tested for RSV and influenza A and B viruses by real-time PCR (RT-PCR) using the FTD Flu/RSV detection assay (Fast Track Diagnostics, Luxembourg). Influenza A positive samples were subtyped using the Centers for Disease Control and Prevention (CDC) influenza A (H1/H3/H1pdm09) subtyping kit and influenza B lineage was determined using the CDC B/Yamagata-B/Victoria lineage typing kit (available through International Reagent Resource Program; http://www.internationalreagentresource.org) using SuperScript™ III One-Step RT-PCR System with Platinum™ Taq DNA Polymerase (ThermoFisher, Waltham, Massachusetts, USA)20. RSV A and B subgroups were determined by an in-house RT-PCR.21, 22 Twice-weekly NP samples were tested for S. pneumoniae using an in-house singleplex (lytA) quantitative real-time PCR assay.23 NP and sputum samples were tested for Bordetella spp. (including B. pertussis, B. parapertussis, B. bronchiseptica and B. holmesii) by a combination of a triplex and singleplex real-time PCR assays and results interpreted as previously described.24

Clotted blood samples from serology surveys collected in vacutainer tubes were centrifuged, aliquoted and stored frozen before being sent in batches to NICD on dry ice. Hemagglutination inhibition (HAI) assays using turkey red blood cells were performed to determine serological reactivity titres for serum samples against influenza.25 Virus strains for testing were selected based on the Southern Hemisphere vaccine strains and strains predominantly circulating in South Africa during each year. Cultures from circulating strains representing the following subtypes and lineages were prepared in Madin–Darby Canine Kidney cells: A/Singapore/NFIMH-16-0019/2016-like for influenza A (H3N2), A/Michigan/45/2015-like for influenza A(H1N1)pdm09, B/Brisbane/60/2008-like for influenza B Victoria lineage and B/Phuket/3073/2013-like for influenza B Yamagata lineage. RSV antibody titres were determined using an enzyme-linked immunosorbent assay (ELISA) (EUROIMMUN Anti-RSV IgG; Lübeck, Germany).26 Serology for pertussis antibody was performed using the commercially available anti-Pertussis toxin ELISA (EUROIMMUN, Lübeck, Germany).27

Urine cotinine tests were performed using the IMMULITE® 1000 Nicotine Metabolite Assay Kit (Siemens Medical Solutions Diagnostics, Gly Rhonwy, UK). Sputum samples were collected at baseline from individuals who could expectorate and thereafter from any individual with fever, cough or weight loss for >2 weeks, assessed monthly. Sputum samples were tested for tuberculosis using GeneXpert MTB/RIF (Cepheid, Sunnyvale, CA, USA) and cultured using the Mycobacteria Growth Indicator Tube (MGIT) 960 instrument (Becton Dickinson, East Rutherford, NJ, USA).

Tuberculin skin tests were performed twice-annually 6–10 months apart. Five units of purified tuberculin were injected intradermally on the volar aspect of the forearm. Results were assessed 48–72 h thereafter, for induration and, if present, study staff measured the diameter in millimetres transverse to the axis of the forearm. HIV testing, according to the double rapid test algorithm in place in each province was offered to all participants older than 10 years of age.28 Participants were considered HIV infected if they had one of the following during the follow-up period: two positive rapid HIV tests, evidence of a positive HIV laboratory result or evidence of antiretroviral treatment. Participants were considered HIV uninfected if they had a documented negative HIV test result during the study. A documented HIV negative status for the mother was considered confirmation of HIV negative status for a child aged <10 years. Infants were defined as HIV exposed but uninfected if they were HIV uninfected, but the mother was HIV infected. HIV infection was confirmed by PCR in children aged <18 months. For all HIV-infected individuals, specimens were collected for CD4+ T cell and quantitative HIV viral load testing. Patients newly diagnosed with HIV were referred to the local clinic for assessment and initiation of antiretroviral treatment.

2.5 Environmental assessment

Environmental monitoring was conducted in a convenience subset of 150 households for one week each year during summer and winter. Respirable particulate matter <4-µm diameter (PM4) concentrations were measured indoors using a stationary photometric monitor (DustTrak II Model 8530, TSI Incorporated, Shoreview, MN, USA) and gravimetric filter sampling to measure the quantity of airborne particulate matter. During the same period, one member of each household carried a personal exposure monitoring device (SidePak AM510, TSI Incorporated, Shoreview, MN, USA) throughout the daytime. Indoor carbon dioxide and ambient PM4 (ES-642, MetOne Instruments, Inc, Grants Pass, OR, USA) levels were measured during 2018. Thermochron® iButton sensors (Maxim Integrated, San Jose, California, USA) were located in the indoor (all households) and ambient (subset) environments to measure temperature (Model DS1921G-F5) and relative humidity (Model RS1923I-F5 in 2018) concurrently with the air pollution monitoring in 2016 and continuously during 2017–2018.

2.6 Housing quality survey

Information on housing type, construction, materials and condition, water sources, water security and water storage, fuel use and expenditure for cooking, space and water heating, waste removal services, visible dampness and smoking practices was collected annually for all households.

2.7 Proximity and contact study

In 2018, four surveys of household contact using proximity monitors (http://www.sociopatterns.org) were conducted to capture information on intra-household contact patterns for three seasons (summer, autumn and winter). To measure contacts of participants of the study outside the home, participants were interviewed by field workers to complete a contact diary and time-use questionnaire for one day between August and October 2018.

2.8 Costing survey

We surveyed all symptomatic household members during August–October 2018 to assess cost of medically attended and non-medically attended illness episodes.

Outcomes	Objectives Primary<br><br>To estimate the community burden of influenza and RSV, including: (i) the incidence of influenza and RSV infection in the community; (ii) the symptomatic fraction associated with influenza and RSV infection; (iii) the severity associated with symptomatic infections; and (iv) the fraction of individuals with symptomatic infection seeking medical care.<br>To assess the transmission dynamics of influenza and RSV infections in the community, including: (i) the estimation of the household secondary infection risk (SIR), serial interval and length of shedding; (ii) the estimation of transmission of infection between age groups within the household and possibly the community; and (iii) the estimation of the effective reproductive number (Rt) and its variation over time in the community.<br>Secondary (selected)<br><br>To estimate the symptomatic fraction, the severity associated with symptomatic infections and the fraction of individuals with symptomatic infection seeking medical care among influenza or RSV positive cases by HIV serostatus and age.<br>To estimate the SIR and length of shedding of influenza and RSV among HIV-infected and HIV-uninfected index cases and the rate of acquisition of influenza and RSV infection among HIV-infected and HIV-uninfected household members.<br>To assess the role of asymptomatic infections in the household transmission of influenza and RSV.<br>Determine the heterogeneity of influenza and RSV virus strains within household clusters and describe viral evolution within and between households as well as the association between virus strains and the duration of virus shedding and HIV status<br>To describe and compare nasopharyngeal pneumococcal loads in healthy individuals by age and over time, and how the loads may be altered by respiratory viral infection, HIV-status and vaccination-status<br>To identify the prevalence and duration of B. pertussis colonization within the community by age group, vaccine status and over time, the transmission dynamics within a household, and the proportion of individuals that develop symptomatic infection<br>To determine the prevalence of N. meningitidis and C. diphtheria colonization within the community by age group at a single point in time each year<br>To measure the annual incidence of tuberculosis infection in individuals living in study households and assess risk factors (including incident and prevalent HIV) for acquiring tuberculosis infection.<br>To ascertain the impact of housing quality, fuel use and indoor air quality and selected measures of ventilation on tuberculosis infection and on household transmission of respiratory viruses and bacteria Methods: We will conduct a household-level community cohort study in a rural community in Mpumalanga Province where health and demographic surveillance (HDSS) is conducted (the Agincourt HDSS surveillance site) and selected urban areas in the Matlosana Muncipality in North West Province. Prospective, hospital-based surveillance for influenza- and RSV-associated severe acute respiratory illness has been conducted at these sites since 2010. The prospective cohort study will be conducted for 3 consecutive years, from 2016-2018, to include 3 consecutive RSV and influenza seasons. Approximately one hundred households (50 households per site, with an expected average number of household members of 5 to recruit a total of 500 enrolled individuals) will be randomly selected within the study population each year and consented to participate to the study. For each household member, serum will be collected quarterly (January-February, April-May, September-October and November-December) including before and after the RSV (February to May) and influenza (May to September) seasons and a final blood draw at the end of the year. The demographic characteristics of the household members will be collected on enrollment. Starting before the RSV and influenza seasons, upper respiratory tract samples will be collected twice-a-week by trained study nurses from all household members irrespective of the presence of respiratory symptoms throughout the year. During each visit the development of symptoms (using a severity score) and healthcare seeking behavior of symptomatic cases will be recorded. In addition, during winter at one household visit, an oropharyngeal swab will be collected from each individual for testing for meningococcus and diphtheria. Nasopharyngeal samples will be tested for the detection of RSV or influenza virus and pneumococcus and B. pertussis infection by real-time reverse-transcriptase polymerase chain reaction (rt-PCR). Blood samples will be tested for rise in RSV, influenza and pertussis antibody titers. From consenting patients, blood samples will be tested for HIV infection. For tuberculosis infection, we will do a tuberculin skin test (TST) at baseline and the end of the first year and annually thereafter in people are found to be TST negative at their previous test. HIV testing will be offered to all participants at baseline and then six monthly thereafter. All participants will have a sputum sample taken for culture at baseline and thereafter symptom screened for symptoms of tuberculosis monthly, and investigated with Xpert Mtuberculosis/Rif if symptoms of tuberculosis are present.

Plants

Seed stocks	NA
Novel plant genotypes	NA
Authentication	NA
