# Supplementary material for: The roles of pre-season immunity, age, viral shedding, and community exposures in shaping influenza household transmission dynamics
Source: Nat Commun. 2026 Jul 30;17:9238. doi: 10.1038/s41467-026-76037-x (PMC13526887; doi:10.1038/s41467-026-76037-x)
Supplement: Supplementary file 3 [file 41467_2026_76037_MOESM3_ESM.pdf]

nature portfolio

Peer Review File

The roles of pre-season immunity, age, viral shedding, and community exposures in shaping influenza household transmission dynamics

Corresponding Author: Dr Kaiyuan Sun

This file contains all reviewer reports in order by version, followed by all author rebuttals in order by version.

Version 0:

Reviewer comments:

Reviewer #1

(Remarks to the Author)
Thank you for the opportunity to review this manuscript – I enjoyed reading it. This manuscript utilises data from the PHIRST study in South Africa; this is a comprehensive dataset involving systematic testing (including asymptomatic individuals) that enables quantitative analysis of the factors driving influenza dynamics and facilitates comparison between subtypes/lineages. While some of the findings of the manuscript are interesting, my main concern is that I found the methodological details (almost entirely in the Supplementary Information) unclear to the point that they were hard to check. A secondary concern is that I was not completely convinced by some of the results, although I think this issue can be offset by suitable caveats being stated in the manuscript. Overall, I am positive about this manuscript at the general level, until I start digging around for details about the analysis. If the explanations of the methods can be improved, and caveats are included with the results as needed, then this manuscript may be suitable for publication (subject to the methods being appropriate when they are presented in a way that can be checked more easily). However, currently I think that a substantial amount of work still needs to be done to present the content of the manuscript more clearly.

I should also add that, although I did not run the code in the Github repository, it looks to me like it is very well documented, which is excellent.

Major comments:

The presentation of the models in the Supplementary Material is confusing and should be checked carefully. For example, in line 75 and Supp Fig 1, it seems that Delta Ct defines the minimum Ct of the infection episode (i.e., the Ct at the highest point in Supp Fig 1). However, in line 79, the authors present an equation for temporal changes in (the expected value of) Delta Ct, which I thought was the (fixed) minimum value. Should this be an equation for Ct, rather than Delta Ct? But line 77 also describes temporal changes in Delta Ct – so I am confused! The Supplementary Material would benefit from rewriting and careful checking (rather than just fixing the specific instances of confusion that I point out here).

Similarly, there should be checks for consistency throughout the Supplementary Material. For example, what is the variable “t” in the equation for the expected Ct? In the later transmission model, t is calendar time, but in the Ct model it must be something else – given the negative values of t, is it time since the minimum Ct in that infection episode? This should be clarified. I note that, in Fig 2A in the main text, the time values shown are times since minimum Ct, but this is not the case in Supp Fig 1, where t=0 appears after min Ct has been reached. I recommend that the authors use different notation for calendar time (in the later transmission model) and however they choose to define t in the Ct model, and also ensure that precisely what t refers to in the Ct model is clearly defined (and is consistent between the equation, Supp Fig 1 and Fig 2A in the main text).

Supp Material, line 87. Why was sigma held constant at 1? Couldn’t this be fitted to the data? Similarly, in line 98, why were the values 37/2 and 37/6 chosen (and other specific values around this point in the Supp Material)? Generally, please can more justification be provided for the modelling choices.

Supp Material, lines 122-124. “These parameter priors and distributions were chosen based on a series of sensitivity

analysis. Those provided here resulted in the best observed model fit based on the Gelman R-hat statistic and the number of divergent transitions." While the Gelman-Rubin statistics is relatively well known, the concept of divergent transitions is perhaps more specialist; in my view, both of these concepts should be summarised for the non-expert reader. More importantly, however, if sensitivity analyses were undertaken to determine the priors and/or any other distributions, then these sensitivity analyses should be reported fully (with figures plotted and relevant quantitative results quoted – e.g. R-hat values etc) to allow the reader to assess the results.

Please provide full details about the regression analysis of factors shaping viral load shedding characteristics (in particular, the relevant equation(s)).

Lines 196-199. I am concerned that the household data may be too sparse to reliably inform estimates of community prevalence. I note from the Supp Material that the estimated prevalence data were smoothed using a rolling window, but it would be useful to include time series plots of estimated prevalence for each subtype/year/location (probably in the supplement).

"The steepest age gradient in the titers was found between the ages of 0 and 10 years for both influenza A subtypes, while the increase was less pronounced for B/Victoria and spreads over a broader age range of 0 to 25 years for B/Yamagata." On first reading, I found this to be an interesting result and wondered whether this aligned with findings from previous studies (I know, for example, that infection severity tends to have a steeper age gradient for A than B, albeit over larger age ranges); can the authors comment? When I later came to Fig 1, however, I was a bit concerned that the fits are not that convincing – the splines are fitted through highly variable data, so is it really appropriate to compare those fits?

Apologies if I missed it, but I do not recall reading about the method used to fit the splines through the data in Fig 1 or Supp Fig 9, what order the fitted polynomials are or any other details?

"For A(H1N1)pdm09 infections, higher pre-season HAI titers correlated with shorter durations of shedding, with a 2.1-day reduction in duration time for those with a titer of at least 40 compared to those with lower titers [95% confidence interval (CI): -3.51 - -0.66 days]. This was not seen for other subtypes." Can the authors hypothesise why this was not the case for other subtypes?

"no significant association was found between titers above 40 and viral load reduction for any subtype or lineage". Presumably, this was a surprising result. Do the authors have a hypothesis for why this might have been the case?

"For both B/Victoria and B/Yamagata infections, larger household size was associated with a decreased risk of infection acquisition". Is the idea here essentially that larger household sizes actually correspond to a higher absolute infection risk (due to a higher household FOI) – just that the risk is not as high as might be expected based on FOI alone? If so, this should be unpacked more by the authors to avoid readers thinking that the infection risk is lower in larger households! This comment also applies in lines 403-404. If there really is an overall lower infection risk in larger households (even considering both FOI and household size), this should be explained (a dilution effect surely is not a sufficient explanation, as I would expect this to be counteracted by the higher FOI). In addition, the authors could explain how the effect of household size interacts with the household force of infection when they introduce the transmission model in the Supp Material.

"A 10-unit increase in shedding intensity, as indicated by the Ct value, was associated with approximately a 160% increased risk for A(H1N1)pdm09, 70% for A(H3N2), 90% for B/Victoria, and 50% for B/Yamagata." Can the authors speculate about possible reasons for these values being so different for different subtypes? In general, more discussion about possible explanations for the authors' results would strengthen the manuscript substantially.

Lines 359-374. The results relating to the effect of age on infection risk between subtypes are interesting. When the authors claim that if contact rates were the dominant factor then similar patterns would be expected between subtypes, I wondered whether this could also be affected by the lack of power due to the limited number of B/Yamagata infections? When the authors note that age differences between subtypes are indicative of age-specific immune responses, I think it is worth noting that this could be due to other age-specific factors (not simply average number of contacts) that were also not considered in the transmission model. As one example, if there is substantial heterogeneity in the number of contacts between individuals of the same age, then this could lead to some subgroups of the population appearing to have higher infection risks in under 11s, even if on average in the entire population 12-18 year olds have more contacts.

As hinted at above, the fitted splines in Fig 1 and the fitted triangles in Fig 2 are not especially convincing. I appreciate that these are average fits, but the data are highly variable, so I am not convinced by attempts to compare relatively small differences in these average values.

In Fig 2A, lots of dots appear that are not on the graphs! For example, there are dots off the left of the bottom left panel, as well as in between the panels.

Supp Material, line 79. I think there is an error in the definition of Delta Ct (or Ct??) between times tp and tp+omega_c? Given that Ct is not constant there, then as a minimum there should be a dependence on t? These equations (and all other methodological details) need careful checking.

Minor comments:

Supp Material, lines 95-101. What are the numbers in square brackets? The notation here should be explained.

Did the authors rigorously account for censoring (i.e. false negative test results) when fitting the shedding model?

Supp Material, line 213. The formatting of this equation is not good, with squares appearing (empty equation fields) – this should be fixed.

Supp Material, lines 219-230. I am not sure the stated formulation makes sense for the non-binary categorical variables. Also, is the household size effect assumed to be monotonic?

Supp Material, lines 143-144. "Year was incorporated as a random effect for all except B/Yamagata". It might be worth reminding the reader here that B/Yamagata only circulated in one year (this is stated in the main text, but I forgot this by the time I was reading the Supp Material). Also, how was year incorporated as a random effect in the model – can the authors explain the method in the Supp Material?

When the authors introduce the transmission model in the Supplementary Material, they include terms for community transmission and household transmission. Doesn't this pre-suppose that these two locations are the main sources of transmission? I do not know much about the locations of the PHIRST study, but certainly in some countries school-based transmission is a key driver. Some rationale for focussing solely on community and household transmission, rather than e.g. school (or any other specific location) transmission, would be beneficial.

Supp Material, lines 231-233. "Observations were created for each day in the influenza season, and we assigned a binary dependent variable indicating a 0 when no infection has occurred and a 1 on the estimated day of infection acquisition". Was this done independently of the transmission model (e.g. based on individuals' measured viral loads), or was the day of infection acquisition estimated using the transmission model? I suspect it is the former, but this should be explained more clearly.

Line 118. "Blood serum samples were collected at least once prior to the influenza season". I think this should say "each influenza season"?

Line 163-164. "The onset of infection was inferred to be one day prior to the first day of shedding". First of all, by saying "inferred", I assumed that this was a result of the authors' analysis – whereas in fact I think they took this value from the literature. Second, might this be expected to vary between individuals?

Lines 241-243. "The variance of the clearance…". Should this be mean rather than variance?

Line 295. I think ">1:40" is a typo and should just be ">40".

Lines 351-352. "however modeling titers on a continuous scale supports a relationship (Supplementary Fig 8)." I would consider talking the reader through Supp Fig 8 in a bit more detail to help explain this result; I found this confusing.

Table 1. Please specify what the numbers in brackets represent. Is the value for males in 2016 deliberately obscured?

In the figure captions, the authors claim that asterisks denote significance levels. It is slightly pedantic (sorry), but aren't the asterisks actually indicative of p-values rather than significance values? (i.e. *** indicates a p-value less than the significance level of 0.001 etc). The wording here should perhaps be made more technically correct (although I appreciate that it is already clear what the authors mean).

Fig 3. Why is there no uncertainty in the shedding duration and minimum Ct for the reference groups? If the reference values are the same in each category, should e.g. the shedding durations in different age groups be interpreted as being age-specific estimates for HIV-negative females with pre-season HAI titre <40?

Fig 4. Is this really a "schematic"? If I understand the figure correctly, it is actually a visualisation of results from the fitted model, so I am not sure that "schematic" is the correct word to use? (I do not have a strong feeling about this though, so please feel free to ignore!)

Fig 5. Should the caption and/or figure specify that hazard ratios for community FOI are (I think) with respect to a 1% change in prevalence?

(Remarks on code availability)

Reviewer #2

(Remarks to the Author)
This is a household cohort study conducted in rural and urban communities in South Africa across 3 influenza seasons to inform influenza transmission models. 327 households (1684 individuals) were enrolled with follow-up, averaging 110 new households enrolled each year. Serum was collected prior to influenza season to measure antibody titers against circulating

strains (HAI)/prior immunity. Respiratory samples were collected twice weekly, regardless of symptoms, to identify influenza infections (rRT-PCR). The authors leveraged this PHIRST study and comprehensive data collected to examine the role of prior immunity, age, and exposure intensity on risk of influenza infection/acquisition in an unvaccinated population in low- and middle-income country.

This is nicely presented, clearly written manuscript. I am unable to comment on the specifics of the modeling (calibration, etc), but the approach, assumptions, and model inputs are appropriate and clearly described. The major strength and contribution of this paper is that the transmission models are subtype/lineage-specific and the models include and assess measures for prior immunity. Preseason HAI titers were correlated with shedding duration only for A(H1N1)pdm09; preseason HAI titers ≥1:40 were protective against infection (except against for B/Yamagata). Higher viral shedding was associated with higher risk of infection. Younger age was consistently (and independently) associated with longer shedding duration, higher viral loads, and higher risk of infection. The variability in the results highlights the challenges with influenza and the need to examine influenza by subtype/lineage whenever possible. The authors interpretation of the data and conclusions are valid. The presentation of data, especially the forest plots are very nice. I do not have any concerns with the methodology, data analysis, interpretation, or conclusions.
This work is remarkable and adds to our understanding of influenza transmission in an unvaccinated population in rural and urban communities in South Africa.

minor comments
- The authors mention the PHIRST study had a high prevalence of asymptomatic infections, did the authors examine or are the authors able to examine the role of asymptomatic infections in shaping household transmission dynamics?
- Line 295 – typo? I think “>1:40” should be “≥40”

(Remarks on code availability)

Reviewer #3

(Remarks to the Author)
In this study, the authors presented their quantitative analysis that estimated the household transmission risk of influenza viruses in two cohorts of participants in South Africa. The authors provided a nice summary of the estimated viral shedding kinetics (Fig. 2) and predictors of infection risk (Fig. 3). They developed a detailed household transmission model to assess how influenza virus exposure intensity, age, and serum HAI titers influence the risk of infection acquisition (Figs. 4 and 5).

I appreciate the great efforts of the authors in organizing the intensive samplings of nasopharyngeal swabs in the PHIRST cohorts. This study design is unique and not easy in implementation. The estimates of infection risk predictors and the relationship between pre-season serum titer and correlates of protection against infection are useful to improve the understanding of household transmission of influenza in South Africa. The authors performed the analysis for all four influenza subtypes/lineages.

There are a few points not very clear in the current manuscript:

1. The influence of school schedules and holiday seasons could be discussed. The authors found that children had higher risk of infection. It looks unclear whether children cases were clustered in time or randomly distributed over the study period.

2. I don’t quite understand the influence of HIV status on the risk of influenza infection. It would be perfect if the authors could add some biological interpretation on this.

3. In line 286, the authors estimated that “a one percent increase in influenza prevalence in the population was associated with a 30-100% increase in risk”. This estimate may suggest a strong influence of community FOI on the household risk of infection. This looks a bit overestimated.

4. In the paragraph starting with line 304, the authors identified the influence of household size on influenza B virus but not influenza A virus. The underlying mechanism is not very clear to make this claim.

5. In line 160-161, “at a population mean and individual (per infection episode) level.” This statement looks confusing.

(Remarks on code availability)

Reviewer #4

(Remarks to the Author)
I co-reviewed this manuscript with one of the reviewers who provided the listed reports. This is part of the Nature Communications initiative to facilitate training in peer review and to provide appropriate recognition for Early Career Researchers who co-review manuscripts.

(Remarks on code availability)

Version 1:

Reviewer comments:

Reviewer #1

(Remarks to the Author)
Thanks to the authors for their responses to our initial set of comments. In general, the authors have provided comprehensive answers to our questions – thank you for that. However, there are still some aspects that we are not convinced about. We therefore think that more work should be done to improve this manuscript before it is considered for publication.

Major comments:

1. The authors use Supplementary Figure 11 to conclude that "the cumulative household infection risk (defined as the fraction of household members infected during a given season) varied across households of different sizes (Supplementary Fig 11)." Looking at the trend lines in that figure, it looks to us like the infection risk does not vary much with household size. We therefore do not agree with the authors' interpretation.

2. Supp Fig 11. The authors argue that fitting trend lines through scatter plot data is commonplace. We agree, and do not generally have an issue with this. However, looking at the dots here, the data points are really scattered – so do the authors really believe that that the observed trends can be trusted?

3. Supp Fig 11 (again!). The caption seems to suggest that there ought to be 4 points for each x value from 3 to 9, but some x values have 5 points ("Each point represents the observed cumulative infection risk for a given household size and influenza virus subtype or lineage."; this suggests to us that at each household size there ought to be one point per subtype?) All the y-values seem to be multiples of 1/(household size), so we wonder if a dot is actually being plotted for every individual household (with lots of overlapping dots, so that there are lots of data points there that cannot be seen). If so, the authors should find a way to visualise the data more clearly (maybe with dots with areas corresponding to the number of points there), and the caption should be corrected.

4. Fig 1. Again, the authors argue that fitting splines through scatter plot data is commonplace. However, when the data are as variable as they appear in Fig 1, we find it hard to be convinced that any trend lines are genuinely trends as opposed to simply being reflective of smoothed lines through a quantity that is highly variable. We think that it might be beneficial for the authors to also visualise a summarised version of the data without smoothing, to assess how reasonable the observed trends are. For example, the authors could plot the average of the data points within age bands to demonstrate that this shows a similar trend (without applying any smoothing). If this displays a similar trend, this would make the inferred trends more convincing; if not, then the trends should be removed.

5. In our previous review, we asked about the previous claim that "A 10-unit increase in shedding intensity, as indicated by the Ct value, was associated with approximately a 160% increased risk for A(H1N1)pdm09, 70% for A(H3N2), 90% for B/Victoria, and 50% for B/Yamagata." In the authors' response, they note that in their revised analysis there is more consistency between subtypes. However, they go on to explain potential mechanisms underlying the subtype specific differences. In the manuscript, however, it seems that the CIs now overlap substantially between subtypes; so we would simply argue that the values were similar for all subtypes rather than trying the explain why they are different (since the authors' Bayesian analysis does not support that a difference exists).

6. While the authors have improved their explanation of the modelling approach taken, we are still not completely convinced about all of the aspects. For example, in Supplementary Figure 1 (and in the text), t is described as calendar time. But doesn't t instead represent time within an individual's infection (rather than overall calendar time)? If not, then when t=0, which may be months before an individual becomes infected, then following the authors' explanation in the Supplementary Information literally then their viral load is drawn from max(0, Normal(0,sigma)) – which seems a little odd?

7. We may be misunderstanding, but the authors seem to use the household data to determine the community prevalence in their model. Then, they analyse the interplay between community prevalence and household exposure, concluding that the infection risk in households was high when the community prevalence was high. This conclusion is in fact simply the assumption that the authors made when they chose to set the community prevalence based on the household cohort data? This seems to be circular reasoning to us.

Minor comments:

1. In our previous review, we asked whether the authors accounted for false negative test results when fitting the model. From the authors' response, it is still unclear whether they accounted for false negatives; it sounds like the authors fitted the model to observed Ct values, thereby not accounting for false negatives? Please confirm.

2. Fig 1. Are the black dotted lines also smoothed? If so, this should be clarified in the caption (with the method of smoothing stated)?

3. One of the questions we asked about in our original review is where the priors were chosen from. While the authors have now attempted to explain this, we think that their explanation that they “were informed primarily by published viral kinetics models and relevant influenza human challenge studies [1,6,7]” is still quite vague. This could be made more precise; exactly which priors came from which study, and how were they chosen?

4. Some of the text in the Supp Info is in equation font (e.g. “days” in “omega_p < 15 days”) rather than in standard font.

5. “To improve computational efficiency, constrained the parameters to biologically plausible upper bounds: $\omega < 15\ days$, $\omega < 30\ days$, and $\Delta Ct < 37$, as values beyond these thresholds are considered implausible for influenza viral kinetics”. References would be useful here to support these choices. Also, we think you are missing the word “we” before constrained?

6. “higher B/Yamagata titers did associate with a reduce infection risk”. This should say “reduced”?

(Remarks on code availability)
We did not review the code in this review (we did access the code in our original review).

Reviewer #3

(Remarks to the Author)
The authors have fully addressed my comments in the revised manuscript. My pleasure to recommend the acceptance of this submission.

(Remarks on code availability)

Reviewer #4

(Remarks to the Author)
I co-reviewed this manuscript with one of the reviewers who provided the listed reports. This is part of the Nature Communications initiative to facilitate training in peer review and to provide appropriate recognition for Early Career Researchers who co-review manuscripts.

(Remarks on code availability)

Version 2:

Reviewer comments:

Reviewer #1

(Remarks to the Author)
Thank you to the authors for responding to our comments on the revised version of the manuscript. The authors have amended the major issues in the original version of the manuscript. We have two minor remaining comments; when these are addressed, we are content that the authors will have provided responses to all of our original points.

1. I am afraid that the description of the methods is (we think) still wrong. For example, in the equation for Delta(Ct) in line 88 of the Supplementary Information, when t is between t_p and t_p+omega_c, there is no dependence on t. We think this ought to be a linear change in Delta(Ct) (so must surely vary with t). I guess this is just a typo, but it points towards the authors needing to still check their description of the methods very carefully. We have not re-reviewed the manuscript in detail, so we would recommend that the authors please check the Supplementary Information again very carefully.

2. Based on the authors' response to our previous comment 6, it seems like the observation model is max(0, Normal(0,sigma)) far away from the peak viral load, including calendar times far preceding the infection episode. This means that, when there is no virus, there is still a substantial chance of a false positive test result (potentially even 50%, if any observed Ct is indicative of an infection). Is this correct? Apologies if we are misunderstanding something here. This assumption should at the very least be made very explicit in the manuscript, since a (possibly 50%?) chance of a false positive test result seems somewhat high.

Thanks again for amending your manuscript, and we hope that our reviews have been constructive. We believe that the manuscript is now much improved.

(Remarks on code availability)

Reviewer #4

(Remarks to the Author)
I co-reviewed this manuscript with one of the reviewers who provided the listed reports. This is part of the Nature Communications initiative to facilitate training in peer review and to provide appropriate recognition for Early Career Researchers who co-review manuscripts.

(Remarks on code availability)

Open Access This Peer Review File is licensed under a Creative Commons Attribution 4.0 International License, which permits use, sharing, adaptation, distribution and reproduction in any medium or format, as long as you give appropriate credit to the original author(s) and the source, provide a link to the Creative Commons license, and indicate if changes were made.
In cases where reviewers are anonymous, credit should be given to 'Anonymous Referee' and the source.
The images or other third party material in this Peer Review File are included in the article's Creative Commons license, unless indicated otherwise in a credit line to the material. If material is not included in the article's Creative Commons license and your intended use is not permitted by statutory regulation or exceeds the permitted use, you will need to obtain permission directly from the copyright holder.
To view a copy of this license, visit https://creativecommons.org/licenses/by/4.0/

Reviewer #1 (Remarks to the Author):

Thank you for the opportunity to review this manuscript – I enjoyed reading it. This manuscript utilises data from the PHIRST study in South Africa; this is a comprehensive dataset involving systematic testing (including asymptomatic individuals) that enables quantitative analysis of the factors driving influenza dynamics and facilitates comparison between subtypes/lineages. While some of the findings of the manuscript are interesting, my main concern is that I found the methodological details (almost entirely in the Supplementary Information) unclear to the point that they were hard to check. A secondary concern is that I was not completely convinced by some of the results, although I think this issue can be offset by suitable caveats being stated in the manuscript. Overall, I am positive about this manuscript at the general level, until I start digging around for details about the analysis. If the explanations of the methods can be improved, and caveats are included with the results as needed, then this manuscript may be suitable for publication (subject to the methods being appropriate when they are presented in a way that can be checked more easily). However, currently I think that a substantial amount of work still needs to be done to present the content of the manuscript more clearly.

I should also add that, although I did not run the code in the Github repository, it looks to me like it is very well documented, which is excellent.

We greatly appreciate the reviewer's positive assessment of our manuscript and the detailed, constructive feedback, particularly on the methodological details, that helped further clarify and improve our manuscript.

We acknowledge the reviewer's concern regarding the clarity of the methodological details presented in the Supplementary Information. In response, we have undertaken a major revision of the Supplementary Information to improve the clarity, structure, and transparency of our analyses. We have addressed the reviewer's specific comments in the revised manuscript and provided point-by-point responses below.

Major comments:

The presentation of the models in the Supplementary Material is confusing and should be checked carefully. For example, in line 75 and Supp Fig 1, it seems that Delta Ct defines the minimum Ct of the infection episode (i.e., the Ct at the highest point in Supp Fig 1). However, in line 79, the authors present an equation for temporal changes in (the expected value of) Delta Ct, which I thought was the (fixed) minimum value. Should this be an equation for Ct, rather than Delta Ct? But line 77 also describes temporal changes in Delta Ct – so I am confused! The Supplementary Material would benefit from rewriting and careful checking (rather than just fixing the specific instances of confusion that I point out here).

Similarly, there should be checks for consistency throughout the Supplementary Material. For example, what is the variable "t" in the equation for the expected Ct? In the later transmission model, t is calendar time, but in the Ct model it must be something else – given the negative values

of t, is it time since the minimum Ct in that infection episode? This should be clarified. I note that, in Fig 2A in the main text, the time values shown are times since minimum Ct, but this is not the case in Supp Fig 1, where t=0 appears after min Ct has been reached. I recommend that the authors use different notation for calendar time (in the later transmission model) and however they choose to define t in the Ct model, and also ensure that precisely what t refers to in the Ct model is clearly defined (and is consistent between the equation, Supp Fig 1 and Fig 2A in the main text).

We thank the reviewer for pointing out these issues and the resulting confusion in our model equations and variable definitions. To ensure consistency throughout the manuscript, we have now clearly defined ΔCt as the difference between the limit of detection threshold (Ct_{lod}= 37) and the observed Ct value, which serves as a proxy for viral shedding intensity. We have thoroughly revised the Supplementary Information to ensure that all downstream analyses, including the formulation of the viral shedding kinetics model and the construction of the household force-of-infection in the transmission model, are consistently based on this definition of ΔCt.

To further improve clarity, we have also revised the notation of the time variable t. In the viral shedding model, t now represents calendar time, with the timing of peak shedding (t_p) modeled as an estimated parameter corresponding to the calendar day of peak viral load. This ensures that t has a consistent interpretation across both the viral shedding and household transmission models. The revised Supplementary Information and figure legends have been carefully updated to reflect these above clarifications (Session 2 and Session 3).

When presenting the average shedding kinetics for each subtype in Figure 2 of the main text, we transformed the fitted model of ΔCt back to Ct values to ensure direct comparability with the observed RT-PCR data and to facilitate interpretation. For visualization, we further centered the average shedding trajectories of each influenza subtype or strain at the model-estimated peak shedding time—that is, time since peak shedding is defined as $\tau = t - \overline{t_p}$, where $\overline{t_p}$ represents the mean estimated timing of peak viral load. This transformation and alignment procedure are now described in detail in Supplementary Methods Section 2.3 (lines 196-203) as follows:

> *"In Figure 2A and 2E, when visualizing the average viral shedding kinetics for each subtype or strain, we first transformed the model-predicted ΔCt values back to Ct by adding the limit of detection threshold (Ct_{lod}) to enable direct comparison with the observed RT-PCR data. We then rescaled the temporal axis from calendar time (t) to time since peak shedding (τ), defined relative to the average timing of minimum Ct (i.e., the average timing of peak viral shedding, $\overline{t_P}$). These transformations are defined as follows:*
>
> $$Ct(\tau) = \Delta Ct(\tau) + Ct_{lod}$$
>
> $$\tau = t - \overline{t_P}$$ "

Supp Material, line 87. Why was sigma held constant at 1? Couldn't this be fitted to the data? Similarly, in line 98, why were the values 37/2 and 37/6 chosen (and other specific values around this point in the Supp Material)? Generally, please can more justification be provided for the modelling choices.

Supp Material, lines 122-124. "These parameter priors and distributions were chosen based on a series of sensitivity analysis. Those provided here resulted in the best observed model fit based on the Gelman R-hat statistic and the number of divergent transitions." While the Gelman-Rubin statistics is relatively well known, the concept of divergent transitions is perhaps more specialist; in my view, both of these concepts should be summarised for the non-expert reader. More importantly, however, if sensitivity analyses were undertaken to determine the priors and/or any other distributions, then these sensitivity analyses should be reported fully (with figures plotted and relevant quantitative results quoted – e.g. R-hat values etc) to allow the reader to assess the results.

We thank the reviewer for these helpful comments. In response, we revised the influenza shedding model to allow σ to be estimated directly from the data for each influenza subtypes/strains rather than being held constant at 1. For the updated model, we found the median posterior distribution of σ as 0.65 for A(H1N1)pdm09 (95% CI: 0.60–0.73), 1.00 for A(H3N2) (95% CI: 0.96–1.05), 1.25 for B/Victoria (95% CI: 1.14–1.39), and 0.96 for B/Yamagata (95% CI: 0.89–1.30), which are comparable to our initial assumption of fixing σ at 1. All subsequent results and figures derived from the shedding model have been updated to reflect the revised parameter estimates, our findings on viral shedding are robust and comparable to the previous choice of holding σ to 1.

We also clarified a source of confusion in the Supplementary Material regarding our choice of priors and the associated sensitivity analyses. Upon reviewing our lab notes, we found that the initial version of the shedding model inherited prior settings from our previous SARS-CoV-2 analysis [1]. However, these priors did not adequately capture the shedding kinetics of influenza viruses and resulted in poor model convergence. To address this, we consulted the literature from human challenge studies of influenza and redefined the priors to encompass the plausible range of influenza viral shedding dynamics, which substantially improved model performance and convergence. In the revised version, to avoid confusion, we simplified the description of the prior specification and now explicitly state that the priors were informed by influenza human challenge studies. Specifically, we have added the following clarification in the Supplementary Material lines 111-115:

> *"The priors of each of the population level mean parameters (μ_{ω_p}, μ_{ω_c}, $\mu_{\Delta C\ max}$) were specified as truncated normal distributions covering biologically plausible ranges of variation in viral proliferation duration (ω_p), clearance duration (ω_c), and peak viral shedding (ΔCt_{max}). These priors, were informed primarily by published viral kinetics models and relevant influenza human challenge studies [1,6,7]…"*

We also follow the reviewer's advice to expand the description of the model diagnostics by briefly summarizing the meaning of the Gelman–Rubin statistic (R-hat) and divergent transitions for non-expert readers. The updated description can be found in Supplementary Materials line 155-158 as follows:

> *"To evaluate the model fit, we analyzed the number of divergent transitions for each of the four model runs, relevant to the numerical instability in Hamilton Monte Carlo sampling and should ideally be zero, and the Gelman R-hat statistic for each posterior, a convergence diagnostic where values close to 1 indicate good mixing across chains."*

Please provide full details about the regression analysis of factors shaping viral load shedding characteristics (in particular, the relevant equation(s)).

We thank the reviewer for this comment. We have added the equations in Supplementary Materials lines 226-272 as follows:

"The specific equations of the regression models are as follows:

$$D_i = \beta_0^D + \beta_T^D x_i^T + \boldsymbol{\beta}_{\boldsymbol{A}}^{\boldsymbol{D}} \boldsymbol{x}_{\boldsymbol{i}}^{\boldsymbol{A}} + \boldsymbol{\beta}_{\boldsymbol{S}}^{\boldsymbol{D}} \boldsymbol{x}_{\boldsymbol{i}}^{\boldsymbol{S}} + \boldsymbol{\beta}_{\boldsymbol{HI}}^{\boldsymbol{D}} \boldsymbol{x}_{\boldsymbol{i}}^{\boldsymbol{HIV}} + b_Y^D x_i^Y$$

$$P_i = \beta_0^P + \beta_T^P x_i^T + \boldsymbol{\beta}_{\boldsymbol{A}}^{\boldsymbol{P}} \boldsymbol{x}_{\boldsymbol{i}}^{\boldsymbol{A}} + \boldsymbol{\beta}_{\boldsymbol{S}}^{\boldsymbol{P}} \boldsymbol{x}_{\boldsymbol{i}}^{\boldsymbol{S}} + \boldsymbol{\beta}_{\boldsymbol{HIV}}^{\boldsymbol{P}} \boldsymbol{x}_{\boldsymbol{i}}^{\boldsymbol{HIV}} + \boldsymbol{b}_{\boldsymbol{Y}}^{\boldsymbol{P}} \boldsymbol{x}_{\boldsymbol{i}}^{\boldsymbol{Y}}$$

Where:

D_i *– shedding duration for infection episode* i*, scalar term.*

P_i *– peak shedding intensity for infection episode* i*, scalar term.*

β_0^D *– regression intercept for shedding duration, scalar term.*

β_0^P *– regression intercept for peak shedding intensity, scalar term.*

β_T^D *– regression coefficient for the association between pre-season HAI titer and shedding duration, scalar term.*

β_T^P *– regression coefficient for the association between pre-season HAI titer and peak shedding intensity, scalar term.*

x_i^T *– individual's pre-season HAI titer for infection episode* i*, scalar term.*

$\boldsymbol{\beta}_{\boldsymbol{A}}^{\boldsymbol{D}}$ *– regression coefficients for the association between age group and shedding duration, vector term, with each element of the vector representing the coefficient of a specific age group.*

$\boldsymbol{\beta}_{\boldsymbol{A}}^{\boldsymbol{P}}$ – regression coefficients for the association between age group and peak shedding intensity, vector term, with each element of the vector representing the coefficient of a specific age group.

$\boldsymbol{x}_{\boldsymbol{i}}^{\boldsymbol{A}}$– individual's age categories for infection episode i, vector term, with each element of the vector representing a specific age category.

$\boldsymbol{\beta}_{\boldsymbol{S}}^{\boldsymbol{D}}$ – regression coefficients for the association between sex and shedding duration, vector term, with each element of the vector representing the regression coefficient of a specific sex category.

$\boldsymbol{\beta}_{\boldsymbol{S}}^{\boldsymbol{P}}$ – regression coefficients for the association between sex and peak shedding intensity, vector term, with each element of the vector representing the regression coefficient of a specific sex category.

$\boldsymbol{x}_{\boldsymbol{i}}^{\boldsymbol{S}}$– individual's sex categories for infection episode i, vector term, with each element of the vector representing a specific sex category.

$\boldsymbol{\beta}_{\boldsymbol{HIV}}^{\boldsymbol{D}}$ – regression coefficients for the association between HIV status and shedding duration, vector term, with each element of the vector representing the regression coefficient of a specific HIV infection status.

$\boldsymbol{\beta}_{\boldsymbol{HIV}}^{\boldsymbol{P}}$ – regression coefficients for the association between HIV status and peak shedding intensity, vector term, with each element of the vector representing the regression coefficient of a specific HIV infection status.

$\boldsymbol{x}_{\boldsymbol{i}}^{\boldsymbol{HIV}}$– individual's HIV status categories for infection episode i, vector term, with each element of the vector representing a specific HIV status.

$\boldsymbol{b}_{\boldsymbol{Y}}^{\boldsymbol{D}}$ – random effects on shedding duration by calendar year, vector term, with each element of the vector representing the random effect of a specific calendar year.

$\boldsymbol{b}_{\boldsymbol{Y}}^{\boldsymbol{P}}$ – random effects on peak shedding intensity by calendar year, vector term, with each element of the vector representing the random effect of a specific calendar year.

$\boldsymbol{x}_{\boldsymbol{i}}^{\boldsymbol{Y}}$– calendar year when infection episode i occurred, vector term, with each element of the vector representing a specific calendar."

Lines 196-199. I am concerned that the household data may be too sparse to reliably inform estimates of community prevalence. I note from the Supp Material that the estimated prevalence data were smoothed using a rolling window, but it would be useful to include time series plots of estimated prevalence for each subtype/year/location (probably in the supplement).

We thank the reviewer for this comment and agree that we should include a time series plot of estimated prevalence. We used the household data because the cohorts randomly selected households from the site populations, so infection prevalence in the cohorts should be representative of the general population. District level influenza surveillance data were very sparse and noisy and were not representative for community FOI.

We have now included a new set of time series plots in the Supplementary Information to illustrate the estimated community prevalence derived from the smoothed community FOI in the cohort data. These plots further stratify prevalence by age group, specifically distinguishing school-aged children (5–18 years) from other age groups, to examine whether infections clustered differently among children compared with adults. To provide additional context, we overlaid the South African school calendar on the plots (gray shading), noting that school attendance is compulsory for ages 7–15, while non-compulsory schooling encompasses the broader 5–18-year age band. The new figure is presented as Supplementary Fig. 10, shown below. Notably, influenza circulation in South Africa typically occurs during school terms, and there is no strong asynchrony in infection timing between school-aged children and the non-school-aged population.

Supplementary Fig. 10 Community prevalence within cohort informing community FOI

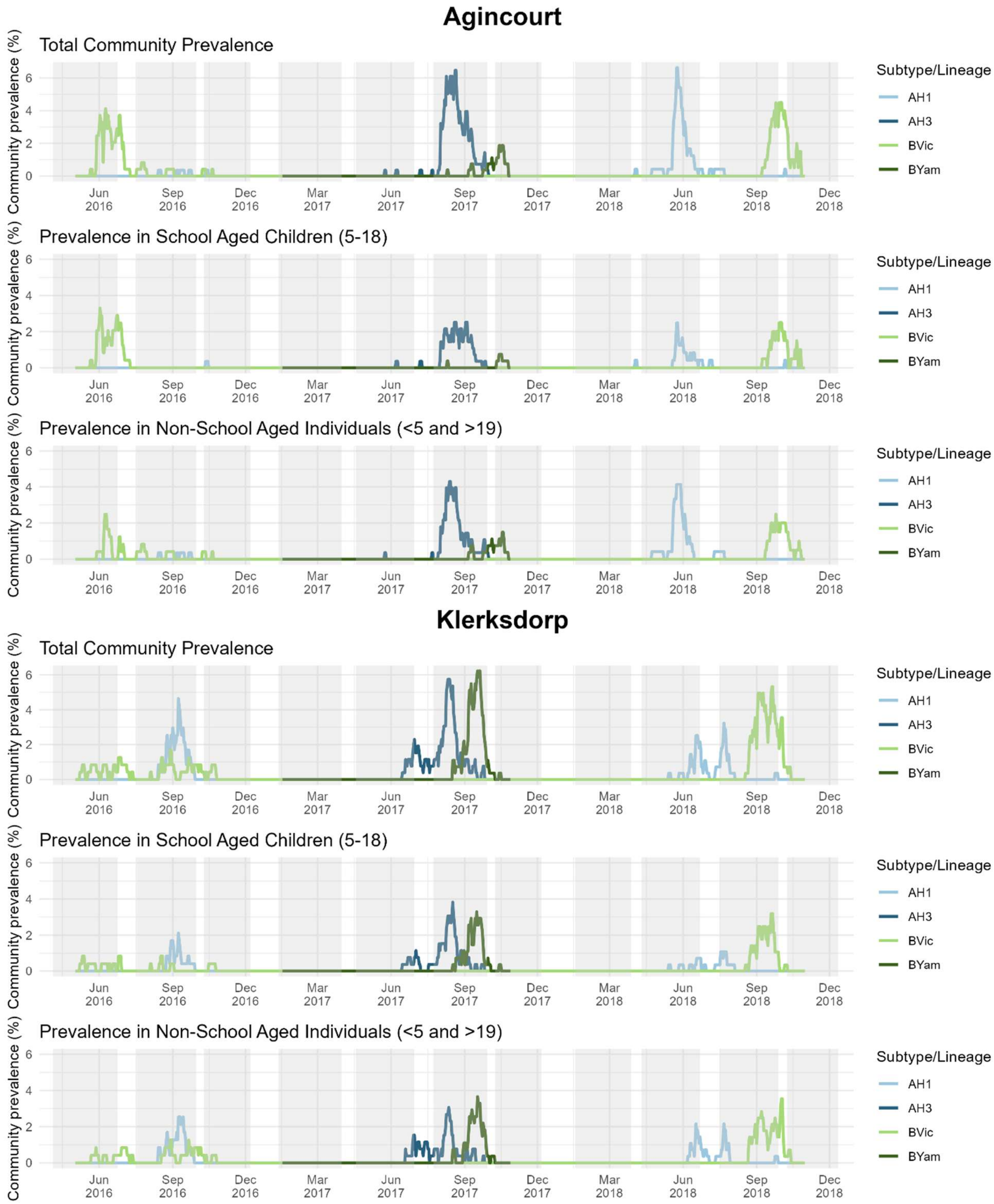

Each set of three panels represents the two cohort sites, Agincourt and Klerksdorp. The top panels of the sets show total community prevalence, calculated as the number of infections on a given day divided by the total cohort population size and smoothed over a 4-day window to account for

potential missing samples. The middle panels show prevalence restricted to school-aged individuals, and the bottom panels show prevalence among non-school-aged individuals. The colored lines correlate to the prevalence for each of the four subtypes/lineages. Community FOI, and thus the prevalence here, was only calculated for the years used in the transmission model for each subtype/lineage (2016 and 2018 for A(H1N1)pdm09 and B/Victoria, 2017 for A(H3N2) and B/Yamagata. The grey shaded regions in each figure indicate when school was in session.

We have now clarified our rationale for this choice of community FOI in the Supplementary Methods (line 335-344), as detailed below:

> *"The community FOI term was represented by the community-level prevalence of infection within the cohort at time t. This time-varying covariate was smoothed using a four-day moving average, corresponding to the maximum interval between consecutive sample collections. We used the cohort household data because households were randomly selected from the site populations, making cohort infection prevalence broadly representative of the community. Supplementary Fig. 10 shows time series of the smoothed community FOI, with separate figures for school-aged children (5–18) and other ages, with the school calendar indicated to provide context for outbreak timing. While school sessions could influence contact patterns, the community FOI shows no distinct timing differences, supporting its use as a reliable measure of population-level prevalence for the model."*

"The steepest age gradient in the titers was found between the ages of 0 and 10 years for both influenza A subtypes, while the increase was less pronounced for B/Victoria and spreads over a broader age range of 0 to 25 years for B/Yamagata." On first reading, I found this to be an interesting result and wondered whether this aligned with findings from previous studies (I know, for example, that infection severity tends to have a steeper age gradient for A than B, albeit over larger age ranges); can the authors comment? When I later came to Fig 1, however, I was a bit concerned that the fits are not that convincing – the splines are fitted through highly variable data, so is it really appropriate to compare those fits?

We thank the reviewer for this comment and agree that we should add details to suggest whether or not this was an expected result through reference relevant literature. Prior work has reported similar age-dependent antibody patterns, particularly for influenza A, which has been the primary focus of antibody landscape analyses to date [5,6]. Although fewer studies have directly compared antibody titers across age groups between influenza A and B viruses, some have suggested a consistent pattern of stronger and more sharply increasing antibody titers to influenza A during early childhood [7].

Among older adults, previous studies have shown that hemagglutination inhibition (HAI) titers against influenza B are generally lower than those against influenza A, consistent with our

findings[8]. Possible explanations for this difference include the more sporadic circulation of influenza B compared with influenza A, which could lead to less frequent exposure and, consequently, a more gradual increase in antibody titers among children. Additionally, the lower titers for influenza B may reflect the inferior persistence of antibodies against influenza B viruses relative to influenza A. We have incorporated the following hypothesis into the Discussion (lines 369-374) to contextualize these hypotheses:

> *"... As it relates to age, the observed titer patterns for influenza A indicate a rapid establishment of strain-specific antibody responses during early childhood, consistent with strong early-life imprinting to influenza A antigens [5]. In contrast, the broader and less pronounced gradient observed for influenza B likely reflects less frequent immune boosting due to lower infection rates, as well as the inferior persistence of antibodies against influenza B viruses compared with influenza A [7,8]."*

We would like to note that it is common practice to fit the mean titer trend by age despite variability within each age cohort [5,6], as such variability is expected in population-based cohort data. Infection acquisition is inherently stochastic, leading to differences in individual exposure histories, and immune responses also vary across individuals. Similar heterogeneity in antibody titers across ages has been reported in previous studies [5,8].

We agree with the reviewer that it is important to quantify and visualize how heterogeneity in titers changes with age. To address this, we have revised the figure to more explicitly illustrate variability in the data. Because infection and antibody boosting occur stochastically in the absence of vaccination, particularly among relatively immunologically naive children who have experienced few influenza seasons, the variability in titers gradually decreases with age. We have revised the figure to include a dotted black line of the standard deviation of the titers (see an example here for A(H1N1)pdm09 in line 459):

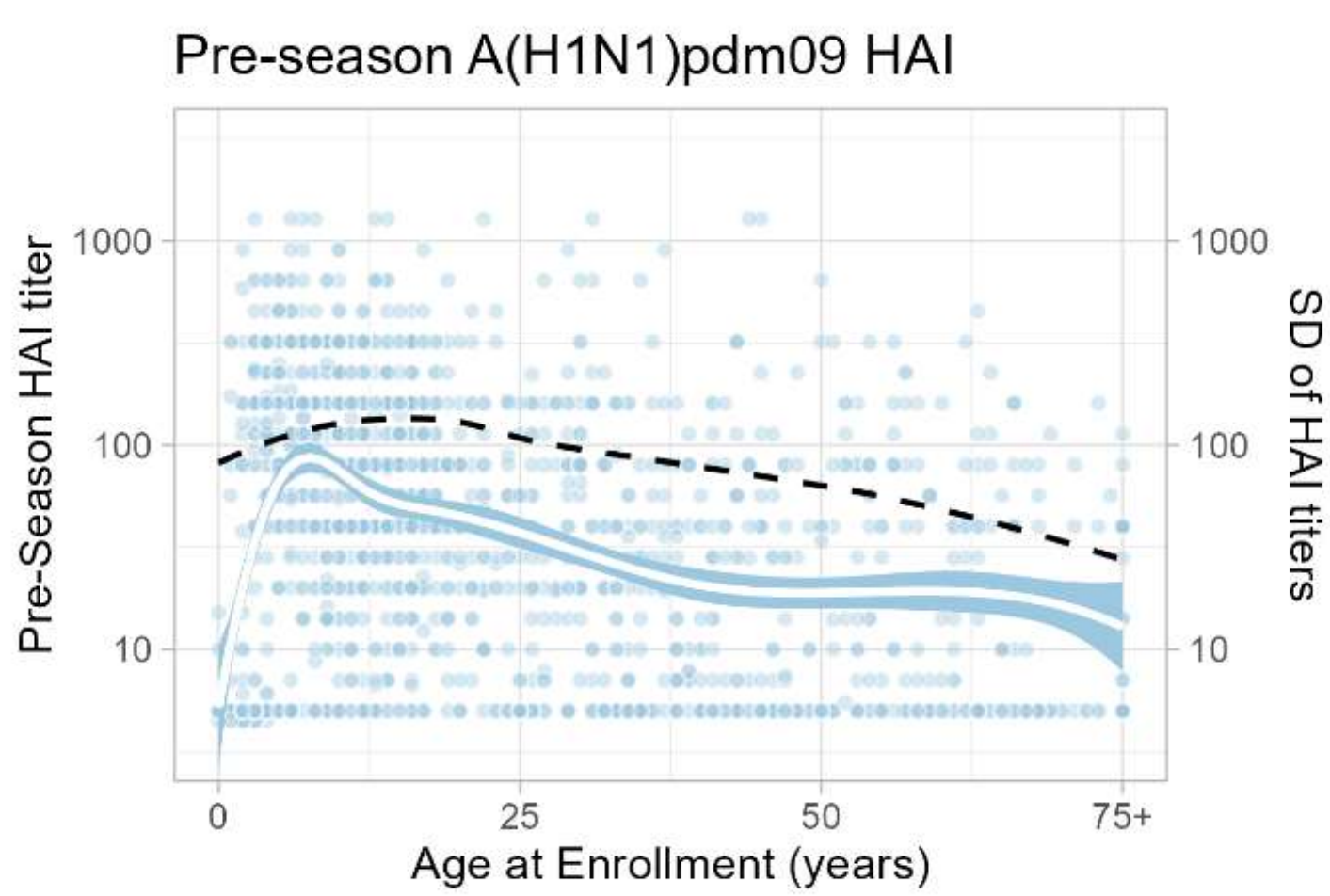

We also added the following to the figure caption (line 482):

> *"... The black dotted line reflects the standard deviation of the titers across ages."*

In the /results we have added the following sentence to reference this pattern in lines 235-239:

> *"In contrast, titer variation started at a high level in early childhood and increased slightly, except for B/Victoria, which declined monotonically, before gradually decreasing with age. This pattern reflects the stochastic nature of infection acquisition and immune boosting in early life, which becomes averaged out as individuals accumulate more exposures over time, as well as intrinsic individual heterogeneity in antibody responses to influenza viruses. "*

Apologies if I missed it, but I do not recall reading about the method used to fit the splines through the data in Fig 1 or Supp Fig 9, what order the fitted polynomials are or any other details?

We are thankful to the reviewer for pointing out the lack of description of the spline fits. We have added this detail to the figure captions for Fig 1 and Supp Fig 9:

> "*… The white solid line is a fitted linear spline fit with B-spline knots at ages 5, 12, 18, and 40 to describe age-dependent patterns …"*

And provided a full description in the Supplementary Methods Section 1 (lines 33-39) as follows:

> *"The age-dependent trend line of HAI titers in Fig 2 and Supplementary Fig 9 were generated in R (version 4.3.1, RStudio 2023.6.2.561) using the base R package splines to create spline-based fits to capture non-linear trends in the data. These curves were constructed with B-splines, with knot placement at ages 5, 12, 18, and 40 to describe age-dependent patterns. We used a cubic spline (degree = 3) because cubic functions are the standard choice in spline modeling; they ensure smooth transitions (continuous first and second derivatives) between segments while maintaining interpretability."*

"For A(H1N1)pdm09 infections, higher pre-season HAI titers correlated with shorter durations of shedding, with a 2.1-day reduction in duration time for those with a titer of at least 40 compared to those with lower titers [95% confidence interval (CI): -3.51 - -0.66 days]. This was not seen for other subtypes." Can the authors hypothesise why this was not the case for other subtypes?

We thank the reviewer for this insightful comment and the opportunity to elaborate on this observation. We first note that viral shedding duration exhibits a strong age gradient, with young children (<5 years) shedding for nearly twice as long as adults (>40 years). A plausible explanation is that cumulative influenza exposures across the life course enhance multiple components of immunity beyond HAI antibodies. These may include non-HAI antibodies targeting neuraminidase (NA) or the HA stalk, influenza-specific memory B and T cells, and binding antibodies that do not inhibit hemagglutination, all of which may contribute to faster viral clearance and shorter shedding duration (lines 399-420).

Mechanistically, HAI antibodies primarily inhibit viral entry by blocking HA–receptor binding, and thus are more directly associated with protection against infection rather than with controlling viral replication once infection occurs. This could explain why pre-season HAI titers were not strong correlates of reduced shedding duration for A(H3N2), B/Victoria, or B/Yamagata. For these subtypes, other immune markers, such as anti-NA antibodies, may play a more dominant role in limiting viral replication and shedding [9].

For A(H1N1)pdm09, the observed association between higher HAI titers and shorter shedding duration may reflect stronger correlation between HAI titers and other mechanistically protective immune responses, such as anti-NA or stalk antibodies. This relationship may differ for A(H3N2), where rapid antigenic evolution of the HA protein can weaken the correlation between anti-HA and anti-NA responses.

In summary, we hypothesize that pre-season HAI antibodies mainly act to prevent infection rather than reduce shedding once infection is established. The exception observed for A(H1N1)pdm09 could therefore indicate a subtype-specific coupling between HAI titers and other immune correlates of viral clearance. While the precise mechanism remains uncertain, we have added the following in the Discussion to reflect this hypothesis lines 396-411:

> *"We found a significant gradient of influenza virus shedding duration with age across all subtypes/lineages in our adjusted models, in which the youngest children shed the longest. We did not find significant impact of pre-season HAI titer level on shedding duration, except for influenza A(H1N1)pdm09. Since children, nevertheless, shed the longest, it is possible that unobserved pre-existing components of adaptive immune memory, such as non-HAI antibodies, could have reduced the shedding duration. These additional immune functions likely operate on viral clearance, as the coefficient of variation was significantly higher for clearance duration than proliferation duration for all but A(H3N2) (Supplementary Table 1). If immune functions beyond HAI antibodies, such as those targeting neuraminidase, the HA stalk, or other influenza-specific cellular responses, play a more critical role in promoting viral clearance for A(H3N2), B/Victoria, and B/Yamagata infections, while pre-existing HAI antibodies are more effective at preventing infection and correlate more closely with viral control for A(H1N1)pdm09, this could explain the subtype-specific effects observed on shedding duration [9]. This interpretation aligns with the hypothesis that pre-season HAI antibodies primarily act to block infection rather than to reduce viral shedding once infection is established, with the exception of A(H1N1)pdm09, where HAI titers may better reflect protective immune responses involved in limiting viral replication."*

"no significant association was found between titers above 40 and viral load reduction for any subtype or lineage". Presumably, this was a surprising result. Do the authors have a hypothesis for why this might have been the case?

We thank the reviewer for this thoughtful comment. From a mechanistic perspective, the absence of a significant association between pre-season HAI titers above 40 and reduced viral load may not be entirely unexpected. Anti-HA antibodies primarily function by blocking viral attachment and entry into host cells, thereby preventing infection. Once infection is established, however, their ability to influence intracellular viral replication is likely limited. Consequently, HAI antibodies are expected to serve as stronger correlates of protection against susceptibility, as we observe in Figure 5, rather than against viral load reduction or shedding dynamics during infection. Adding on to the description given regarding the mechanistic function of HAI antibodies, we also added this description, specific to the lack of association with peak viral load in lines 411-415, immediately following the expansion of Discussion on the last comment:

> *"… Further, this hypothesis could in part explain why there was no significant association between titers above 40 and viral load reduction. But it should be noted that there was also no residual effect of age, and it is also likely that since peak viral load is less directly measurable, it is likely less accurately estimated and further interpretation is limited."*

"For both B/Victoria and B/Yamagata infections, larger household size was associated with a decreased risk of infection acquisition". Is the idea here essentially that larger household sizes actually correspond to a higher absolute infection risk (due to a higher household FOI) – just that the risk is not as high as might be expected based on FOI alone? If so, this should be unpacked more by the authors to avoid readers thinking that the infection risk is lower in larger households! This comment also applies in lines 403-404. If there really is an overall lower infection risk in larger households (even considering both FOI and household size), this should be explained (a dilution effect surely is not a sufficient explanation, as I would expect this to be counteracted by the higher FOI). In addition, the authors could explain how the effect of household size interacts with the household force of infection when they introduce the transmission model in the Supp Material.

We thank the reviewer for this thoughtful comment regarding the interpretation of household size effects and the opportunity to clarify our findings. Specifically, we have clarified this point in the Discussion as follows in lines 429-433:

> *"…Additionally, for B/Yamagata, individuals living in larger households exhibited a lower risk of infection after controlling for household FOI. This finding is consistent with previous influenza studies that have suggested a "dilution effect" of household contact, wherein members of larger households experience a reduced per-contact transmission risk compared with those in smaller households [11]. However, this does not necessarily imply a lower overall household infection risk, as we observed that the cumulative household infection risk (defined as the fraction of household members infected during a given season) varied across households of different sizes (Supplementary Fig 11)."*

We also included the following description the Supplementary Methods section 2 (lines 327-334) to give more context to the reader for our reasons to include household size as a covariate in the household transmission model:

> *"The household force of infection (FOI), defined as the sum of daily viral shedding intensities from infectious household members, was included as a time-varying covariate in the regression model. After accounting for household FOI, we further examined the effect of household size on infection acquisition to adjust for potential reductions in per-person contact intensity in larger households. This so-called "dilution effect," where an increase in household size is associated with a lower person-to-person transmission risk, has been reported in previous household transmission studies[11]."*

We have added the following figure to the Supplementary Materials comparing household size to household attack rate, which shows no clear correlation between the two, indicating that larger households do not inherently have more infections that would contribute to higher household FOIs.

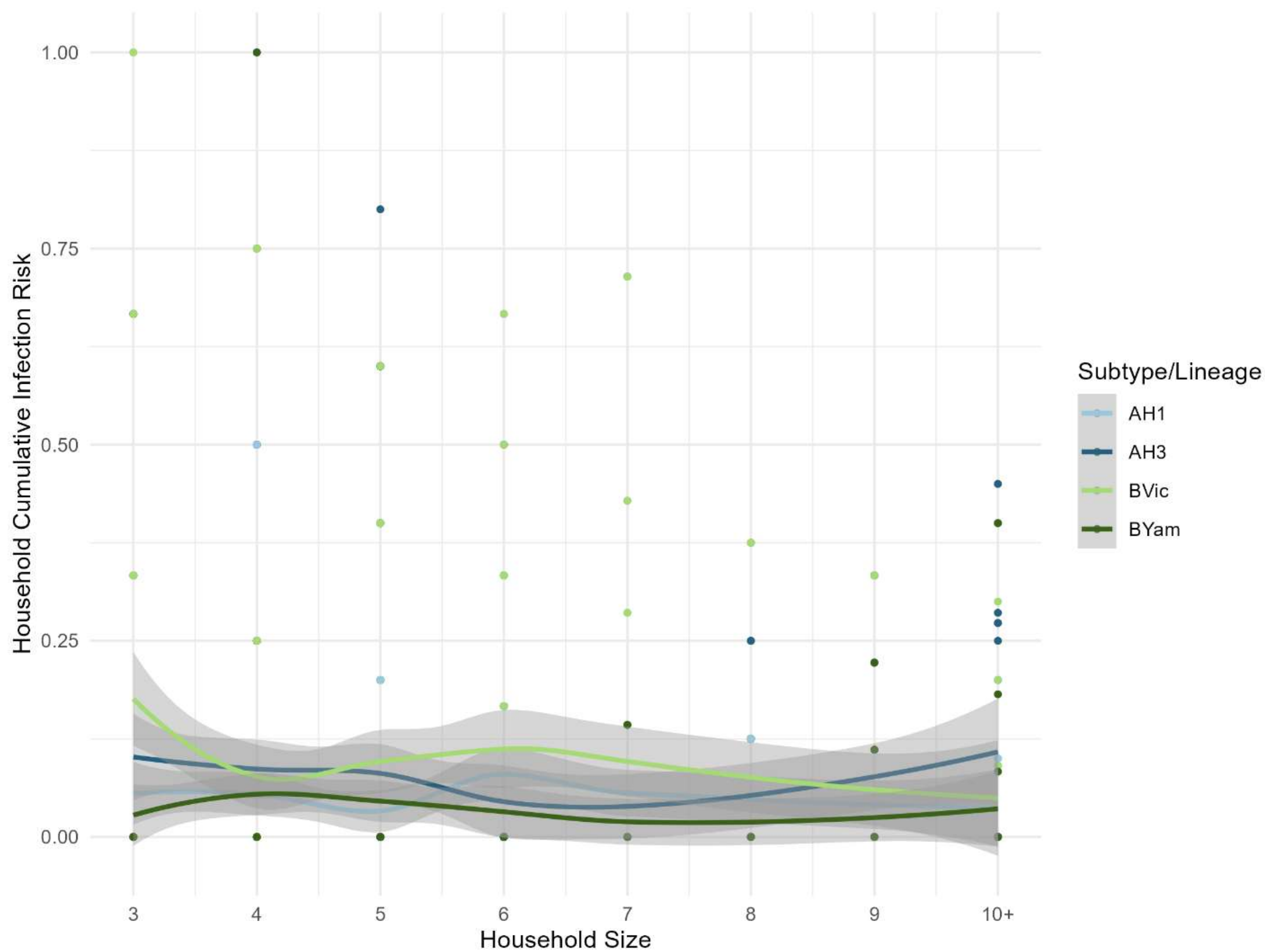

Supplementary Fig. 11 Household cumulative infection risk by household size and influenza virus subtype/lineage *Each point represents the observed cumulative infection risk for a given household size and influenza virus subtype or lineage. The cumulative infection risk was calculated*

as the proportion of individuals experiencing an infection within each household during the observed influenza season. Lines show locally smoothed trends in cumulative infection risk by household size. Household size was treated as a continuous variable from 1 to 10, with all households of size 10 or larger grouped into the "10+" category.

"A 10-unit increase in shedding intensity, as indicated by the Ct value, was associated with approximately a 160% increased risk for A(H1N1)pdm09, 70% for A(H3N2), 90% for B/Victoria, and 50% for B/Yamagata." Can the authors speculate about possible reasons for these values being so different for different subtypes? In general, more discussion about possible explanations for the authors' results would strengthen the manuscript substantially.

We thank the reviewer for this thoughtful comment and for emphasizing the importance of providing mechanistic explanations for our findings. After refining our modeling framework to better account for uncertainty, the estimated effect sizes became more consistent across subtypes/lineages, now showing a 95% increased risk for A(H1N1)pdm09, 65% for A(H3N2), 80% for B/Victoria, and 70% for B/Yamagata, while retaining the same qualitative trends.

While the precise mechanisms underlying these subtype-specific differences remain uncertain, several biological factors could contribute. The Ct value reflects the abundance of viral RNA, which may not directly correspond to the number of infectious viral particles responsible for transmission. The ratio of infectious virions to total RNA copies can vary across subtypes and lineages due to differences in viral replication efficiency, particle stability, or host immune responses. In addition, subtype-specific features, such as variations in receptor binding affinity, replication dynamics in the upper respiratory tract, and the magnitude of mucosal or systemic immune responses, may modulate how viral load translates into infectiousness.

Overall, these observations highlight that while Ct-based measures provide a useful proxy for shedding intensity, they may not fully capture subtype-specific differences in infectious virus output or transmission potential. We have expanded the Discussion to reflect this mechanistic consideration in lines 333-337:

> *"... This observed difference between subtypes and lineages may reflect variation in the infectivity of viral particles, where the same viral load (Ct) corresponds to differing numbers of infectious virions due to subtype-specific differences in replication efficiency, particle stability, and host immune responses not fully captured by controlling for HAI titer and age."*

Lines 359-374. The results relating to the effect of age on infection risk between subtypes are interesting. When the authors claim that if contact rates were the dominant factor then similar patterns would be expected between subtypes, I wondered whether this could also be affected by the lack of power due to the limited number of B/Yamagata infections? When the authors note that age differences between subtypes are indicative of age-specific immune responses, I think it is

worth noting that this could be due to other age-specific factors (not simply average number of contacts) that were also not considered in the transmission model. As one example, if there is substantial heterogeneity in the number of contacts between individuals of the same age, then this could lead to some subgroups of the population appearing to have higher infection risks in under 11s, even if on average in the entire population 12-18 year olds have more contacts.

We appreciate the reviewer's comment. We certainly agree that the lack of significance for B/Yamagata could be due to a lack of power and do suggest this when we first reference the results of the transmission in lines 364-365: "… or a lack of power due to B/Yamagata causing fewer infections than the other subtypes/lineages in our dataset."

B/Yamagata aside, the other three subtypes/lineages still demonstrate differences across the age groups that pique our interest into the role of age in impacting transmission risk, particularly when one compares the two type A subtypes.

We also appreciate the reviewer's suggestion that factors beyond average contact rates, particularly those related to a heterogeneity of contact rates within the same age group or age-dependent behaviors, may contribute to the observed age differences in susceptibility and we should be hesitant to imply that the age patterns are solely a result of age-related immune differences. In response, we have expanded the discussion in the main text in lines 387-389:

> *"… While there could be other age-specific factors such as contact rates and behavioral heterogeneities unaccounted for, our data suggests a potential residual effect of age-specific immune responses."*

As hinted at above, the fitted splines in Fig 1 and the fitted triangles in Fig 2 are not especially convincing. I appreciate that these are average fits, but the data are highly variable, so I am not convinced by attempts to compare relatively small differences in these average values.

For Figure 1, our intention was to highlight the mean population-level trend with age, and the fitted spline serves to capture this average behavior. Similar approaches have been adopted in previous studies with comparable levels of heterogeneity to characterize mean immune responses across ages [5]. We agree, however, that quantifying variability is equally important. As mentioned earlier, we now include dashed lines representing the standard deviation of antibody titers across ages to better convey the degree of variation around the mean trend.

For Figure 2, we acknowledge that the data points are inherently stochastic, as each infection episode varies in duration and peak viral load (minimum Ct value). The fitted triangles are intended to illustrate the average viral shedding trajectories estimated by our model, while the box plots summarize the distributions of estimated peak viral load and duration across individual infections. The fitted triangles were informed not by the raw Ct values as indicated by the points but instead taken from the mean estimates. We acknowledge that this figure was hard to interpret and to avoid this confusion further, we removed the raw Ct values and presented only the fitted triangles.

Finally, while we recognize the large within-group variability, we believe that comparing mean values across groups remains informative. As a purely illustrative example, one can meaningfully compare the average height of children aged 10–12 versus those aged 13–15 to understand growth patterns with age, even though individual heights within each group are variable and overlapping across group. In a similar way, our comparisons of average titer changes and shedding characteristics capture systematic differences across age or subtype groups despite individual-level heterogeneity.

We also want to highlight that this large within-group variability is not only expected but also key to understanding a later hypothesis regarding the large variation in clearance duration across individuals relative to proliferation duration gives hint to the role of unmeasured immune factors in facilitating clearance of infection. In the Results section, while we do list the subtype/lineage differences in duration, the only result we claim to be statistically significant is the following in lines 252-254:

> *"… The coefficients of variation were higher for the clearance duration than proliferation duration, significantly so for all but A(H3N2) by asymptomatic testing for equality of coefficients of variation (p-value < 0.05)."*

In Fig 2A, lots of dots appear that are not on the graphs! For example, there are dots off the left of the bottom left panel, as well as in between the panels.

We thank the reviewer for pointing this out. In response to a separate comment, we decided to remove the dots for the raw Ct data altogether.

Supp Material, line 79. I think there is an error in the definition of Delta Ct (or Ct??) between times tp and tp+omega_c? Given that Ct is not constant there, then as a minimum there should be a dependence on t? These equations (and all other methodological details) need careful checking.

We thank the reviewer for catching this. We have fixed the typo and carefully rechecked all related equations and methodological details for consistency throughout the manuscript.

Minor comments:

Supp Material, lines 95-101. What are the numbers in square brackets? The notation here should be explained.

We appreciate the reviewer for pointing out the lack of clarity in our modeling description. The numbers represent the range in which we truncate the exploration of the model for each given parameter, cutting off the priors at extreme values to improve efficiency of the fitting. In making substantial edits to our Supplementary Materials, we removed these numbers from the brackets next to the priors as used before and instead described them within lines 107-110 for better clarity:

"… To improve computational efficiency, constrained the parameters to biologically plausible upper bounds: $\omega_p < 15\ days$, $\omega_c < 30\ days$, and $\Delta Ct_{max} < 37$, as values beyond these thresholds are considered implausible for influenza viral kinetics."

Did the authors rigorously account for censoring (i.e. false negative test results) when fitting the shedding model?

We thank the reviewer for raising this important point. We addressed the issue of censoring through the Bayesian probabilistic framework. Specifically, our shedding model was implemented within a Bayesian framework that fits the underlying shedding kinetics most consistent with the observed longitudinal Ct values at discrete time points. The probabilistic nature of the model allows multiple plausible shedding trajectories that can equally explain the observed data. In cases of false negatives or missing samples, this uncertainty is naturally reflected in a broader posterior distribution of the shedding parameters.

In the revised manuscript, we also explicitly account for this uncertainty in the household transmission model used to evaluate the shedding kinetics and infection risk factors (Fig. 3 and 5). Specifically, we resample each individual's posterior shedding trajectory 100 times, refit the shedding duration, shedding peak, and household transmission regression models for each realization, and pool the resulting estimates. This approach ensures that uncertainty in viral shedding kinetics is fully propagated into the estimation of infection risk. We noted this approach in the Supplementary Materials when describing each regression and clarified this in the revised main text Methods section as follows (lines 165-169):

"…In all following regression analysis, to account for uncertainty in the shedding estimates due to potential censoring or missing observations, we resampled from the posterior; each regression was run 100 times, drawing one random posterior estimate per individual from the shedding model outputs, and the resulting regression estimates were pooled to obtain the final reported values."

Supp Material, line 213. The formatting of this equation is not good, with squares appearing (empty equation fields) – this should be fixed.

We thank the reviewer for pointing this out and have fixed the issue in the revised version.

Supp Material, lines 219-230. I am not sure the stated formulation makes sense for the non-binary categorical variables. Also, is the household size effect assumed to be monotonic?

We thank the reviewer for this helpful comment. We have clarified the notation in the equations in the Supplementary Methods to indicate that categorical variables, in bold text and noted in the variable description, are represented as vectors (with one coefficient per non-reference category), while continuous variables are modeled with a single coefficient, assuming a linear relationship

with the outcome. Regarding household size, this variable was modeled as continuous; therefore, its effect is assumed to be monotonic.

Supp Material, lines 143-144. "Year was incorporated as a random effect for all except B/Yamagata". It might be worth reminding the reader here that B/Yamagata only circulated in one year (this is stated in the main text, but I forgot this by the time I was reading the Supp Material). Also, how was year incorporated as a random effect in the model – can the authors explain the method in the Supp Material?

We agree with the reviewer that it is important to remind the reader of this fact within the supplementary materials and that we failed to explain how the random effect is included in the model. We replaced the current description with a more detailed one Supplementary Materials section in lines 216-224 as follows:

> *" … To account for potential batch effects across study years, we included study year as a random effect in the regression model. This approach allows the model to estimate a separate intercept for each year while simultaneously estimating the variance of these intercepts across years. In doing so, the model captures unobserved year-to-year variability without assuming that such differences systematically influence the relationship being tested, for example due to differences in measurement or sample collection. For B/Yamagata, which circulated in only a single study year, inclusion of a random effect for year was not possible; therefore, a standard multivariable linear regression model was used instead."*

When the authors introduce the transmission model in the Supplementary Material, they include terms for community transmission and household transmission. Doesn't this pre-suppose that these two locations are the main sources of transmission? I do not know much about the locations of the PHIRST study, but certainly in some countries school-based transmission is a key driver. Some rationale for focussing solely on community and household transmission, rather than e.g. school (or any other specific location) transmission, would be beneficial.

We thank the reviewer for this helpful comment. We would like to clarify that the "community force of infection (FOI)" in our model is defined broadly to include all exposures occurring outside of the household, such as in schools, workplaces, or other community settings. Referring to these collectively as "community FOI" is standard practice in household cohort studies. Our goal in including this term was not to identify specific transmission settings, but rather to control for all non-household sources of exposure to more robustly evaluate the effects of naturally acquired immunity, age, and other characteristics [12].

To clarify this in the text, we have revised lines 348-351 to read:

> *"Similarly, increased prevalence in the general community, estimated from the overall cohort population, accounting for any exposure outside of the household such as in schools*

or workplaces, was also strongly correlated with an increased risk of infection acquisition."

Supp Material, lines 231-233. "Observations were created for each day in the influenza season, and we assigned a binary dependent variable indicating a 0 when no infection has occurred and a 1 on the estimated day of infection acquisition". Was this done independently of the transmission model (e.g. based on individuals' measured viral loads), or was the day of infection acquisition estimated using the transmission model? I suspect it is the former, but this should be explained more clearly.

We agree that our description here leaves room for confusion regarding the model. The assignment of the infection outcome at daily resolution was solely based on the viral shedding model, independent of the transmission model. To clarify this in the Supplementary Materials we revised lines 316-321 as follows:

> *"Observations were created for each day in the influenza season, with a binary dependent variable indicating infection status. The estimated day of infection acquisition was derived from the shedding model, using the estimated duration of shedding and the day of peak infection: we subtracted the estimated proliferation duration from the peak day to obtain the first day of shedding, and, following prior literature, assumed infection acquisition occurred one day before shedding onset [13]."*

Line 118. "Blood serum samples were collected at least once prior to the influenza season". I think this should say "each influenza season"?

Typo fixed.

Line 163-164. "The onset of infection was inferred to be one day prior to the first day of shedding". First of all, by saying "inferred", I assumed that this was a result of the authors' analysis – whereas in fact I think they took this value from the literature. Second, might this be expected to vary between individuals?

We appreciate the reviewer for highlighting this unclear language. This value was taken from challenge studies that uniformly suggest that individuals begin shedding within the first 24 hours after inoculation [13,14]. It is possible that this could vary between individuals, particularly in considering that higher HAI antibody titers would act to prevent infection and might delay onset and that natural infection is not produced with a uniform exposure level as in challenge studies. But since our transmission regression based on the sampling scheme is only robust enough to include an onset in the unit of days (as opposed to hours), and the strong evidence from previous studies that shedding begins within a day of inoculation, we considered this approximation appropriate. We have changed the language to better reflect that this choice was informed by literature as follows in lines 163-165:

"… The onset of infection was set as one day prior to the first day of shedding based on kinetics observed in challenge studies [13,14]"

Lines 241-243. "The variance of the clearance…". Should this be mean rather than variance?

We thank the reviewer for pointing this out; we in fact meant to discuss the coefficient of variation and made the following edits in line 252 to make this clearer:

"… The coefficients of variation were higher for the clearance duration …"

Line 295. I think ">1:40" is a typo and should just be ">40".

Typo fixed.

Lines 351-352. "however modeling titers on a continuous scale supports a relationship (Supplementary Fig 8)." I would consider talking the reader through Supp Fig 8 in a bit more detail to help explain this result; I found this confusing.

We appreciate the reviewer pointing out the lack of context for Supplementary Figure 8. We revised lines 360-362 as follows:

"However, running the same transmission model, with the only difference being to treat titers as a continuous variable rather than ⩾40 or <40 categorical, higher B/Yamagata titers did associate with a reduce infection risk (Supplementary Fig 8)."

Table 1. Please specify what the numbers in brackets represent. Is the value for males in 2016 deliberately obscured?

Specification now given in figure caption as follows and document issue that caused the obscured value was removed (lines 470-472):

"*Brackets indicate percentage relative to the given N for the year and category (Individual Characteristic/Household Characteristic/Observed Infection Episodes)."*

In the figure captions, the authors claim that asterisks denote significance levels. It is slightly pedantic (sorry), but aren't the asterisks actually indicative of p-values rather than significance values? (i.e. *** indicates a p-value less than the significance level of 0.001 etc). The wording here should perhaps be made more technically correct (although I appreciate that it is already clear what the authors mean).

We thank the reviewer for this comment and agree that it best to denote p-value instead of significance, so as to not confuse interpretation of the figures. We edited the captions figures to fix this (Figures 3 and 5, Supplementary Figures 7 and 8).

Fig 3. Why is there no uncertainty in the shedding duration and minimum Ct for the reference groups? If the reference values are the same in each category, should e.g. the shedding durations

in different age groups be interpreted as being age-specific estimates for HIV-negative females with pre-season HAI titre <40?

We thank the reviewer for this insightful comment. Our analysis uses a multivariable linear regression framework in which one category from each variable serves as the reference group. By definition, this reference category provides the baseline against which all other categories are compared and therefore does not have its own confidence interval. The displayed estimates and uncertainty intervals for other categories represent differences relative to this baseline (e.g., relative to >40-year-old, HIV-negative females with pre-season HAI titers <40). The reference group is thus a statistical construct used to anchor comparisons and does not imply that the results are specific to that subgroup; the modeled associations apply across the full study population.

Fig 4. Is this really a “schematic”? If I understand the figure correctly, it is actually a visualisation of results from the fitted model, so I am not sure that "schematic" is the correct word to use? (I do not have a strong feeling about this though, so please feel free to ignore!)

We agree with the reviewer that “schematic” was a misleading choice for the title, given the use of model results in the figure. We revised the title to the following (line 505):

> “*Visualization of key variables of the influenza household transmission model.”*

Fig 5. Should the caption and/or figure specify that hazard ratios for community FOI are (I think) with respect to a 1% change in prevalence?

We thank the reviewer for pointing out this opportunity to improve figure clarity. To better clarify community FOI and household FOI, we removed the small sub-text from the figure under Household FOI and inserted the following into the figure caption (lines 516-519):

> “*Community FOI is indicated with respect to a 0.01 increase in absolute community prevalence (defined as the total number of infection episodes in the cohort at a given time t / cohort population). Household FOI is indicated with respect to a step by 10 Ct units within the household.”*

Reviewer #2 (Remarks to the Author):

This is a household cohort study conducted in rural and urban communities in South Africa across 3 influenza seasons to inform influenza transmission models. 327 households (1684 individuals) were enrolled with follow-up, averaging 110 new households enrolled each year. Serum was collected prior to influenza season to measure antibody titers against circulating strains (HAI)/prior immunity. Respiratory samples were collected twice weekly, regardless of symptoms, to identify influenza infections (rRT-PCR). The authors leveraged this PHIRST study and comprehensive data collected to examine the role of prior immunity, age, and exposure intensity on risk of influenza infection/acquisition in an unvaccinated population in low- and middle-income country.

This is nicely presented, clearly written manuscript. I am unable to comment on the specifics of the modeling (calibration, etc), but the approach, assumptions, and model inputs are appropriate and clearly described. The major strength and contribution of this paper is that the transmission models are subtype/lineage-specific and the models include and assess measures for prior immunity. Preseason HAI titers were correlated with shedding duration only for A(H1N1)pdm09; preseason HAI titers ≥1:40 were protective against infection (except against for B/Yamagata). Higher viral shedding was associated with higher risk of infection. Younger age was consistently (and independently) associated with longer shedding duration, higher viral loads, and higher risk of infection. The variability in the results highlights the challenges with influenza and the need to examine influenza by subtype/lineage whenever possible. The authors interpretation of the data and conclusions are valid. The presentation of data, especially the forest plots are very nice. I do not have any concerns with the methodology, data analysis, interpretation, or conclusions. This work is remarkable and adds to our understanding of influenza transmission in an unvaccinated population in rural and urban communities in South Africa.

We thank the reviewer's positive assessment of our manuscript and for highlighting the uniqueness and importance of the PHIRST cohort study design and data set. We also appreciate the commendation of our modeling scheme and data presentation. We have addressed the reviewer's question below and edited the manuscript as suggested.

minor comments

- The authors mention the PHIRST study had a high prevalence of asymptomatic infections, did the authors examine or are the authors able to examine the role of asymptomatic infections in shaping household transmission dynamics?

We thank the reviewer for this important suggestion. We did not explicitly examine the role of asymptomatic infections in shaping household transmission dynamics in the present analysis. However, we are currently conducting a multi-pathogen analysis—including influenza, RSV, and SARS-CoV-2—to systematically assess the contribution of asymptomatic and pre-symptomatic infections to household transmission across these major respiratory pathogens. We believe this broader comparative framework will provide a more comprehensive understanding of the epidemiological importance of asymptomatic transmission, a key feature of respiratory viruses.

- Line 295 – typo? I think “>1:40” should be “≥40”

Typo fixed.

Reviewer #3 (Remarks to the Author):

In this study, the authors presented their quantitative analysis that estimated the household transmission risk of influenza viruses in two cohorts of participants in South Africa. The authors

provided a nice summary of the estimated viral shedding kinetics (Fig. 2) and predictors of infection risk (Fig. 3). They developed a detailed household transmission model to assess how influenza virus exposure intensity, age, and serum HAI titers influence the risk of infection acquisition (Figs. 4 and 5).

I appreciate the great efforts of the authors in organizing the intensive samplings of nasopharyngeal swabs in the PHIRST cohorts. This study design is unique and not easy in implementation. The estimates of infection risk predictors and the relationship between pre-season serum titer and correlates of protection against infection are useful to improve the understanding of household transmission of influenza in South Africa. The authors performed the analysis for all four influenza subtypes/lineages.

We thank the reviewer for the positive remarks on the PHIRST cohort study design and our manuscript. In the revised version, we have carefully addressed the reviewer's comments and provided point-by-point responses below.

There are a few points not very clear in the current manuscript:

1. The influence of school schedules and holiday seasons could be discussed. The authors found that children had higher risk of infection. It looks unclear whether children cases were clustered in time or randomly distributed over the study period.

We appreciate the reviewer's suggestion to clarify the potential influence of school schedules and holiday seasons. To address this, we added a new supplementary figure (Fig 10 – included in rebuttal letter above) showing the community FOI for each subtype/lineage across the three study years, with shaded regions indicating school terms and separate panels illustrating infection prevalence among school-aged children (5–18 years) and young children/adults. The time series demonstrates relatively synchronized infection dynamics between school aged children and others, suggesting that differences in infection risk are unlikely to be driven primarily by school schedules or holidays. While influenza seasonality does coincide with school terms, the overlap appears coincidental rather than causal, and the observed higher infection risk among children is likely to reflect underlying biological and immunological factors rather than timing of school attendance.

2. I don't quite understand the influence of HIV status on the risk of influenza infection. It would be perfect if the authors could add some biological interpretation on this.

We appreciate the reviewer's comment regarding the role of HIV status in infection risk. The inclusion of HIV status was motivated by the design of the PHIRST cohort, which was established in settings with a high HIV prevalence to assess its potential influence on influenza transmission dynamics and disease burden [2]. However, most participants living with HIV in our cohort were on antiretroviral therapy with well-managed disease, resulting in few individuals with severe immunosuppression (CD4 <200) and, consequently, limited power to detect an effect. Despite this, we retained HIV status as a covariate given its epidemiological relevance in the study context. We have edited lines 175-177 as follows to make this clearer in the present manuscript:

"HIV status was included as a covariate given the high prevalence of participants living with HIV (PLWH) in the study population and its potential relevance to influenza transmission dynamics."

We also added the caveat of the limited population size of immunocompromised individuals into the discussion in lines 424-426:

"While there was a relatively large portion of people living with HIV included in our analysis, most participants living with HIV were on effective treatment, limiting the power of analysis on immunocompromised individuals."

3. In line 286, the authors estimated that "a one percent increase in influenza prevalence in the population was associated with a 30-100% increase in risk". This estimate may suggest a strong influence of community FOI on the household risk of infection. This looks a bit overestimated.

We thank the reviewer for pointing out this important clarification. We want to clarify that the 1% increase refers to an absolute rather than a relative increase in community prevalence. To provide better context, community influenza prevalence at the seasonal peak was only around 5–6%, so a 1% absolute increase represents a substantial rise in community burden. To make this easier to interpret, we changed all uses of "1% increase" to 0.01 increase. We have also added Supplementary Figure 10 to illustrate these dynamics and revised the Results text in lines 286-292 accordingly:

"The community FOI (proxied by influenza infection prevalence in the cohort) was significantly associated with an increase in risk of infection acquisition for all four subtypes/lineages, such that a 0.01 increase in influenza prevalence in the population, for which the peak prevalence was between 5-6%, was associated with a 70-100% increase in risk (A(H1N1) pdm09 1.89 HR, 95% CI: 1.68-2.13; A(H3N2) 1.69 HR, 95% CI: 1.56-1.82; B/Victoria 2.01 HR, 95% CI: 1.83-2.20; B/Yamagata 1.95 HR, 95% CI: 1.74-2.19; supplemental figure 10."

4. In the paragraph starting with line 304, the authors identified the influence of household size on influenza B virus but not influenza A virus. The underlying mechanism is not very clear to make this claim.

We thank the reviewer for this helpful comment. We agree that the mechanism underlying the observed difference between influenza B and A was not clearly explained in the earlier version. In the updated analysis, the association between household size and infection risk was observed only for B/Yamagata. While previous studies of type B influenza have suggested a potential "dilution effect," whereby individuals in larger households experience lower per-person exposure due to reduced contact intensity, we consider the B/Yamagata-specific result in our data more likely to reflect stochastic variation given the small sample size for this lineage. We have clarified this interpretation in the revised main text (429-433):

"Additionally, individuals in larger households exhibited a decreased risk of acquiring B/Yamagata. While previous studies of type B influenza have suggested a role of a dilution effect, in which each household member has less extensive exposure to the virus on a per-contact basis, this result solely for B/Yamagata is more likely a result of a stochastic effect of the small sample size [10,11]*."*

5. In line 160-161, "at a population mean and individual (per infection episode) level." This statement looks confusing.

We thank the reviewer for pointing out this unclear phrasing. The lines 151-161 have been edited in the main text as follows:

"To calibrate the model, we implemented a Bayesian hierarchical framework using the Hamiltonian Monte Carlo (HMC) algorithm to generate posterior draws for parameter inference. Each draw estimates the value and timing of the minimum Ct (a proxy for peak viral load), as well as the durations of the proliferation and clearance phases, both at the population mean for each subtype or lineage and at the individual (per-infection episode) level."

Reviewer #4 (Remarks to the Author):

I co-reviewed this manuscript with one of the reviewers who provided the listed reports. This is part of the Nature Communications initiative to facilitate training in peer review and to provide appropriate recognition for Early Career Researchers who co-review manuscripts.

We appreciate your support of the review of this manuscript.

Citations

1. Sun, K. *et al.* SARS-CoV-2 transmission, persistence of immunity, and estimates of Omicron's impact in South African population cohorts. *Sci. Transl. Med.* **14**, eabo7081 (2022).
2. Cohen, C. *et al.* Asymptomatic transmission and high community burden of seasonal influenza in an urban and a rural community in South Africa, 2017–18 (PHIRST): a population cohort study. *Lancet Glob. Health* **9**, e863–e874 (2021).
3. Hayden, F. G. *et al.* Use of the oral neuraminidase inhibitor oseltamivir in experimental human influenza: randomized controlled trials for prevention and treatment. *JAMA* **282**, 1240–1246 (1999).
4. Ip, D. K. M. *et al.* The Dynamic Relationship Between Clinical Symptomatology and Viral Shedding in Naturally Acquired Seasonal and Pandemic Influenza Virus Infections. *Clin. Infect. Dis. Off. Publ. Infect. Dis. Soc. Am.* **62**, 431–437 (2016).
5. Yang, B. *et al.* Life course exposures continually shape antibody profiles and risk of seroconversion to influenza. *PLoS Pathog.* **16**, e1008635 (2020).
6. Lessler, J. *et al.* Evidence for Antigenic Seniority in Influenza A (H3N2) Antibody Responses in Southern China. *PLOS Pathog.* **8**, e1002802 (2012).
7. Ranjeva, S. *et al.* Age-specific differences in the dynamics of protective immunity to influenza. *Nat. Commun.* **10**, 1660 (2019).
8. Suntronwong, N. *et al.* Prevalence of antibodies against seasonal influenza A and B viruses among older adults in rural Thailand: A cross-sectional study. *PLOS ONE* **16**, e0256475 (2021).
9. Hoy, G. *et al.* Anti-Neuraminidase Antibodies Reduce the Susceptibility to and Infectivity of Influenza A/H3N2 Virus. 2024.06.14.24308936 Preprint at https://doi.org/10.1101/2024.06.14.24308936 (2024).
10. Gordon, A. *et al.* Influenza Transmission Dynamics in Urban Households, Managua, Nicaragua, 2012–2014. *Emerg. Infect. Dis.* **24**, (2018).
11. Cauchemez, S. *et al.* Determinants of Influenza Transmission in South East Asia: Insights from a Household Cohort Study in Vietnam. *PLoS Pathog.* **10**, e1004310 (2014).
12. Longini, I. M., Koopman, J. S., Monto, A. S. & Fox, J. P. Estimating household and community transmission parameters for influenza. *Am. J. Epidemiol.* **115**, 736–751 (1982).
13. Carrat, F. *et al.* Time lines of infection and disease in human influenza: a review of volunteer challenge studies. *Am. J. Epidemiol.* **167**, 775–785 (2008).
14. Memoli, M. J. *et al.* Validation of the wild-type influenza A human challenge model H1N1pdMIST: an A(H1N1)pdm09 dose-finding investigational new drug study. *Clin. Infect. Dis. Off. Publ. Infect. Dis. Soc. Am.* **60**, 693–702 (2015).

Reviewer #1 (Remarks to the Author):

Thanks to the authors for their responses to our initial set of comments. In general, the authors have provided comprehensive answers to our questions – thank you for that. However, there are still some aspects that we are not convinced about. We therefore think that more work should be done to improve this manuscript before it is considered for publication.

We thank the reviewer for their thoughtful follow-up comments and for the overall positive assessment of our responses to the initial set of comments. We greatly appreciate the care taken to provide detailed feedback that has helped us to further clarify and strengthen our manuscript. We have addressed the reviewer's remaining concerns in the revised manuscript and provide detailed, point-by-point responses below.

Major comments:

1. The authors use Supplementary Figure 11 to conclude that "the cumulative household infection risk (defined as the fraction of household members infected during a given season) varied across households of different sizes (Supplementary Fig 11)." Looking at the trend lines in that figure, it looks to us like the infection risk does not vary much with household size. We therefore do not agree with the authors' interpretation.

We thank the reviewer for this comment and agree. Upon reexamination, we found that our original wording was incorrect; our intention was to convey that there was no clear increase or any trend in infection risk with increasing household size. We have corrected the text as follows in lines 423-426:

> *"However, this does not necessarily imply a lower overall household infection risk, as we observed that cumulative household infection risk (defined as the fraction of household members infected during a given season) does not vary across households of different sizes (Supplementary Fig 11)."*

2. Supp Fig 11. The authors argue that fitting trend lines through scatter plot data is commonplace. We agree, and do not generally have an issue with this. However, looking at the dots here, the data points are really scattered – so do the authors really believe that that the observed trends can be trusted?

3. Supp Fig 11 (again!). The caption seems to suggest that there ought to be 4 points for each x value from 3 to 9, but some x values have 5 points ("Each point represents the observed cumulative infection risk for a given household size and influenza virus subtype or lineage."; this suggests to us that at each household size there ought to be one point per subtype?) All the y-values seem to be multiples of 1/(household size), so we wonder if a dot is actually being plotted for every individual household (with lots of overlapping dots, so that there are lots of data points there that cannot be seen). If so, the authors should find a way to visualise the data more clearly (maybe with dots with areas corresponding to the number of points there), and the caption should be corrected.

We thank the reviewer for these comments and agree that the original presentation of Supplementary Figure 11 was confusing. In the original figure, each point represented an individual household–subtype observation, rather than a single summary value per household size and subtype. Because cumulative infection risk is defined as the number of infected household members divided by household size, many households shared identical values (multiples of 1/household size), resulting in substantial overlap that was not readily apparent. We agree that this made it difficult to visually assess the distribution of the data.

To address both concerns, we have revised Supplementary Figure 11 to now represent the number of households at each cumulative infection risk value, using dots whose size reflect the number of overlapping observations, rather than plotting each household individually. In addition, we have removed the smoothed trend lines and instead marked the mean cumulative infection risk for each household size with an "X." The figure caption has been updated accordingly to accurately describe the revised visualization. We believe that these changes make the figure more transparent and better support our intended conclusion that there is no clear or consistent relationship between household size and cumulative infection risk.

4. Fig 1. Again, the authors argue that fitting splines through scatter plot data is commonplace. However, when the data are as variable as they appear in Fig 1, we find it hard to be convinced that any trend lines are genuinely trends as opposed to simply being reflective of smoothed lines through a quantity that is highly variable. We think that it might be beneficial for the authors to also visualise a summarised version of the data without smoothing, to assess how reasonable the observed trends are. For example, the authors could plot the average of the data points within age bands to demonstrate that this shows a similar trend (without applying any smoothing). If this displays a similar trend, this would make the inferred trends more convincing; if not, then the trends should be removed.

We thank the reviewer for raising these important points. We have reworked the figure to provide summary statistics without a trend line (new Fig 1, see below) and moved the initial figure to supplement (Supplementary Figure 12). The new figure presents violin plots of the data stratified into age bands, showing both the full distribution of observations within each band as well as the corresponding group medians. This approach allows the underlying structure of the data to be assessed without applying any smoothing. These violin plots demonstrate that the overall age-related patterns observed within the binned data are consistent with the smoothed trends shown in Supplementary Fig. 12 and described in the main text, providing reassurance that the fitted splines are not artefacts of smoothing highly variable data but instead reflect genuine structure present in the observations.

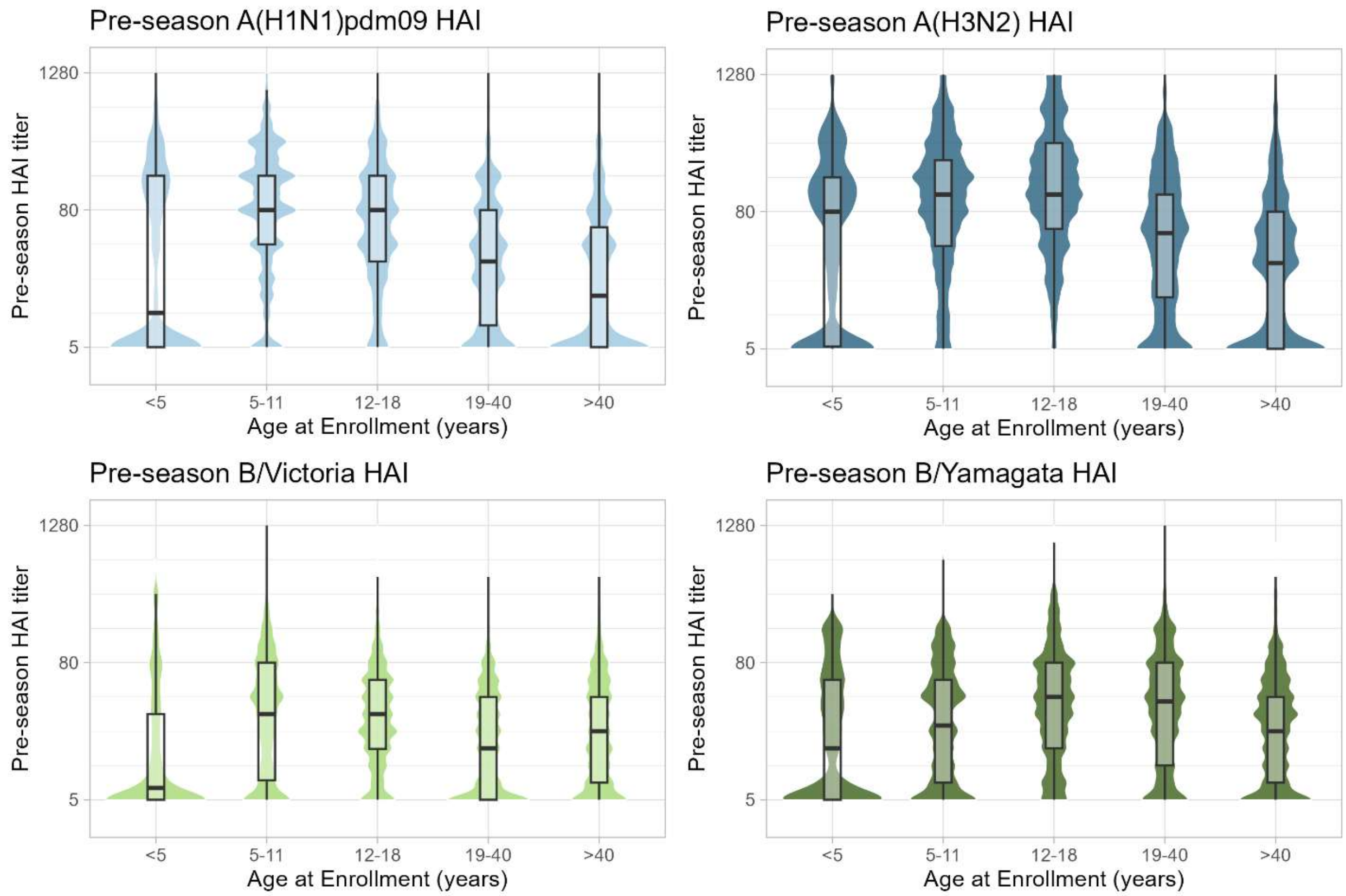

In particular, we believe that the new Figure 1 provides important biological context for the observed trend by more clearly illustrating the distribution of antibody titers across age groups. Notably, the youngest age group (<5 years) exhibits a distinct bimodal distribution of titers. This bimodality reflects the stochastic nature of early-life influenza exposure, whereby a substantial proportion of young children remain immunologically naïve, while others have already been exposed to influenza early in life and subsequently seroconvert. In contrast, middle age groups display more unimodal distributions with substantial variability, consistent with the accumulation of repeated exposures over the life course. In the oldest age group, titers decline again with noticeable seroreversion, likely reflecting immune aging and/or immune imprinting effects. We have therefore retained this figure in the Supplementary Materials and now explicitly reference it in the Results section at the point where we discuss the stochastic nature of infection acquisition (lines 222-236):

> *"We examined the distribution of pre-season hemagglutination inhibition (HAI) titers across age groups (Fig. 1). The youngest group (<5 years) displays a distinctly bimodal titer distribution, consistent with the stochastic nature of early-life influenza exposure: many children remain immunologically naïve, while others have already experienced infection and seroconverted. In contrast, individuals aged 12–18 years exhibit a more unimodal, but still broadly distributed, titer profile, reflecting the accumulation of heterogeneous exposure histories over time. Among older adults, the titer distribution reverts to a bimodal pattern, with evidence of seroreversion, potentially indicative of waning immunity due to aging and/or the long-term effects of immune imprinting.*

Supplementary Fig. 12 illustrates the age-dependent trajectory of average pre-season hemagglutination inhibition (HAI) titers. For both influenza A subtypes, titers increase steeply between ages 0 and 10, whereas influenza B lineages show a more gradual rise over a broader age range, likely reflecting less frequent immune boosting due to lower infection rates, as well as the poorer persistence of antibodies against influenza B viruses compared with those against influenza A. Thereafter, titers for all subtypes and lineages decline progressively with advancing age and eventually plateau in older adulthood at levels of approximately 20–30."

We hope that the inclusion of this additional figure and the accompanying discussion address the reviewer's concerns and strengthen confidence in the trends originally described.

5. In our previous review, we asked about the previous claim that "A 10-unit increase in shedding intensity, as indicated by the Ct value, was associated with approximately a 160% increased risk for A(H1N1)pdm09, 70% for A(H3N2), 90% for B/Victoria, and 50% for B/Yamagata." In the authors' response, they note that in their revised analysis there is more consistency between subtypes. However, they go on to explain potential mechanisms underlying the subtype specific differences. In the manuscript, however, it seems that the CIs now overlap substantially between subtypes; so we would simply argue that the values were similar for all subtypes rather than trying the explain why they are different (since the authors' Bayesian analysis does not support that a difference exists).

We agree with the reviewer's assessment and have therefore removed this hypothesis from the manuscript.

6. While the authors have improved their explanation of the modelling approach taken, we are still not completely convinced about all of the aspects. For example, in Supplementary Figure 1 (and in the text), t is described as calendar time. But doesn't t instead represent time within an individual's infection (rather than overall calendar time)? If not, then when t=0, which may be months before an individual becomes infected, then following the authors' explanation in the Supplementary Information literally then their viral load is drawn from max(0, Normal(0,sigma)) – which seems a little odd?

We appreciate the reviewer's careful reading and the opportunity to clarify this point. In the updated description, t represents calendar time only, as indicated in Supplementary Fig 1 and throughout the text. It does not represent time relative to an individual's infection. The parameter t_p denotes the calendar time at which an individual's peak viral load is reached; it is not centered at zero but instead corresponds to the estimated calendar date of peak shedding. The parameters ω_p and ω_c represent the durations of the proliferation and clearance phases, respectively, and are both expressed in units of days.

It is worth clarifying the behavior of the model for calendar times far preceding the infection episode, when the true viral load is expected to be zero. In this regime, the term max(0, Normal(0, sigma)) represents observational error and accommodates the possibility of spurious low-level signals. Specifically, when the observed viral load lies just above the detection threshold, there is a non-negligible probability that the signal does not reflect true PCR positivity

but instead arises from measurement noise, limited PCR precision, or occasional sample contamination. Our inference suggests sigma ~ 1, which is consistent with the expected magnitude of PCR variability close to the assay's detection limit.

7. We may be misunderstanding, but the authors seem to use the household data to determine the community prevalence in their model. Then, they analyse the interplay between community prevalence and household exposure, concluding that the infection risk in households was high when the community prevalence was high. This conclusion is in fact simply the assumption that the authors made when they chose to set the community prevalence based on the household cohort data? This seems to be circular reasoning to us.

We thank the reviewer for raising this important point. The households included in our study constitute a random sample drawn from the surrounding community, and community prevalence is estimated by aggregating infection status across all individuals in the cohort for a given site and year. As such, the cohort provides a reasonable, and, for this study, the most direct, estimate of community prevalence. Unfortunately, no other surveillance data set exists to inform community prevalence for the studied locations.

Crucially, household exposure and community prevalence enter the model as distinct quantities measured on different scales. Household exposure is derived from the cumulative daily viral load within a given household, inferred from a model of viral kinetics applied to each infected member, and capturing the intensity of within-household transmission pressure. Community prevalence is calculated as the proportion of population infected, which is purely based on binary PCR positivity, not viral load, and serves as a proxy for external risk of infection acquisition from the general community. It is worth noting that we monitor infections in 327 households, so that the infected members of a particular household will have a very small contribution to the overall community exposure which aggregates infections across 327 households. Further, as seen from timeline of infections in Supplementary Fig 4-6, household outbreaks are asynchronous in their epidemic timing, spanning 2-5 months depending on the year and circulating subtype. Hence there is more variability in the household outbreaks than in the aggregated community prevalence curve, which allows the model to disentangle the contribution from both internal and external forces of infections. Overall, the observed association between high community prevalence and elevated household infection risk therefore reflects the concurrence of two distinct transmission pressures, rather than a circular modeling assumption. We have added a comment in discussion to this effect and caveated the lack of broad community-based surveillance data (lines 439-447):

> *"Fifth, we did not have independent influenza surveillance data from the studied locales to infer community exposure, and instead aggregated PCR-identified infections across all studied households as a proxy for community FOI. In contrast, household FOI was measured by a distinct indicator, based on the cumulative daily viral load within a given household inferred from a viral kinetics model applied to each infected member. Overall, we believe the use of distinct FOI indicators for community and household exposures, fairly large number of households studied (n=327), and asynchronicity of individual outbreaks between households (Supplementary Figs 4-6), likely contributed to disentangling the role of these two infection sources."*

Minor comments:

1. In our previous review, we asked whether the authors accounted for false negative test results when fitting the model. From the authors' response, it is still unclear whether they accounted for false negatives; it sounds like the authors fitted the model to observed Ct values, thereby not accounting for false negatives? Please confirm.

We thank the reviewer for the opportunity to clarify this point. The inference framework implicitly accounts for false-negative test results through the observation model. Specifically, the inferred latent viral load trajectory ΔCt(t) is linked to the observed viral load via a noisy measurement likelihood function max(ΔCt(t), Normal(E[ΔCt(t)], sigma)). When the inferred viral load ΔCt(t) is small, the likelihood of observing a zero viral load (i.e., a false-negative test) is non-negligible due to measurement noise near the assay's detection limit.

Consequently, within the time window of an inferred infection episode, during which the inferred latent viral load remains strictly positive, the inference framework still allows for negative observations to occur probabilistically within the episode. In this sense, the model accommodates false negatives as a natural outcome of the observation process rather than treating observed negative results as error-free measurements.

2. Fig 1. Are the black dotted lines also smoothed? If so, this should be clarified in the caption (with the method of smoothing stated)?

Yes, the black dotted lines are smoothed. In the revised figure (which has been moved to Supplementary Fig 12), these lines now represent trends in the age-specific standard deviation smoothed by age group with the same method as the mean trend lines as using the same method improves interpretability and ensures consistency in how the age-related patterns are visualized. Specifically, both the mean and standard deviation curves are fitted using spline-based regression implemented via geom_smooth in ggplot2, with identical knot placement and spline degree. We have clarified this in the figure caption and updated Supplementary Materials Section 3 accordingly:

> *"The age-specific standard deviation, shown as the black dotted line, was calculated at each age and then smoothed using the same spline smoothing method, with identical knot placement and spline degree."*

3. One of the questions we asked about in our original review is where the priors were chosen from. While the authors have now attempted to explain this, we think that their explanation that they "were informed primarily by published viral kinetics models and relevant influenza human challenge studies [1,6,7]" is still quite vague. This could be made more precise; exactly which priors came from which study, and how were they chosen?

We thank the reviewer for this comment and agree that our original description of the prior specification was insufficiently precise. In the revised Supplementary Information, we now

explicitly describe how each prior was informed, and we clarify the rationale for the chosen prior means and variances as follows in the Supplementary Materials lines 109-127:

> *"These priors were informed by human challenge studies and household cohort studies [6–8]. In particular, we drew on a comprehensive review of 56 influenza challenge studies, which provides a consistent characterization of early viral shedding dynamics under controlled conditions [6]. Across the studies, viral shedding increased rapidly within the first day after challenge, peaked around day 2, and had an average duration of approximately 4.8 days with no significant variation between subtypes/lineages. The consistency in the proliferation stage across studies and subtypes/lineages motivated a prior mean of 2 days and prior variance of 1 day for the proliferation phase. However, the same review reported substantial variation in shedding clearance duration between studies and individuals within the same studies, which informed our choice of a relatively large variance of 3 days for the clearance phase. Further, challenge studies are restricted to healthy adults whereas household cohort studies suggest that in the excluded populations, particularly children, viral shedding can be longer and more heterogeneous [7,8]. Such studies also reported that influenza B viruses tend to exhibit slightly longer shedding durations than influenza A viruses. To account for these considerations, we specified priors for the mean clearance duration centered at 4 days for influenza A subtypes and 5 days for influenza B lineages. These priors were intended to be weakly informative, providing biologically plausible starting values while allowing the observed data to drive posterior inference.*
>
> *The prior on the maximum change in Ct value reflects the dynamic range of the RT-PCR assay and provides a weakly informative constraint on peak shedding intensity."*

4. Some of the text in the Supp Info is in equation font (e.g. "days" in "omega_p < 15 days") rather than in standard font.

We thank the reviewer for highlighting this and have revised all occurrences.

5. "To improve computational efficiency, constrained the parameters to biologically plausible upper bounds: $\omega < 15\ days$, $\omega < 30\ days$, and $\Delta Ct < 37$, as values beyond these thresholds are considered implausible for influenza viral kinetics". References would be useful here to support these choices. Also, we think you are missing the word "we" before constrained?

We thank the reviewer for finding this typo and agree that a reference would be useful to the reader to support the threshold choices. We have added a reference as follows in the Supplementary Materials lines 99-105:

> *"To improve computational efficiency, we constrained the parameters to biologically plausible upper bounds: $\omega_p < 15$ days, $\omega_c < 30$ days, and $\Delta Ct_{max} < 37$. Both human influenza challenge and household cohort studies have suggested the high end of shedding duration to fall around 8-10 days [6–8], though it has also been shown in A(H1N1)pdm09 that particularly severe infections might shed for over 27 days [9]. Thus, the constraints we used extend well beyond the typically observed durations to accommodate unusually long shedding episodes, while remaining computationally tractable."*

6. “higher B/Yamagata titers did associate with a reduce infection risk”. This should say “reduced”?

This typo has been fixed.

Reviewer #1 (Remarks on code availability):

We did not review the code in this review (we did access the code in our original review).

Reviewer #3 (Remarks to the Author):

The authors have fully addressed my comments in the revised manuscript. My pleasure to recommend the acceptance of this submission.

We sincerely thank the reviewer for their careful reading of our manuscript and for their positive assessment and recommendation for acceptance. We greatly appreciate the time and effort invested in providing constructive feedback.

Reviewer #4 (Remarks to the Author):

I co-reviewed this manuscript with one of the reviewers who provided the listed reports. This is part of the Nature Communications initiative to facilitate training in peer review and to provide appropriate recognition for Early Career Researchers who co-review manuscripts.

We thank the co-reviewer for their contribution and for participating in the peer review process as part of this initiative.

Reviewer #1 (Remarks to the Author):

Thank you to the authors for responding to our comments on the revised version of the manuscript. The authors have amended the major issues in the original version of the manuscript. We have two minor remaining comments; when these are addressed, we are content that the authors will have provided responses to all of our original points.

We thank the reviewer for their further constructive review of our manuscript and for highlighting these two remaining concerns. We have addressed the reviewers' comments in the revised manuscript and provide detailed, point-by-point responses below.

1. I am afraid that the description of the methods is (we think) still wrong. For example, in the equation for Delta(Ct) in line 88 of the Supplementary Information, when t is between t_p and t_p+omega_c, there is no dependence on t. We think this ought to be a linear change in Delta(Ct) (so must surely vary with t). I guess this is just a typo, but it points towards the authors needing to still check their description of the methods very carefully. We have not re-reviewed the manuscript in detail, so we would recommend that the authors please check the Supplementary Information again very carefully.

We are grateful to the reviewer for pointing out this typo and have added the appropriate terms to correct the equation. We have closely reviewed our methods description and are confident there are no further confusing typos or errors.

2. Based on the authors' response to our previous comment 6, it seems like the observation model is max(0, Normal(0,sigma)) far away from the peak viral load, including calendar times far preceding the infection episode. This means that, when there is no virus, there is still a substantial chance of a false positive test result (potentially even 50%, if any observed Ct is indicative of an infection). Is this correct? Apologies if we are misunderstanding something here. This assumption should at the very least be made very explicit in the manuscript, since a (possibly 50%?) chance of a false positive test result seems somewhat high.

We thank the reviewer for raising this important point and apologize that our previous description of the observation model was not sufficiently clear. We can see how the model specification, as written, could give the impression that observations far from an infection episode were treated as draws from the same viral-load observation process, which would imply an unrealistically high probability of false-positive RT-PCR results. This was not our intention, and we appreciate the opportunity to clarify this point.

The measurement error term in the viral-load observation model is intended to capture uncertainty in quantitative viral-load measurements conditional on an infection episode being

modeled, rather than the background false-positive probability of the RT-PCR assay at arbitrary calendar times. This uncertainty may reflect both assay-level variability and sampling-level variability. In particular, even for an actively infected individual, a nasopharyngeal swab may not always collect virus-containing material, especially early in infection when viral replication may be spatially localized within the upper respiratory tract. We therefore view this observation error primarily as a model for variability in measured viral load during and near an infection episode, not as a model for false-positive testing in periods when the individual is truly uninfected. To avoid applying this observation model to long periods in which individuals were unlikely to be infected, we did not fit the viral kinetics model to the entire testing history of each participant. Instead, as described in Supplementary Methods Section 1.1, infection episodes were first defined using RT-PCR measurements above the limit of detection. The viral kinetics model was then fitted only to observations within each identified episode, together with a two-week buffer period on either side. Negative RT-PCR results far from any positive infection episode were therefore not included in the likelihood for the viral kinetics model, and our approach does not imply a high false-positive probability at those time points.

We agree with the reviewer that this distinction should be made more explicit in the manuscript. We have revised the Supplementary Methods (lines 93-106) to clarify that the viral-load observation model is applied only within the restricted episode-level fitting window, and that it should not be interpreted as a model for the background false-positive probability of RT-PCR testing outside identified infection episodes. We have also clarified that negative tests far from identified infection episodes were treated as true negatives for the purpose of defining infection episodes and fitting windows.

Thanks again for amending your manuscript, and we hope that our reviews have been constructive. We believe that the manuscript is now much improved.

Reviewer #4 (Remarks to the Author):

I co-reviewed this manuscript with one of the reviewers who provided the listed reports. This is part of the Nature Communications initiative to facilitate training in peer review and to provide appropriate recognition for Early Career Researchers who co-review manuscripts.

We thank the co-reviewer for their contribution and for participating in the peer review process as part of this initiative.
